# Improved survival among colon cancer patients with increased differentially expressed pathways

**DOI:** 10.1186/s12916-015-0292-9

**Published:** 2015-04-08

**Authors:** Martha L Slattery, Jennifer S Herrick, Lila E Mullany, Jason Gertz, Roger K Wolff

**Affiliations:** Department of Internal Medicine, University of Utah School of Medicine, 383 Colorow, Salt Lake City, 84018 USA; Department of Oncological Sciences, Huntsman Cancer Institute, University of Utah School of Medicine, 1950 Circle of Hope, Salt Lake City, 84112 USA

**Keywords:** Colon cancer, Gene expression, *MYC*, RNAseq, *TGFB1*, *TP53*

## Abstract

**Background:**

Studies of colorectal cancer (CRC) have shown that hundreds to thousands of genes are differentially expressed in tumors when compared to normal tissue samples. In this study, we evaluate how genes that are differentially expressed in colon versus normal tissue influence survival.

**Methods:**

We performed RNA-seq on tumor/normal paired samples from 175 colon cancer patients. We implemented a cross validation strategy to determine genes that were significantly differentially expressed between tumor and normal samples. Differentially expressed genes were evaluated with Ingenuity Pathway Analysis to identify key pathways that were de-regulated. A summary differential pathway expression score (DPES) was developed to summarize hazard of dying while adjusting for age, American Joint Committee on Cancer (AJCC) stage, sex, and tumor molecular phenotype, i.e., MSI, *TP53*, *KRAS*, and CIMP.

**Results:**

A total of 1,138 genes were up-regulated and 695 were down-regulated. These de-regulated genes were enriched for 19 Ingenuity Canonical Pathways, with the most significant pathways involving cell signaling and growth. Of the enriched pathways, 16 were significantly associated with CRC-specific mortality, including 1 metabolic pathway and 15 signaling pathways. In all instances, having a higher DPES (i.e., more de-regulated genes) was associated with better survival. Further assessment showed that individuals diagnosed at AJCC Stage 1 had more de-regulated genes than individuals diagnosed at AJCC Stage 4.

**Conclusions:**

Our data suggest that having more de-regulated pathways is associated with a good prognosis and may be a reaction to key events that are disabling to tumor progression.

Please see related article: http://dx.doi.org/10.1186/s12916-015-0307-6.

**Electronic supplementary material:**

The online version of this article (doi:10.1186/s12916-015-0292-9) contains supplementary material, which is available to authorized users.

## Background

Cancer is a multifaceted disease, characterized by dysregulation of multiple genes in multiple pathways. Gene expression studies have demonstrated the extent to which genes are altered in tumors. Studies of colorectal cancer (CRC) have shown that hundreds to thousands of genes are differentially expressed in tumors when compared to normal tissue samples [1]. While gene expression studies are limited in their ability to distinguish the importance of individual genes that are differentially expressed in tumors; assessment of unique features of these genes and their associated pathways has shed light on important molecular differences between tumors. Studies have used gene expression data to classify tumor phenotypes as well as evaluate tumors with microsatellite instability [2-4]. Nannini et al. [5] summarized the utility of gene expression profiling into three categories: molecular diagnosis and disease classification; molecular characterization, including molecular staging, treatment prediction, and prognosis prediction; and target discovery that can lead to new treatment options.

In this study, we perform gene expression analysis with the goal of molecular characterization and prognosis prediction focused on identifying molecular pathways that are associated with outcome.

Our analysis takes a pathway approach with the goal of improving our knowledge of molecular diagnosis and prognosis prediction. We classify genes that are significantly differentially expressed into pathways relevant to the carcinogenic process for colon cancer. We evaluate the impact of these significant pathways on survival and disease stage. We believe that our sequential analytic approach will provide insight into the carcinogenic process and provide a better understanding of the biological significance of these pathways in colon cancer as well as insight into therapeutic possibilities.

## Methods

We used RNA from 175 tumor and normal pairs who were part of the Diet, Activity, and Lifestyle study, which is an incident, population-based, case-control study of colon cancer conducted in Utah, the Kaiser Permanente Medical Research Program (KPMRP), and the Twin Cities Metropolitan area. Normal tissue was taken from tissue adjacent to the tumor and was determined to be free of any tumor cells by the study pathologist. Tumor and non-tumor colonic tissue (subsequently referred to this as ‘normal’) for RNA extraction were available from the Utah and KPMRP sites. Cases had to have tumor registry verification of a first primary adenocarcinoma of the colon and diagnosed between October 1991 and September 1994. Tumor tissue was obtained for 97% of all Utah cases diagnosed and for 85% of all KPMRP study participants [6], including those who signed informed consent and those retrieved by local tumor registries and sent to study investigators without personal identifiers. The study was approved by the Institutional Review Board of the University of Utah and at KPMRP.

We have previously assessed these tumor samples for Tumor protein p53 (*TP53*) and *KRAS* mutations, the CpG island methylator phenotype (CIMP) using the classic panel [7], and MSI based on the mononucleotides *BAT26* and *TGFβRII* and a panel of 10 tetranucleotide repeats that were correlated highly with the Bethesda Panel [8]; our study was carried out prior to the Bethesda Panel development. We consider tumor molecular phenotype in our evaluation of survival since we have shown their association with survival [9,10]. Samples were selected to maximize numbers based on tumor molecular phenotype or *TP53*, *KRAS*, CIMP, and MSI tumor status.

### RNA processing

RNA was extracted from formalin-fixed paraffin-embedded tissues. We assessed slides and tumor blocks that were prepared over the duration of the study prior to the time of RNA isolation to determine their suitability. Older slides produced comparable RNA quality as more recent slides; RNA quality was not correlated with time lapse between slide preparation and mRNA preparation. The study pathologist reviewed slides to delineate cancer and normal tissue. Cells were dissected from 1 to 4 sequential sections on aniline blue stained slides using a hematoxylin and eosin slide for reference. Total RNA was extracted, isolated, and purified using the RecoverAll Total Nucleic Acid isolation kit (Ambion). RNA yields were determined using a NanoDrop spectrophotometer.

### Sequencing library preparation

Library construction was performed using the Illumina TruSeq Stranded Total RNA Sample Preparation Kit with Ribo-Zero. Briefly, ribosomal RNA was removed from 100 ng total RNA using biotinylated Ribo-Zero oligos attached to magnetic beads that are complimentary to cytoplasmic rRNA. Following purification, the rRNA-depleted sample is fragmented with divalent cations under elevated temperatures and primed with random hexamers in preparation for cDNA synthesis. First strand reverse transcription is accomplished using Superscript II Reverse Transcriptase (Invitrogen). Second strand cDNA synthesis is accomplished using DNA polymerase I and Rnase H under conditions in which dUTP is substituted for dTTP, yielding blunt-ended cDNA fragments in which the second strand contains dUTP. An A-base is added to the blunt ends as a means to prepare the cDNA fragments for adapter ligation and block concatemer formation during the ligation step. Adapters containing a T-base overhang were ligated to the A-tailed DNA fragments. Ligated fragments were PCR-amplified (13 cycles) under conditions in which the PCR reaction enables amplification of the first strand cDNA product, whereas attempted amplification of the second strand product stalls at dUTP bases and is therefore not represented in the amplified library. The PCR-amplified library was purified using Agencourt AMPure XP beads (Beckman Coulter Genomics). The concentration of the amplified library was measured with a NanoDrop spectrophotometer and an aliquot of the library was resolved on an Agilent 2200 Tape Station to define the size distribution of the sequencing library.

### Sequencing and data processing

Sequencing libraries (18 pM) were chemically denatured and applied to an Illumina TruSeq v3 single read flow cell using an Illumina cBot. Hybridized molecules were clonally amplified and annealed to sequencing primers with reagents from an Illumina TruSeq SR Cluster Kit v3-cBot-HS. Following transfer of the flowcell to an Illumina HiSeq instrument, a 50 cycle single-read sequence run was performed using TruSeq SBS v3 sequencing reagents. The single-end 50-base reads from the Illumina HiSeq2500 were aligned to a sequence database containing the human genome chromosomes (build GRCh37/hg19, February 2009, from UCSC Genome Bioinformatics [11]) plus all splice junctions generated using the USeq MakeTranscriptome application (version 8.8.1, [12]). Alignment was performed using Novoalign (version 2.08.01, [13]), which also trimmed any adapter sequence. Following alignment, genome alignments to splice junctions were translated back to genomic coordinates using the USeq SamTranscriptomeParser application. The resulting alignments were sorted and indexed using the Picard SortSam application (version 1.100, [14]). Aligned read counts for each gene were calculated using the pysam [15] and SAMtools [16]. A python script using the pysam library was given a list of the genome coordinates for each gene, and counts to the exons and UTRs of those genes were calculated. Gene coordinates were downloaded from the UCSC Genome Bioinformatics website [11].

Our data were compared to 51,041 molecular features in the gene table. Of these, 33,876 were excluded because of low to no expression in colon tissue or because they were non-coding or had no known function. We used the BioMart tool on the Ensembl website [17], to create a list of known regions linked to protein-coding genes from the human *GRCh38* gene annotation dataset. We included the 17,165 features on 17,141 genes involved in protein coding for data analysis.

### Statistical methods

Of the 197 initial tumor/normal pairs, 5 subjects failed quality control based on the low number of sequence counts for both tumor and normal, and 17 were dropped because either the normal or tumor pair failed quality control, leaving 175 subjects with high quality data for inclusion in the analysis. From this pool of subjects, we randomly assigned people to group “A” or “B” to cross-validate findings pertaining to differentially expressed genes. To prevent biasing the data towards those genes differentially expressed among people who were alive since our population had more alive individuals than those who had died, we used balanced groups of people who died and matched people who were alive to those who died based on age category and sex. To assess differences in overall tumor vs. normal tissue expression level, we performed a paired comparison, resampling the data 20 times, and permuting the data 1,000 times using the program SAMseq implemented in the ‘samr’ package of R [18] for each group [19,20]. Fold-change was calculated as the ratio of the means of tumor expression to the means of normal expression. Further bioinformatics analysis and survival and stage analysis included the entire sample of eligible participants, analyzing only those features that were significantly differentially expressed in both groups A and B with a *P* <0.05 and over a two-fold change in expression level between normal and tumor tissue.

Bioinformatics analysis was performed on the list of Ensemble IDs found to be significantly differentially expressed between tumor and normal tissue at a *P* value of <0.05 with a two-fold change in both Group A and Group B. Our goal in the bioinformatics analysis was to identify key pathways that were deregulated in colon cancer and assess the potential impact of those pathways on survival. We utilized QIAGEN’s Ingenuity Pathway Analysis (IPA) [21] with the following criteria: A total of 1,138 unregulated features and 695 down-regulated feature Ensembl IDs were uploaded to IPA, and all but five were successfully mapped to Ingenuity. The five unmapped IDs were: ENSG00000184682, ENSG00000214999, ENSG00000251184, ENSG00000244255, ENSG00000167046; the first two IDs are for genes that were down-regulated and the last three are IDs for genes that were up-regulated. The IPA settings were as follows for General Settings: only genes from Ingenuity Knowledge Base were used, and both indirect and direct relationships were considered; for Network: both causal and interaction networks were included, for Interaction we included endogenous chemicals and we used the defaults set by Ingenuity, 35 molecules per network and 25 networks per analysis; for Data Sources: all data sources were used; for Confidence: only experimentally observed relationships were considered; for Species: all species were included; for Tissue: no specific tissue was selected; for Mutations: all mutations were included. For Species and Tissue selections, the ‘stringent filter’ option was selected. For the Canonical Pathways Analysis the selected scoring method was the B-H Multiple Testing Correction *P* value and for this method all pathways that score between 0 and 6.47 are displayed.

Genes that were significantly differentially expressed were grouped into pathways based on IPA summary data. This involved several steps. First, individuals were given a score for each differentially expressed gene depending on their level of differential expression: 1 was assigned to individuals in the bottom quartile of the distribution of differential expression (i.e., closer to normal), 2 was assigned to individuals whose differential expression for the gene was between the 25^th^ and 75^th^ percentile of the population differential expression, and 3 was assigned to those whose tumors were in the top (>75^th^ percentile) level of differential expression. Next, individual differential gene expression scores (DGES) were summed to obtain a differential pathway expression score (DPES) for genes in the pathways that were statistically significant after adjusting for multiple comparisons as described by Benjamini and Hochberg [22]. A higher DPES correlates with more genes being differentially expressed. DPES were categorized into tertiles for survival analysis using a Cox Proportional Hazard model, adjusting for age, sex, American Joint Committee on Cancer (AJCC) stage, and tumor molecular phenotype (*TP53*, *KRAS*, MSI, and CIMP) using SAS 9.4 (SAS Institute, Cary, NC, USA). We report hazard ratios (HR) and 95% confidence intervals (CI) associated with survival. Survival data were obtained from local tumor registries and reported as months survived from date of diagnosis to date of last contact or lost to follow-up. We report HR associated with CRC death where other causes of death were censored. Similar categories were used to evaluate mean DPES expression levels across AJCC stages of 1 through 4.

## Results

We analyzed gene expression in tumor/normal paired samples from 175 colon cancer patients using RNA-seq. Of these tumors, 47.9% were proximal and 52.1% were distal colon and were similar for both Group A and B (Table 1). Evaluation of tumor molecular phenotype showed that 25.7% were CIMP high, 18.3% were MSI, 27.4% were *KRAS* mutated, and 44% were *TP53* mutated. The average age of the study participants included in these analysis was 65.2 years. Groups A and B were used to determine if significant differentially expressed genes were similar for most variables.

**Table 1:** **Description of the study population**

		**Total population**		**Group A** ^**1**^		**Group B**	
		**n**	**%**	**n**	**%**	**n**	**%**
Sex	Male	94	53.7	18	48.6	14	34.1
	Female	81	46.3	19	51.4	27	65.9
Center	Kaiser	106	60.6	22	59.5	23	56.1
	Utah	69	39.4	15	40.5	18	43.9
Site	Proximal	78	47.9	16	45.7	21	53.8
	Distal	85	52.2	19	54.3	18	46.2
Vital status	Alive	104	59.4	15	40.5	24	58.5
	CRC death	39	22.3	22	59.5	17	41.5
	Other death	17	9.7				
	Unknown	15	8.6				
AJCC stage	1	40	23.0	6	16.2	8	19.5
	2	56	32.2	9	24.3	14	34.1
	3	57	32.8	15	40.5	9	22.0
	4	21	12.1	7	18.9	10	24.4
*TP53*	Non-mutated	98	56.0	22	59.5	26	63.4
	Mutated	77	44.0	15	40.5	15	36.6
*KRAS*	Non-mutated	127	72.6	26	70.3	31	75.6
	Mutated	48	27.4	11	29.7	10	24.4
MSI	Stable	143	81.7	30	81.1	33	80.5
	Unstable	32	18.3	7	18.9	8	19.5
CIMP	Low	130	74.3	27	73.0	27	65.9
	High	45	25.7	10	27.0	14	34.1
		Mean	STD	Mean	STD	Mean	STD
Age		65.2	10.2	64.8	10.9	63.2	11.3

^1^Groups A and B were randomly selected to determine differentially expressed genes for further analysis.

AJCC, American Joint Committee on Cancer; CIMP, CpG island methylator phenotype; MSI, Microsatellite instability; STD, Standard deviation.

Of the 17,141 genes evaluated, using the parameter of two-fold change in addition to a *P* <0.05 for both groups, 1,138 were significantly up-regulated and 695 were significantly down-regulated between tumor and normal tissue. Of the 1,833 genes identified as having significant differential expression, 1,567 were linked to Cancer, while 1,290 were linked to Gastrointestinal Diseases in IPA. The main molecular and cellular functions that these genes contributed to were cellular growth and proliferation (715 genes with gene enrichment *P* values of 1.89 × 10^-43^ to 2.01 × 10^-05^), cell death and survival (632 genes with gene enrichment *P* values from 5.39 × 10^-33^ to 20.4 × 10^-05^), cell cycle (316 genes with enrichment *P* values from 6.25 × 10^-26^ to 2.19 × 10^-05^), cellular movement (415 genes with enrichment *P* values of 1.51 × 10^-21^ to 2.22 × 10^-05^), and cellular assembly and organization (275 genes with enrichment *P* values of 2.79 × 10^-20^ to 7.75 × 10^-06^). Additional file 1 has a complete list of differentially expressed genes analyzed and their level of expression.

**Figure 1:**
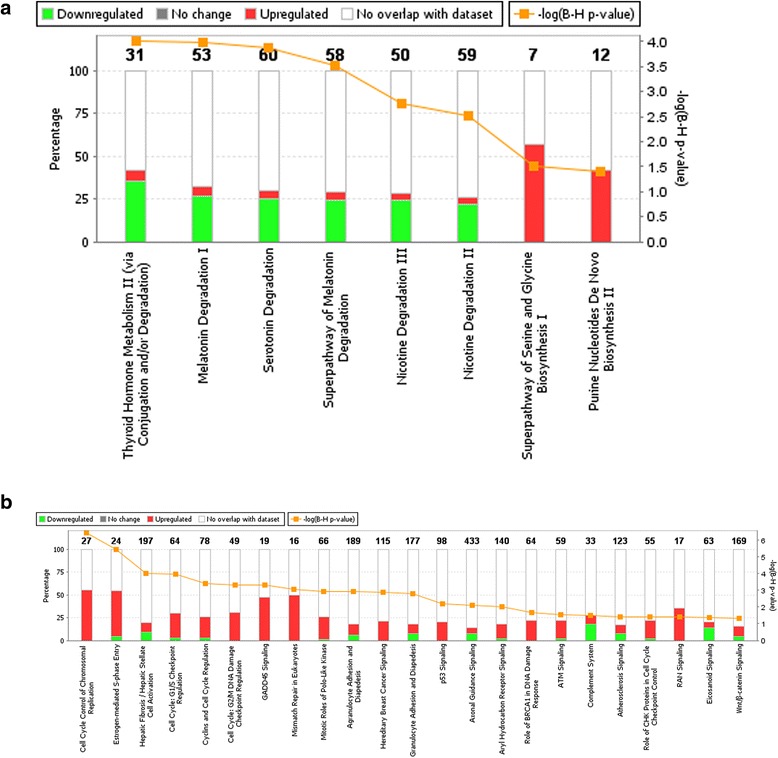
**Significant canonical pathways identified from IPA.** The pathways were statistically significant at the 0.05 level after adjustment for multiple comparisons. **(a)** Metabolic pathway. **(b)** Signaling pathway.

We also linked these genes to major canonical pathways in IPA, summarized in Figure 1; green refers to down-regulated genes and red to up-regulated genes within the pathway. Our significant differentially expressed genes were significantly enriched in 30 pathways (Additional file 2 shows genes in our data that were associated with these pathways). For the most part, the pathways with the majority of genes being significantly down-regulated were in metabolic pathways (Thyroid Hormone Metabolism, Malatonin Degredation I, Seratonin Degradation, Superpathway of Melatonin Degradiation, and Nicotine Degradation III and II). The other two metabolic pathways, Superpathway of Serine and Glycine Biosynthesis and Purine Nucleotides De Novo Biosynthesis, were only up-regulated. The other 22 pathways that were differentially expressed were signaling pathways, where the majority of genes were up-regulated. Exceptions to this were Complement System and Eicosanoid Signaling where the majority of de-regulated genes were down-regulated.

In order to analyze a pathway’s prognostic value, we constructed a DPES that captures the extent to which a pathway is de-regulated in a given individual. Evaluation of DPES with CRC-specific mortality showed significant reduced mortality as the number of differentially expressed genes increased for several signaling pathways (Table 2). Of the pathways significantly enriched for genes that were differentially expressed between tumor and normal tissue in our data, 16 were significantly associated with CRC-specific mortality. One of these, Purine Nucleotides de Novo Biosynthesis II was a metabolic pathway that was up-regulated, while the other 15 were signaling pathways. In all instances, a higher DPES (T3) was associated with better survival. Similar associations were observed when looking at expression in tumors only. Additional file 3: Figures S1, Additional file 4: Figure S2, and Additional file 5: Figure S3 show Kaplan-Meier curves for the first three pathways listed in Table 2.

**Table 2:** **Associations between the differential gene expression score and colorectal cancer-specific mortality**

**Pathways**	**T1 HR** ^**1**^ ** (referent)**	**T2 HR** ^**1**^	**(95% CI)**	**T3 HR** ^**1**^	**(95% CI)**
**Cell Cycle Control of Chromosomal Replication**	**1.00**	**0.36**	**(0.17, 0.77)**	**0.37**	**(0.16, 0.87)**
**Estrogen-mediated S-phase Entry**	**1.00**	**0.38**	**(0.18, 0.81)**	**0.35**	**(0.15, 0.81)**
Thyroid Hormone Metabolism II	1.00	1.26	(0.57, 2.78)	1.60	(0.71, 3.63)
Hepatic Fibrosis / Hepatic Stellate Cell Activation	1.00	0.73	(0.33, 1.58)	0.75	(0.34, 1.65)
Cell Cycle: G1/S Checkpoint Regulation	1.00	0.39	(0.19, 0.81)	0.44	(0.18, 1.07)
Melatonin Degradation I	1.00	1.58	(0.71, 3.54)	1.44	(0.66, 3.12)
Serotonin Degradation	1.00	1.29	(0.58, 2.87)	1.48	(0.68, 3.23)
Superpathway of Melatonin Degradation	1.00	1.58	(0.71, 3.54)	1.44	(0.66, 3.12)
**Cyclins and Cell Cycle Regulation**	**1.00**	**0.41**	**(0.20, 0.87)**	**0.28**	**(0.12, 0.67)**
**Cell Cycle: G2/M DNA Damage Checkpoint Regulation**	**1.00**	**0.35**	**(0.16, 0.75)**	**0.34**	**(0.15, 0.78)**
**GADD45 Signaling**	**1.00**	**0.34**	**(0.15, 0.74)**	**0.36**	**(0.15, 0.82)**
**Mismatch Repair in Eukaryotes**	**1.00**	**0.51**	**(0.23, 1.10)**	**0.38**	**(0.17, 0.88)**
Mitotic Roles of Polo-Like Kinase	1.00	0.54	(0.26,1.16)	0.43	(0.19, 1.01)
Agranulocyte Adhesion and Diapedesis	1.00	0.68	(0.31, 1.49)	0.59	(0.27, 1.32)
**Hereditary Breast Cancer Signaling**	**1.00**	**0.40**	**(0.19, 0.85)**	**0.33**	**(0.14, 0.78)**
Granulocyte Adhesion and Diapedesis	1.00	0.73	(0.34, 1.59)	0.54	(0.24, 1.23)
Nicotine Degradation III	1.00	1.59	(0.73, 3.47)	1.59	(0.68, 3.70)
Nicotine Degradation II	1.00	1.81	(0.81, 4.05)	1.93	(0.86, 4.34)
***TP53*** **Signaling**	**1.00**	**0.39**	**(0.18, 0.84)**	**0.34**	**(0.14, 0.78)**
Axonal Guidance Signaling	1.00	0.49	(0.21, 1.11)	0.86	(0.41, 1.81)
**Aryl Hydrocarbon Receptor Signaling**	**1.00**	**0.42**	**(0.20, 0.92)**	**0.36**	**(0.16, 0.80)**
**Role of** ***BRCA1*** **in DNA Damage Response**	**1.00**	**0.46**	**(0.22, 0.97)**	**0.33**	**(0.14, 0.81)**
**ATM Signaling**	**1.00**	**0.43**	**(0.20, 0.92)**	**0.34**	**(0.15, 0.80)**
Complement System	1.00	1.28	(0.56, 2.93)	1.01	(0.41, 2.51)
**Superpathway of Serine and Glycine Biosynthesis I**	**1.00**	**0.43**	**(0.18, 1.03)**	**0.30**	**(0.13, 0.69)**
Atherosclerosis Signaling	1.00	0.70	(0.32, 1.56)	0.88	(0.41, 1.87)
**Role of CHK Proteins in Cell Cycle Checkpoint Control**	**1.00**	**0.44**	**(0.21, 0.90)**	**0.35**	**(0.14, 0.86)**
**RAN Signaling**	**1.00**	**0.36**	**(0.16,0.81)**	**0.39**	**(0.17, 0.86)**
**Purine Nucleotides De Novo Biosynthesis II**	**1.00**	**0.36**	**(0.17, 0.77)**	**0.26**	**(0.10, 0.66)**
Eicosanoid Signaling	1.00	0.97	(0.44,2.14)	0.91	(0.40, 2.08)
**Wnt/ß-catenin Signaling**	**1.00**	**0.38**	**(0.17, 0.85)**	**0.44**	**(0.20, 0.99)**

^1^Hazard ratios (HR) and 95% confidence intervals (CI) adjusted for age, sex, AJCC stage, *TP53*, and *KRAS* mutations, CIMP, and MSI. T1, Tertile 1 and Referent group; T2, Tertile 2; T3, Tertile 3; tertiles are based on the distribution of the Differential Pathway Expression Score with higher tertile having greater differential expression. Bold text highlights those pathways that were statistically significantly associated with colorectal cancer-specific survival.

We further assessed mean levels of DPES by stage (Table 3) and observed that those diagnosed at AJCC Stage 1 had more differentially expressed genes than individuals who were diagnosed at AJCC Stage 4. This trend was present for most pathways, although the majority did not reach statistical significance, which may be indicative of limited power from the few individuals with an AJCC Stage 4 tumor.

**Table 3:** **Associations between significant pathways and AJCC stage**

		**Stage 1**		**Stage 2**		**Stage 3**		**Stage 4**		
**Pathway**	**# Features (Genes)**	**Mean** ^**1**^	**STD**	**Mean**	**STD**	**Mean**	**STD**	**Mean**	**STD**	***P*** **value** ^**2**^
Cell Cycle Control of Chromosomal Replication	15	31.0	9.4	30.5	8.8	30.1	7.4	27.0	8.6	0.11
Estrogen-mediated S-phase Entry	13	27.1	7.7	26.2	6.8	26.2	5.8	23.3	6.9	0.06
Thyroid Hormone Metabolism II	12	24.3	5.5	24.3	5.5	24.7	5.7	22.8	5.6	0.33
Hepatic Fibrosis/Hepatic Stellate Cell Activation	39	78.4	14.0	78.5	13.1	80.7	14.1	73.1	14.3	0.17
Cell Cycle: G1/S Checkpoint Regulation	19	38.8	10.4	38.2	9.7	38.6	8.3	34.6	9.7	0.13
Melatonin Degradation I	16	32.1	6.7	32.7	6.7	32.6	6.8	30.7	7.1	0.45
Serotonin Degradation	17	34.7	7.3	34.5	7.1	34.8	7.7	32.5	7.5	0.28
Superpathway of Melatonin Degradation	16	32.1	6.7	32.7	6.7	32.6	6.8	30.7	7.1	0.45
Cyclins and Cell Cycle Regulation	20	41.2	10.9	40.3	10.1	40.4	8.6	36.3	9.9	0.09
Cell Cycle: G2/M DNA Damage Checkpoint Regulation	15	31.2	9.8	30.1	8.7	30.0	7.9	26.8	9.0	0.09
GADD45 Signaling	9	18.9	5.6	18.3	5.2	17.8	4.4	16.4	5.2	0.11
Mismatch Repair in Eukaryotes	8	16.9	4.8	16.2	4.6	15.9	4.1	14.4	5.1	0.06
Mitotic Roles of Polo-Like Kinase	17	35.5	9.6	34.6	9.0	34.0	8.0	30.9	8.6	0.07
Agranulocyte Adhesion and Diapedesis	34	70.6	11.4	68.3	9.8	70.5	11.2	65.6	13.3	0.13
Hereditary Breast Cancer Signaling	24	50.1	13.9	48.7	13.3	48.2	12.2	43.9	13.4	0.10
Granulocyte Adhesion and Diapedesis	32	66.3	10.6	64.8	9.5	66.8	10.6	61.3	12.6	0.10
Nicotine Degradation III	13	25.8	5.9	26.8	5.9	26.3	5.9	24.9	6.2	0.59
Nicotine Degradation II	14	27.8	6.2	28.8	6.4	28.2	6.4	26.9	6.6	0.59
p53 Signaling	20	40.8	11.2	40.6	10.4	40.7	9.7	36.4	11.0	0.15
Axonal Guidance Signaling	60	121.2	20.6	121.9	20.7	122.5	21.1	113.5	22.9	0.18
Aryl Hydrocarbon Receptor Signaling	25	51.4	13.5	50.1	11.6	50.6	10.7	46.3	12.8	0.16
Role of BRCA1 in DNA Damage Response	14	29.5	8.2	28.6	7.7	27.9	7.3	25.2	7.9	0.05
ATM Signaling	13	27.1	7.5	26.6	7.1	25.6	6.3	23.8	7.0	0.10
Complement System	10	20.7	3.3	19.7	3.4	20.3	3.4	19.3	3.6	0.12
Superpathway of Serine and Glycine Biosynthesis I	4	8.1	2.3	8.0	2.3	8.2	2.0	7.5	2.6	0.34
Atherosclerosis Signaling	20	40.4	6.9	39.9	6.1	41.7	6.7	38.4	8.1	0.32
Role of CHK Proteins in Cell Cycle Checkpoint Control	12	25.0	6.8	24.5	6.4	24.0	5.4	21.4	6.4	0.05
RAN Signaling	6	12.6	4.1	11.8	3.7	12.1	3.4	11.0	4.0	0.15
Purine Nucleotides De Novo Biosynthesis II	5	10.2	3.3	10.0	3.1	10.2	3.0	9.2	3.2	0.26
Eicosanoid Signaling	13	26.1	4.0	26.1	4.3	27.0	4.7	25.0	5.0	0.37
Wnt/ß-catenin Signaling	26	52.7	10.6	52.3	9.5	53.2	10.0	48.9	10.9	0.19

^1^Mean and SD values are from Differential Pathway Expression Score; ^2^ *P* values compares differences between Stages 1 and 4.

**Figure 2:**
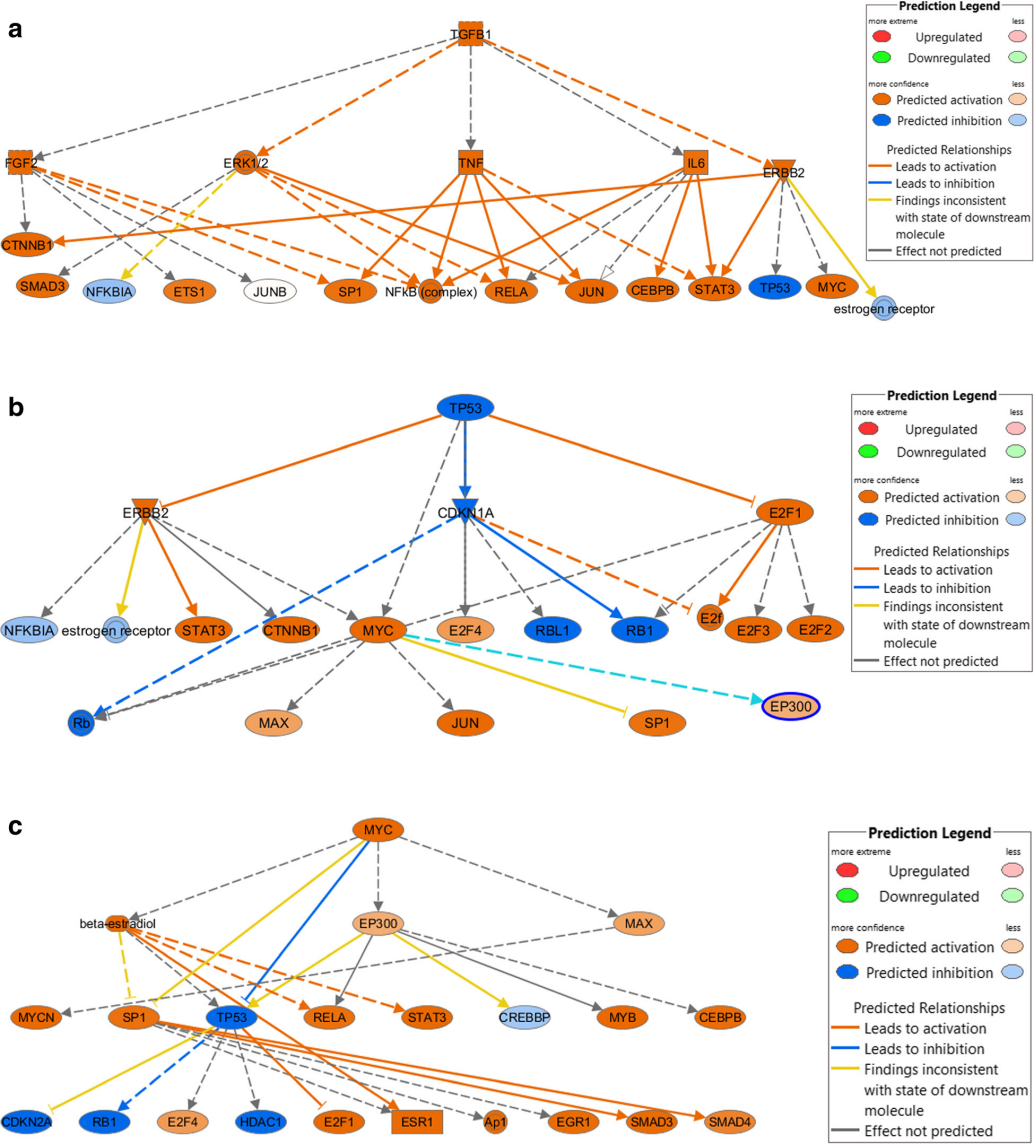
**Major upstream regulators, TGFB1, MYC, and TP53 enriched by differentially expressed genes in our dataset. (a)** *TGFB1* upstream regulator of networks where the gene enrichment *P* values for differential expression was highly significant. **(b)** *MYC* primary upstream regulator of networks where the gene enrichment *P* value for differential expression was highly significant. **(c)** *TP53* primary upstream regulator of networks where the gene enrichment *P* value for differential expression was highly significant.

To help interpret these results, we assessed upstream regulators using IPA. The top upstream of molecules associated with the de-regulated genes in our data were *TGFB1* (*P* = 8.14 × 10^-46^), beta-estradiol (*P* = 1.21 × 10^-41^), *TP53* (*P* = 1.90 × 10^-38^), *CDKN1A* (*P* = 1.41 × 10^-37^), and *MYC* (*P* = 4.59 × 10^-36^) (Table 4). It is interesting to note that *TP53*, which was predicted to be inhibited in our data, was actually up-regulated, although only significant in Group A and therefore not included in the analysis. This suggests that an indirect relationship with other molecules could have resulted in this shift in expected activation. The major network regulators regulated by the *TGFB1*, *TP53*, and *MYC* are shown in Figure 2a, b, and c, respectively. There were 20 mechanistic network regulators for *TGFB1*, 20 for *TP53*, and 22 for *MYC* that were significantly enriched in our data based on the number of differentially expressed genes belonging directly and indirectly to these networks (Additional file 6: Table S3 shows genes in our data that were directly or indirectly regulated by these pathways).

**Table 4:** **Top upstream regulators of genes significantly differentially expressed in dataset**

**Upstream regulator**	**Molecule type**	**Predicted activation state**	***P*** **value of overlap**	**Genes in dataset (Number of regulators from data in network)**
*TGFB1*	Growth factor	Activated	8.14 × 10^-46^	743 (20)
Beta-estradiol	Chemical drug	Activated	1.21 × 10^-41^	779 (24)
*TP53*	Transcription regulator	Inhibited	1.41 × 10^-37^	616 (20)
*CDKN1A*	Kinase	Inhibited	4.35 × 10^-35^	485 (14)
*MYC*	Transcription regulator	Activated	4.59 × 10^-36^	660 (22)
Calcitriol	Chemical drug	Inhibited	4.51 × 10^-34^	538 (24)
*E2F1*	Transcription regulator	Activated	1.71 × 10^-30^	397 (15)

*CDKN1A*, Cyclin-dependent kinase inhibitor 1A; E2F1, E2F transcription factor 1; MYC, v-myc avian myelocytomatosis viral oncogene homolog; *TGFB1*, Transforming growth factor beta 1; *TP53*, Tumor protein p53.

## Discussion

Our data illustrate the complexity of colon cancer and the extent to which genes are significantly differentially expressed in tumors. These differentially expressed genes are associated with many pathways and functions, many of which are associated with survival and provide insight into the broader carcinogenic process. Our data suggest that tumors with the most differentially expressed genes in key pathways are associated with better survival and less advanced disease stage. The differentially expressed genes were associated with upstream regulators that have previously been associated with colon cancer, such as *TGFB1*, *MYC*, and *TP53.* In our data, these genes were activated and influenced the downstream genes and pathways they regulate. It appears that activation of these pathways improves prognosis. This information could potentially be utilized to help determine biomarkers for treatment modalities that may influence survival.

Disruption of multiple biological pathways is a hallmark feature of the tumors. Our data illustrates the number of key pathways involved in the carcinogenic process and the number of genes showing significant differential gene expression in both test and validation data sets, with 1,138 features being significantly up-regulated and 695 being significantly down-regulated in both groups. Many of these pathways are comprised of genes involved in cell growth, differentiation, and apoptosis. One of the top pathways that showed significant differential expression in our data was Cell Cycle Control of Chromosomal Replication (55.6% of genes in pathway were significantly differentially expressed in our data). Additionally, in the Cell Cycle: G1/S Checkpoint Regulation pathway almost 30% of genes in the pathway were de-regulated in our data. These observations were further supported by the number of genes involved in key molecular and cellular functions of cellular growth and proliferation (715 genes), cell death and survival (632 genes), cell cycle (316 genes), cellular assembly and organization (275 genes), and cellular movement (415 genes). Also of interest was the observation that 54.2% of genes that showed significant differential expression were in the Estrogen-mediated S-phase Entry Canonical Pathway, which was the second most enriched pathway from our data. Estrogen status and hormone therapy have been shown to reduce risk of developing colon cancer and have been associated with better survival after diagnosis with colon cancer [23,24].

The major finding from this study is that better survival is seen in patients with more differential gene expression and DPES after adjusting for tumor stage and tumor molecular phenotype of *TP53*, *KRAS*, CIMP, and MSI. This observation was further supported by the higher DPES being observed for those who were diagnosed at AJCC Stage 1 vs. Stage 4. This implies that genes are activated as part of a cell response mechanism potentially to promote apoptosis and decrease tumor growth. Individuals who are able to initiate this response have better survival and tumors that are less likely to advance. This observation also supports the concept that genes downstream respond to upstream events that may be driving the carcinogenic process. While the phenomena of having more differentially expressed genes being associated with better survival could seem counterintuitive, given disrupted genes and gene regulation are a hallmark feature of tumors, others have noted similar observations that tumors from patients who live longer after cancer diagnosis have more differentially expressed genes [25]. However, replication of these findings in other similar datasets is needed.

To help interpret the results, we further evaluated the top upstream regulators of pathways where we observed significant enrichment of differentially expressed genes in our data. Three of the top upstream regulators were *TGFB1*, *MYC*, and *TP53*; these regulators are frequently associated with colon cancer. *TGFB1* is a growth factor that has been linked to apoptosis through multiple mechanisms [26,27] and is required to maintain homeostasis between apoptosis and cell growth. Multiple pathways, including MAPK signaling, SMAD, and JNK are linked to *TFGB1* and its role on apoptosis. If *TGFB1* is up-regulated, as it was in our data, it is possible it activates signaling cascades that lead to cell death, which in turn would improve survival. *MYC* regulates many functions, some that promote tumor growth, while others promote apoptosis [28]. MYC expression has been associated with improved survival in the absence of *TP53* mutations in one study [29]. While we attempted to confirm this association, there were too few deaths among those with *TP53* mutations to estimate the association; however, an inverse association was observed for higher MYC differential expression among those with non-*TP53* mutated tumors. In our data, *MYC* primarily regulated transcription factors, playing a key role in downstream gene regulation, through both direct and indirect gene targeting. The importance of *MYC* as a regulator of transcriptional activation and repression in CRC also was noted by the Cancer Genome Atlas Network [30]. Likewise, *TP53*, a frequently mutated gene in colon cancer [31], was significantly up-regulated in our data, and genes for which *TP53* was an upstream regulator were differentially expressed in our data. As a tumor suppressor gene, *TP53* is involved in apoptosis, so it is reasonable that enrichment of differentially expressed genes in this pathway could significantly have a favorable effect on survival.

There are several possible explanations for our observations that pathways with increased differential are associated with better survival. Large gene expression changes could be due to cell response to over-proliferation and attempt to shut down. Increased gene expression changes could be destabilizing for the tumor and lead to better overall survival. Alternatively, expression changes could be the reaction to immune signaling and infiltration.

This study has several strengths and limitations. First, we have a rich dataset in which to examine gene expression profiles of colon tumors. Because we had tumor and normal paired samples, we were able to evaluate differential gene expression. Although our normal tissue was from colonic tissue adjacent to the tumor, it could have undergone gene changes and therefore not truly ‘normal’. However, it is the only practical tissue available for comparison. Our sample was large enough that we were able to use both a test and retest set of data to validate findings and only evaluate those differentially expressed genes with survival that were differentially expressed in both groups. Our pathway approach enabled us to group genes together based on canonical pathways rather than evaluate genes individually. Other pathways and genes that were not identified in IPA could be important. Additionally, it is important to recognize that other genes or specific pathways could detrimentally influence survival. While a linear trend with increasing de-regulated genes would be expected, frequently this was not the case. Often, the largest drop in risk was going from tertile 1 to tertile 2. Additionally, we are only able to look at gene expression and not actual protein expression.

In summary, our data suggest that having more genes differentially expressed in colon tumors compared to normal tissues improves survival and the likelihood of being diagnosed at a less advanced disease stage. This may be the signature of a cellular response mechanism and an ongoing challenge is to identify the key factors that stimulate the activation of important upstream genes that are required to mount a cellular response to the initial drivers in the carcinogenic process and to understand the cellular response to those initiating events.

## Conclusions

Our data suggest that having more de-regulated pathways is associated with a good prognosis and may be a reaction to key events that are disabling to tumor progression. This observation is re-enforced by the observation that people diagnosed at AJCC Stage 1 had more de-regulated genes than those diagnosed at AJCC Stage 4. These findings need confirmation in other studies.

## Additional files

Additional file 1:
**Genes up and down regulated in both Group A and Group B.**
Additional file 2:
**Major Canonical Pathway and related gene enrichment and key molecules; pathways have a significant gene enrichment after adjustment for multiple comparisons.**
Additional file 3: Figure S1. Kaplan-Meier curve for de-regulated genes in the Thyroid Hormone Metabolism II in the IPA Canonical Pathway. Additional file 4: Figure S2. Kaplan-Meier curve for de-regulated genes in the Cell Cycle Control of Chromosomal Replication Pathway IPA Canonical Pathway. Additional file 5: Figure S3. Kaplan-Meier curve for de-regulated genes in the Estrogen-mediated S-phase Entry IPA Canonical Pathway. Additional file 6: Table S3. Description of major upstream regulators and their targets in our data.

## Abbreviations

AJCC: American joint committee on cancer
CI: Confidence intervals
CIMP: CpG Island Methylator Phenotype
CRC: Colorectal cancer
DGES: Differential gene expression score
DPES: Differential pathway expression score
HR: Hazard ratios
IPA: Ingenuity pathway analysis
KPMRP: Kaiser Permanente Medical Research Program
MSI: Microsatellite instability
MYC: v-myc avian myelocytomatosis viral oncogene homolog
*TGFB1*: Transforming growth factor beta 1
*TP53*: Tumor protein p53
