# Supplementary material for: Improved survival among colon cancer patients with increased differentially expressed pathways
Source: BMC Med. 2015 Apr 8;13:75. doi: 10.1186/s12916-015-0292-9 (PMC4389992; doi:10.1186/s12916-015-0292-9)
Supplement: Additional file 1: — Genes up and down regulated in both Group A and Group B. [file 12916_2015_292_MOESM1_ESM.docx]

| Online Supplement 1. Genes up and down regulated in both Group A and Group B | | | | | | | | | | | |  |  |
| --- | --- | --- | --- | --- | --- | --- | --- | --- | --- | --- | --- | --- | --- |
| UP-Regulated (1138 features) | | | |  |  | DOWN-Regulated (695 features) | | | | | |  |  |
|  | Average Expression | | Fold Change |  |  | Gene | Average Expression | | | | Fold Change |  |  |
| Name | Normal | Tumor |  | P Value |  | Name | Normal | | Tumor | |  | P Value |  |
| *SLCO1B3* | 0.03 | 0.97 | 29.12 | 5.43E-05 |  | *CLDN8* | 0.61 | | 0.00 | | 0.00 | 3.42E-05 |  |
| *NOTUM* | 0.02 | 0.58 | 27.64 | 7.89E-03 |  | *TMIGD1* | 1.01 | | 0.01 | | 0.01 | 3.35E-05 |  |
| *SLCO1B3* | 0.03 | 0.66 | 26.44 | 7.26E-05 |  | *ZG16* | 8.04 | | 0.12 | | 0.02 | 3.35E-05 |  |
| *INS-IGF2* | 0.23 | 5.97 | 26.05 | 6.63E-04 |  | *GUCA2B* | 1.04 | | 0.02 | | 0.02 | 4.69E-05 |  |
| *IGF2* | 0.58 | 13.77 | 23.89 | 1.60E-02 |  | *MS4A12* | 2.53 | | 0.05 | | 0.02 | 3.35E-05 |  |
| *CST1* | 0.06 | 1.18 | 18.52 | 6.19E-05 |  | *AQP8* | 2.74 | | 0.06 | | 0.02 | 3.35E-05 |  |
| *LY6G6D* | 0.05 | 0.81 | 17.30 | 1.94E-02 |  | *OTOP2* | 0.20 | | 0.01 | | 0.03 | 4.89E-04 |  |
| *DUSP27* | 0.02 | 0.39 | 16.97 | 3.14E-02 |  | *PYY* | 0.75 | | 0.02 | | 0.03 | 3.35E-05 |  |
| *SLCO1B7* | 0.01 | 0.23 | 15.76 | 1.96E-02 |  | *CA4* | 2.64 | | 0.08 | | 0.03 | 3.35E-05 |  |
| *KLK10* | 0.08 | 1.22 | 15.21 | 3.35E-05 |  | *CD177* | 1.87 | | 0.06 | | 0.03 | 3.35E-05 |  |
| *CXCL11* | 0.03 | 0.49 | 14.92 | 3.65E-03 |  | *SCNN1B* | 1.25 | | 0.04 | | 0.03 | 3.35E-05 |  |
| *DSG3* | 0.03 | 0.38 | 12.86 | 2.27E-04 |  | *GUCA2A* | 7.94 | | 0.29 | | 0.04 | 3.35E-05 |  |
| *MEGT1* | 0.06 | 0.71 | 12.69 | 1.07E-02 |  | *ADH1C* | 4.93 | | 0.21 | | 0.04 | 3.35E-05 |  |
| *LY6G6F* | 0.06 | 0.74 | 12.10 | 1.17E-02 |  | *UGT2B17* | 3.50 | | 0.16 | | 0.05 | 3.35E-05 |  |
| *DPEP1* | 0.35 | 4.11 | 11.61 | 2.62E-04 |  | *CA2* | 11.11 | | 0.51 | | 0.05 | 3.35E-05 |  |
| *COL11A1* | 0.07 | 0.80 | 11.43 | 3.35E-05 |  | *B3GNT7* | 5.84 | | 0.27 | | 0.05 | 3.35E-05 |  |
| *NKD1* | 0.30 | 3.31 | 11.03 | 3.35E-05 |  | *MT1H* | 1.53 | | 0.08 | | 0.05 | 3.21E-03 |  |
| *KRT7* | 0.02 | 0.25 | 10.89 | 9.90E-03 |  | *GCG* | 0.18 | | 0.01 | | 0.05 | 1.02E-04 |  |
| *KRT23* | 0.14 | 1.49 | 10.77 | 3.73E-05 |  | *BMP3* | 0.46 | | 0.02 | | 0.05 | 3.35E-05 |  |
| *CNTD2* | 0.02 | 0.23 | 10.59 | 1.64E-03 |  | *MT1M* | 1.57 | | 0.08 | | 0.05 | 9.33E-05 |  |
| *CYP2W1* | 0.24 | 2.52 | 10.53 | 1.18E-03 |  | *PRIMA1* | 0.25 | | 0.01 | | 0.05 | 3.35E-05 |  |
| *WNT2* | 0.02 | 0.23 | 10.11 | 3.39E-05 |  | *ADH1B* | 0.66 | | 0.04 | | 0.06 | 3.35E-05 |  |
| *TBX20* | 0.01 | 0.08 | 10.04 | 3.66E-02 |  | *B4GALNT2* | 1.32 | | 0.08 | | 0.06 | 3.35E-05 |  |
| *SPP1* | 0.21 | 2.02 | 9.62 | 3.35E-05 |  | *SFRP1* | 0.48 | | 0.03 | | 0.06 | 3.45E-05 |  |
| *KLK6* | 0.08 | 0.79 | 9.51 | 2.33E-04 |  | *VSTM2A* | 0.15 | | 0.01 | | 0.06 | 3.35E-05 |  |
| *C6orf223* | 0.11 | 1.01 | 9.31 | 3.35E-05 |  | *SCARA5* | 0.91 | | 0.06 | | 0.06 | 3.35E-05 |  |
| *KRT80* | 0.09 | 0.84 | 9.08 | 3.35E-05 |  | *CHP2* | 3.23 | | 0.22 | | 0.07 | 3.35E-05 |  |
| *MAGEB17* | 0.18 | 1.65 | 8.98 | 9.62E-03 |  | *BEST2* | 0.59 | | 0.04 | | 0.07 | 5.15E-05 |  |
| *SHISA2* | 0.03 | 0.24 | 8.95 | 5.71E-05 |  | *SLC30A10* | 0.30 | | 0.02 | | 0.07 | 3.35E-05 |  |
| *FOXQ1* | 0.25 | 2.23 | 8.92 | 3.38E-05 |  | *SLC6A19* | 0.26 | | 0.02 | | 0.07 | 3.44E-05 |  |
| *PIWIL1* | 0.04 | 0.38 | 8.72 | 4.89E-02 |  | *SLC26A3* | 15.55 | | 1.14 | | 0.07 | 3.35E-05 |  |
| *GNG4* | 0.05 | 0.44 | 8.65 | 2.28E-03 |  | *SCNN1G* | 0.19 | | 0.02 | | 0.08 | 2.83E-03 |  |
| *AZGP1* | 0.09 | 0.72 | 8.48 | 3.52E-05 |  | *BRINP3* | 0.26 | | 0.02 | | 0.08 | 3.53E-05 |  |
| *SERPINB5* | 0.18 | 1.52 | 8.41 | 4.20E-05 |  | *SST* | 0.22 | | 0.02 | | 0.08 | 2.36E-02 |  |
| *ZIC2* | 0.03 | 0.25 | 8.33 | 4.18E-03 |  | *AMPD1* | 0.28 | | 0.02 | | 0.08 | 4.87E-05 |  |
| *INHBA* | 0.14 | 1.20 | 8.28 | 3.35E-05 |  | *BEST4* | 0.44 | | 0.04 | | 0.08 | 1.29E-04 |  |
| *GRIN2D* | 0.09 | 0.71 | 8.11 | 3.35E-05 |  | *RPL10L* | 0.36 | | 0.03 | | 0.08 | 3.38E-04 |  |
| *STRA6* | 0.03 | 0.21 | 8.11 | 4.65E-04 |  | *SLC4A4* | 2.87 | | 0.24 | | 0.09 | 3.35E-05 |  |
| *SLC35D3* | 0.02 | 0.19 | 7.99 | 2.46E-03 |  | *CEACAM7* | 18.57 | | 1.65 | | 0.09 | 3.35E-05 |  |
| *BBOX1* | 0.04 | 0.31 | 7.98 | 5.18E-05 |  | *CLCA4* | 2.73 | | 0.25 | | 0.09 | 3.35E-05 |  |
| *TNS4* | 0.43 | 3.41 | 7.89 | 3.35E-05 |  | *GPR15* | 1.06 | | 0.10 | | 0.09 | 3.35E-05 |  |
| *TACSTD2* | 0.11 | 0.88 | 7.85 | 3.59E-05 |  | *GLP2R* | 0.22 | | 0.02 | | 0.09 | 3.35E-05 |  |
| *CBX2* | 0.05 | 0.41 | 7.80 | 3.35E-05 |  | *IGJ* | 71.14 | | 6.73 | | 0.09 | 3.35E-05 |  |
| *HS6ST2* | 0.04 | 0.35 | 7.74 | 3.69E-05 |  | *SI* | 1.50 | | 0.15 | | 0.10 | 3.35E-05 |  |
| *ASCL2* | 0.37 | 2.86 | 7.70 | 3.38E-05 |  | *C21orf88* | 0.34 | | 0.03 | | 0.10 | 2.92E-03 |  |
| *MSLN* | 0.19 | 1.45 | 7.68 | 9.85E-04 |  | *ENAM* | 0.90 | | 0.09 | | 0.10 | 3.35E-05 |  |
| *PRSS33* | 0.04 | 0.33 | 7.67 | 4.68E-03 |  | *HSD3B2* | 0.11 | | 0.01 | | 0.10 | 1.29E-04 |  |
| *PKP1* | 0.02 | 0.19 | 7.66 | 5.17E-04 |  | *TRPM6* | 1.10 | | 0.12 | | 0.11 | 3.35E-05 |  |
| *OTX1* | 0.01 | 0.07 | 7.66 | 2.35E-03 |  | *CNTN3* | 0.45 | | 0.05 | | 0.11 | 3.35E-05 |  |
| *GAD1* | 0.01 | 0.10 | 7.66 | 7.24E-04 |  | *LGALS9C* | 0.16 | | 0.02 | | 0.11 | 3.81E-03 |  |
| *C2CD4A* | 0.07 | 0.55 | 7.64 | 3.35E-05 |  | *KCNJ16* | 0.06 | | 0.01 | | 0.11 | 1.05E-02 |  |
| *CPNE7* | 0.17 | 1.26 | 7.52 | 3.47E-05 |  | *NOVA1* | 0.19 | | 0.02 | | 0.12 | 3.37E-05 |  |
| *KAL1* | 0.03 | 0.26 | 7.40 | 3.35E-05 |  | *MT1G* | 6.09 | | 0.72 | | 0.12 | 5.76E-05 |  |
| *NKD2* | 0.11 | 0.81 | 7.39 | 3.44E-05 |  | *CA1* | 2.16 | | 0.26 | | 0.12 | 3.35E-05 |  |
| *ZIC5* | 0.02 | 0.13 | 7.32 | 4.83E-03 |  | *ANKRD62* | 0.05 | | 0.01 | | 0.12 | 8.59E-04 |  |
| *C1QTNF8* | 0.01 | 0.10 | 7.30 | 4.91E-03 |  | *CHGA* | 1.75 | | 0.21 | | 0.12 | 3.35E-05 |  |
| *SFRP4* | 0.10 | 0.72 | 7.29 | 3.35E-05 |  | *ANPEP* | 5.46 | | 0.67 | | 0.12 | 3.35E-05 |  |
| *TESC* | 0.17 | 1.19 | 7.19 | 1.64E-04 |  | *USP2* | 0.28 | | 0.03 | | 0.12 | 3.35E-05 |  |
| *TNFSF9* | 0.07 | 0.50 | 7.19 | 9.04E-03 |  | *PKIB* | 0.92 | | 0.12 | | 0.13 | 3.43E-05 |  |
| *CA9* | 0.16 | 1.13 | 7.17 | 6.13E-03 |  | *NXPE1* | 7.72 | | 0.98 | | 0.13 | 3.35E-05 |  |
| *ESM1* | 0.03 | 0.22 | 7.15 | 6.62E-05 |  | *NXPE4* | 3.81 | | 0.49 | | 0.13 | 3.35E-05 |  |
| *CDH3* | 0.11 | 0.79 | 7.15 | 3.35E-05 |  | *PADI2* | 4.69 | | 0.61 | | 0.13 | 3.35E-05 |  |
| *KRT17* | 0.08 | 0.55 | 7.14 | 9.70E-03 |  | *ABCA8* | 0.57 | | 0.07 | | 0.13 | 3.35E-05 |  |
| *SULT2B1* | 0.06 | 0.39 | 6.97 | 1.09E-04 |  | *ATP13A4* | 0.47 | | 0.06 | | 0.13 | 3.35E-05 |  |
| *FLJ22184* | 0.04 | 0.25 | 6.89 | 5.46E-05 |  | *ALPI* | 0.18 | | 0.02 | | 0.13 | 8.12E-04 |  |
| *MSX2* | 0.05 | 0.36 | 6.87 | 3.14E-03 |  | *CNTFR* | 0.21 | | 0.03 | | 0.13 | 1.03E-02 |  |
| *SSTR5* | 0.02 | 0.14 | 6.86 | 1.50E-02 |  | *SLC26A2* | 12.88 | | 1.72 | | 0.13 | 3.35E-05 |  |
| *CLDN1* | 0.32 | 2.16 | 6.66 | 3.35E-05 |  | *ASPA* | 0.17 | | 0.02 | | 0.14 | 3.35E-05 |  |
| *SULT1C3* | 0.16 | 1.06 | 6.65 | 3.42E-05 |  | *CADM3* | 0.23 | | 0.03 | | 0.14 | 3.84E-05 |  |
| *TUBB3* | 0.07 | 0.44 | 6.65 | 3.35E-05 |  | *CLCA1* | 11.45 | | 1.57 | | 0.14 | 3.35E-05 |  |
| *ETV4* | 0.31 | 2.07 | 6.60 | 3.35E-05 |  | *TTLL6* | 0.59 | | 0.08 | | 0.14 | 3.35E-05 |  |
| *PDX1* | 0.06 | 0.37 | 6.60 | 7.44E-03 |  | *ITLN1* | 3.28 | | 0.45 | | 0.14 | 3.35E-05 |  |
| *SIM2* | 0.07 | 0.47 | 6.58 | 3.65E-05 |  | *SLC37A2* | 1.37 | | 0.19 | | 0.14 | 2.92E-04 |  |
| *KRT6B* | 0.03 | 0.20 | 6.56 | 4.17E-03 |  | *PCK1* | 3.49 | | 0.48 | | 0.14 | 3.35E-05 |  |
| *ONECUT2* | 0.02 | 0.15 | 6.55 | 8.93E-05 |  | *SCN9A* | 0.42 | | 0.06 | | 0.14 | 3.35E-05 |  |
| *UBD* | 0.48 | 3.10 | 6.49 | 1.21E-04 |  | *CCL13* | 0.55 | | 0.08 | | 0.14 | 2.82E-04 |  |
| *TRIM29* | 0.12 | 0.76 | 6.48 | 3.39E-05 |  | *MT1F* | 2.97 | | 0.42 | | 0.14 | 5.49E-04 |  |
| *KIF26B* | 0.07 | 0.47 | 6.46 | 3.35E-05 |  | *SPATA31E1* | 0.07 | | 0.01 | | 0.14 | 1.62E-04 |  |
| *MYOM3* | 0.08 | 0.49 | 6.36 | 9.69E-05 |  | *BTNL8* | 0.60 | | 0.09 | | 0.14 | 3.35E-05 |  |
| *HTR1D* | 0.05 | 0.34 | 6.27 | 9.49E-05 |  | *MAL* | 0.08 | | 0.01 | | 0.14 | 3.87E-02 |  |
| *MMP11* | 0.22 | 1.35 | 6.20 | 3.35E-05 |  | *RP11-386G21.2* | 0.11 | | 0.02 | | 0.14 | 2.20E-02 |  |
| *PPAPDC1A* | 0.02 | 0.10 | 6.19 | 4.48E-02 |  | *B3GALT1* | 0.38 | | 0.05 | | 0.14 | 3.40E-05 |  |
| *SLC4A11* | 0.05 | 0.30 | 6.10 | 4.74E-04 |  | *SLCO4C1* | 0.08 | | 0.01 | | 0.15 | 3.53E-05 |  |
| *MYADML2* | 0.01 | 0.07 | 6.09 | 4.72E-02 |  | *DNASE1L3* | 0.19 | | 0.03 | | 0.15 | 4.23E-05 |  |
| *STC2* | 0.07 | 0.42 | 6.08 | 3.42E-05 |  | *CLEC9A* | 0.05 | | 0.01 | | 0.15 | 1.54E-02 |  |
| *ADAMTS12* | 0.09 | 0.53 | 6.08 | 3.37E-05 |  | *BTNL3* | 1.39 | | 0.21 | | 0.15 | 3.35E-05 |  |
| *DKK2* | 0.02 | 0.11 | 6.00 | 4.45E-04 |  | *SCN7A* | 0.19 | | 0.03 | | 0.15 | 4.55E-05 |  |
| *TRIB3* | 0.16 | 0.96 | 5.99 | 3.35E-05 |  | *HTR4* | 0.20 | | 0.03 | | 0.15 | 3.35E-05 |  |
| *MDFI* | 0.08 | 0.46 | 5.87 | 3.39E-05 |  | *SLC17A4* | 1.72 | | 0.27 | | 0.15 | 3.35E-05 |  |
| *GYLTL1B* | 0.14 | 0.80 | 5.84 | 7.13E-05 |  | *TMEM72* | 0.29 | | 0.05 | | 0.15 | 2.71E-04 |  |
| *PACSIN3* | 0.07 | 0.39 | 5.83 | 1.24E-03 |  | *PKHD1L1* | 0.09 | | 0.01 | | 0.16 | 7.28E-04 |  |
| *SALL4* | 0.07 | 0.40 | 5.81 | 3.50E-05 |  | *NPY1R* | 0.25 | | 0.04 | | 0.16 | 3.38E-05 |  |
| *OLR1* | 0.08 | 0.44 | 5.80 | 3.35E-05 |  | *ZBTB16* | 0.42 | | 0.07 | | 0.16 | 3.35E-05 |  |
| *CXCL8* | 0.26 | 1.49 | 5.74 | 4.13E-05 |  | *ASPG* | 0.59 | | 0.09 | | 0.16 | 3.36E-05 |  |
| *COMP* | 0.06 | 0.32 | 5.69 | 1.76E-03 |  | *FAM135B* | 0.05 | | 0.01 | | 0.16 | 5.69E-05 |  |
| *THBS2* | 0.42 | 2.41 | 5.68 | 3.35E-05 |  | *ABCB11* | 0.09 | | 0.01 | | 0.16 | 8.03E-05 |  |
| *SP5* | 0.05 | 0.31 | 5.65 | 8.32E-05 |  | *SLC51B* | 0.63 | | 0.10 | | 0.16 | 2.75E-04 |  |
| *RAD51AP1* | 0.20 | 1.11 | 5.64 | 4.92E-05 |  | *HEPACAM2* | 1.05 | | 0.17 | | 0.16 | 3.35E-05 |  |
| *PLK5* | 0.03 | 0.19 | 5.59 | 4.66E-03 |  | *FAM107A* | 0.26 | | 0.04 | | 0.16 | 3.60E-05 |  |
| *KLK7* | 0.04 | 0.25 | 5.56 | 6.65E-03 |  | *ADTRP* | 1.62 | | 0.26 | | 0.16 | 3.35E-05 |  |
| *BMP7* | 0.11 | 0.61 | 5.50 | 4.71E-04 |  | *VSIG2* | 3.59 | | 0.59 | | 0.16 | 3.35E-05 |  |
| *VWA2* | 0.23 | 1.27 | 5.49 | 3.39E-05 |  | *BAI3* | 0.11 | | 0.02 | | 0.16 | 8.42E-05 |  |
| *GAL* | 0.06 | 0.34 | 5.43 | 2.70E-02 |  | *C11orf86* | 0.20 | | 0.03 | | 0.16 | 1.92E-03 |  |
| *PAX9* | 0.02 | 0.09 | 5.42 | 1.20E-02 |  | *PAPPA2* | 0.09 | | 0.01 | | 0.16 | 3.65E-05 |  |
| *ATG9B* | 0.06 | 0.30 | 5.35 | 1.41E-03 |  | *CTD-2545M3.6* | 0.80 | | 0.13 | | 0.17 | 3.35E-05 |  |
| *SCD* | 1.55 | 8.22 | 5.29 | 3.35E-05 |  | *GPR98* | 0.12 | | 0.02 | | 0.17 | 3.38E-05 |  |
| *EVX1* | 0.03 | 0.14 | 5.24 | 2.94E-02 |  | *MUSK* | 0.21 | | 0.04 | | 0.17 | 3.36E-05 |  |
| *EPHX4* | 0.03 | 0.15 | 5.21 | 1.39E-02 |  | *GFRA3* | 0.08 | | 0.01 | | 0.17 | 3.26E-03 |  |
| *SPTBN2* | 0.17 | 0.87 | 5.18 | 3.35E-05 |  | *CWH43* | 0.34 | | 0.06 | | 0.17 | 3.38E-05 |  |
| *EREG* | 0.36 | 1.86 | 5.15 | 6.42E-04 |  | *ZNF536* | 0.17 | | 0.03 | | 0.18 | 3.37E-05 |  |
| *PROX1* | 0.30 | 1.56 | 5.14 | 3.35E-05 |  | *LGALS9B* | 0.10 | | 0.02 | | 0.18 | 2.10E-03 |  |
| *ANKRD13B* | 0.14 | 0.69 | 5.12 | 3.55E-05 |  | *LPHN3* | 0.44 | | 0.08 | | 0.18 | 3.35E-05 |  |
| *ITGBL1* | 0.04 | 0.20 | 5.06 | 3.80E-05 |  | *XKR4* | 0.08 | | 0.01 | | 0.18 | 2.25E-03 |  |
| *ZNF695* | 0.05 | 0.24 | 5.03 | 4.07E-04 |  | *SEMA6D* | 0.90 | | 0.16 | | 0.18 | 3.35E-05 |  |
| *CCDC74A* | 0.01 | 0.06 | 5.03 | 4.05E-02 |  | *TNXB* | 0.58 | | 0.10 | | 0.18 | 3.35E-05 |  |
| *IL17RD* | 0.07 | 0.33 | 4.95 | 5.24E-05 |  | *ATP1A2* | 0.14 | | 0.03 | | 0.18 | 4.72E-05 |  |
| *MMP7* | 0.18 | 0.90 | 4.95 | 3.39E-05 |  | *UGT2B15* | 0.20 | | 0.04 | | 0.18 | 5.72E-04 |  |
| *MMP1* | 0.23 | 1.13 | 4.94 | 2.45E-04 |  | *PRKG2* | 0.14 | | 0.03 | | 0.19 | 1.95E-03 |  |
| *FAP* | 0.07 | 0.36 | 4.91 | 3.35E-05 |  | *CCL19* | 0.79 | | 0.15 | | 0.19 | 1.40E-02 |  |
| *NEK2* | 0.10 | 0.48 | 4.88 | 3.39E-05 |  | *BMP5* | 0.42 | | 0.08 | | 0.19 | 3.35E-05 |  |
| *TFAP2A* | 0.04 | 0.19 | 4.86 | 1.02E-02 |  | *CPM* | 1.07 | | 0.20 | | 0.19 | 3.35E-05 |  |
| *SRMS* | 0.08 | 0.39 | 4.84 | 3.99E-02 |  | *C7* | 0.54 | | 0.10 | | 0.19 | 3.35E-05 |  |
| *SH3TC2* | 0.06 | 0.27 | 4.81 | 3.39E-05 |  | *PLP1* | 0.13 | | 0.03 | | 0.19 | 3.85E-05 |  |
| *C16orf59* | 0.05 | 0.25 | 4.80 | 1.34E-02 |  | *NXPE2* | 1.38 | | 0.26 | | 0.19 | 3.35E-05 |  |
| *MEX3A* | 0.06 | 0.27 | 4.79 | 1.23E-02 |  | *ADAMDEC1* | 3.41 | | 0.66 | | 0.19 | 3.35E-05 |  |
| *SULF1* | 0.77 | 3.68 | 4.78 | 3.35E-05 |  | *CHST5* | 1.40 | | 0.27 | | 0.19 | 3.38E-05 |  |
| *PSAT1* | 0.26 | 1.23 | 4.77 | 1.10E-04 |  | *EDN3* | 1.05 | | 0.20 | | 0.19 | 3.35E-05 |  |
| *ANLN* | 0.35 | 1.67 | 4.77 | 3.35E-05 |  | *NR1H4* | 0.23 | | 0.04 | | 0.19 | 1.07E-03 |  |
| *AXIN2* | 1.28 | 6.08 | 4.77 | 3.38E-05 |  | *MMRN1* | 0.15 | | 0.03 | | 0.19 | 7.44E-04 |  |
| *NTSR1* | 0.05 | 0.23 | 4.74 | 7.51E-03 |  | *KHDRBS2* | 0.08 | | 0.02 | | 0.19 | 6.15E-03 |  |
| *PCSK9* | 0.17 | 0.79 | 4.72 | 3.51E-05 |  | *GCNT2* | 0.21 | | 0.04 | | 0.20 | 3.35E-05 |  |
| *DSCC1* | 0.11 | 0.51 | 4.71 | 3.38E-05 |  | *COL19A1* | 0.10 | | 0.02 | | 0.20 | 3.39E-05 |  |
| *FAM150A* | 0.04 | 0.19 | 4.69 | 2.62E-02 |  | *TSPAN7* | 0.90 | | 0.18 | | 0.20 | 3.35E-05 |  |
| *TGFBI* | 1.28 | 5.98 | 4.69 | 3.35E-05 |  | *ST6GALNAC6* | 3.91 | | 0.77 | | 0.20 | 3.35E-05 |  |
| *CCL24* | 0.39 | 1.82 | 4.68 | 3.72E-02 |  | *UGT1A7* | 1.05 | | 0.21 | | 0.20 | 3.35E-05 |  |
| *DUSP4* | 0.18 | 0.84 | 4.68 | 5.56E-04 |  | *SCUBE1* | 0.20 | | 0.04 | | 0.20 | 3.35E-05 |  |
| *DIO2* | 0.07 | 0.31 | 4.68 | 5.27E-05 |  | *MS4A8* | 0.55 | | 0.11 | | 0.20 | 4.07E-05 |  |
| *NOX4* | 0.02 | 0.08 | 4.67 | 9.17E-05 |  | *PLAC8* | 2.80 | | 0.56 | | 0.20 | 3.35E-05 |  |
| *PNPLA3* | 0.06 | 0.28 | 4.67 | 1.22E-03 |  | *KCNA3* | 0.63 | | 0.13 | | 0.20 | 3.35E-05 |  |
| *TDGF1* | 0.08 | 0.37 | 4.67 | 6.91E-04 |  | *LDHD* | 0.86 | | 0.17 | | 0.20 | 3.42E-05 |  |
| *NTM* | 0.06 | 0.30 | 4.63 | 3.47E-05 |  | *CCDC152* | 7.34 | | 1.48 | | 0.20 | 3.35E-05 |  |
| *POU5F1B* | 0.07 | 0.31 | 4.62 | 2.66E-03 |  | *DHRS9* | 1.39 | | 0.28 | | 0.20 | 3.35E-05 |  |
| *KIAA1549L* | 0.03 | 0.12 | 4.57 | 3.58E-05 |  | *BEND4* | 0.07 | | 0.01 | | 0.20 | 3.38E-05 |  |
| *AJUBA* | 0.14 | 0.66 | 4.56 | 3.35E-05 |  | *ADAM29* | 0.03 | | 0.01 | | 0.20 | 1.96E-03 |  |
| *LGR5* | 0.56 | 2.55 | 4.54 | 5.14E-05 |  | *CCL8* | 0.25 | | 0.05 | | 0.21 | 5.72E-03 |  |
| *HIST3H2A* | 0.07 | 0.31 | 4.53 | 1.54E-02 |  | *TLL1* | 0.11 | | 0.02 | | 0.21 | 5.48E-04 |  |
| *UBE2C* | 0.42 | 1.88 | 4.49 | 3.38E-05 |  | *DISP2* | 0.60 | | 0.12 | | 0.21 | 3.35E-05 |  |
| *PHLDA1* | 1.09 | 4.90 | 4.48 | 3.35E-05 |  | *SEPP1* | 5.86 | | 1.22 | | 0.21 | 3.35E-05 |  |
| *SLC6A6* | 0.61 | 2.75 | 4.48 | 3.35E-05 |  | *HAPLN1* | 0.10 | | 0.02 | | 0.21 | 6.74E-05 |  |
| *VSNL1* | 0.15 | 0.68 | 4.48 | 3.70E-04 |  | *ABCC8* | 0.04 | | 0.01 | | 0.21 | 5.50E-03 |  |
| *CLDN2* | 0.43 | 1.91 | 4.47 | 3.56E-05 |  | *WSCD1* | 0.36 | | 0.08 | | 0.21 | 3.42E-05 |  |
| *CACNG8* | 0.03 | 0.12 | 4.45 | 4.99E-03 |  | *PDK4* | 1.19 | | 0.25 | | 0.21 | 3.35E-05 |  |
| *SOX11* | 0.01 | 0.04 | 4.45 | 5.06E-04 |  | *KRT24* | 0.08 | | 0.02 | | 0.21 | 3.76E-02 |  |
| *NEBL* | 0.31 | 1.38 | 4.45 | 3.35E-05 |  | *SLC4A10* | 0.09 | | 0.02 | | 0.21 | 2.22E-04 |  |
| *COL12A1* | 0.90 | 3.99 | 4.42 | 3.35E-05 |  | *SNTG2* | 0.05 | | 0.01 | | 0.21 | 1.71E-02 |  |
| *ECT2* | 0.57 | 2.52 | 4.42 | 3.35E-05 |  | *TNFRSF13B* | 0.24 | | 0.05 | | 0.21 | 6.29E-04 |  |
| *TPX2* | 0.57 | 2.52 | 4.41 | 3.35E-05 |  | *ASXL3* | 0.23 | | 0.05 | | 0.21 | 3.35E-05 |  |
| *ASIC1* | 0.08 | 0.36 | 4.40 | 3.26E-04 |  | *CA12* | 4.49 | | 0.96 | | 0.21 | 3.35E-05 |  |
| *CEMIP* | 0.51 | 2.21 | 4.37 | 3.35E-05 |  | *C14orf64* | 0.18 | | 0.04 | | 0.21 | 3.37E-05 |  |
| *FUT1* | 0.05 | 0.22 | 4.34 | 1.16E-04 |  | *UGT1A9* | 1.20 | | 0.26 | | 0.21 | 3.35E-05 |  |
| *MYEOV* | 0.22 | 0.96 | 4.32 | 1.53E-04 |  | *PDE7B* | 0.22 | | 0.05 | | 0.22 | 3.35E-05 |  |
| *FOSL1* | 0.16 | 0.71 | 4.31 | 1.05E-03 |  | *NRXN1* | 0.12 | | 0.03 | | 0.22 | 3.35E-05 |  |
| *PFDN4* | 0.16 | 0.69 | 4.30 | 3.51E-05 |  | *MOGAT2* | 1.49 | | 0.32 | | 0.22 | 3.35E-05 |  |
| *CYP19A1* | 0.05 | 0.20 | 4.25 | 6.26E-05 |  | *ABCG2* | 0.24 | | 0.05 | | 0.22 | 4.19E-04 |  |
| *CXCL10* | 0.31 | 1.31 | 4.25 | 1.05E-04 |  | *ENPP6* | 0.04 | | 0.01 | | 0.22 | 1.01E-02 |  |
| *SAPCD2* | 0.34 | 1.46 | 4.24 | 3.35E-05 |  | *TPSB2* | 0.33 | | 0.07 | | 0.22 | 2.04E-03 |  |
| *PRSS22* | 0.21 | 0.88 | 4.24 | 7.46E-05 |  | *CLEC10A* | 0.29 | | 0.06 | | 0.22 | 7.88E-05 |  |
| *TOP2A* | 0.79 | 3.35 | 4.24 | 3.35E-05 |  | *SYT10* | 0.04 | | 0.01 | | 0.22 | 1.64E-02 |  |
| *HILPDA* | 0.26 | 1.09 | 4.23 | 5.74E-05 |  | *LRRC7* | 0.05 | | 0.01 | | 0.22 | 3.89E-04 |  |
| *CTHRC1* | 0.25 | 1.06 | 4.23 | 3.36E-05 |  | *MUC12* | 11.46 | | 2.51 | | 0.22 | 3.35E-05 |  |
| *SLC2A1* | 0.88 | 3.71 | 4.22 | 3.36E-05 |  | *KIAA0125* | 0.55 | | 0.12 | | 0.22 | 3.35E-05 |  |
| *CDCA5* | 0.15 | 0.65 | 4.19 | 3.35E-05 |  | *BLK* | 0.19 | | 0.04 | | 0.22 | 3.35E-05 |  |
| *BGN* | 1.28 | 5.36 | 4.19 | 3.35E-05 |  | *CBLN2* | 0.05 | | 0.01 | | 0.22 | 3.69E-02 |  |
| *SLCO4A1* | 0.38 | 1.60 | 4.18 | 6.50E-05 |  | *FCER2* | 0.12 | | 0.03 | | 0.22 | 3.94E-02 |  |
| *PERP* | 2.76 | 11.52 | 4.18 | 3.35E-05 |  | *PCSK5* | 1.24 | | 0.28 | | 0.22 | 3.35E-05 |  |
| *SNTB1* | 0.34 | 1.44 | 4.17 | 3.37E-05 |  | *HDC* | 0.29 | | 0.07 | | 0.22 | 6.28E-05 |  |
| *CENPW* | 0.16 | 0.66 | 4.17 | 2.67E-04 |  | *MS4A1* | 0.85 | | 0.19 | | 0.22 | 3.35E-05 |  |
| *IL11* | 0.04 | 0.18 | 4.17 | 1.46E-03 |  | *PIGR* | 165.61 | | 37.11 | | 0.22 | 3.35E-05 |  |
| *CCNB1* | 0.27 | 1.11 | 4.15 | 4.66E-05 |  | *LYVE1* | 0.24 | | 0.05 | | 0.22 | 4.97E-04 |  |
| *MACC1* | 0.64 | 2.66 | 4.14 | 3.35E-05 |  | *HHLA2* | 2.14 | | 0.48 | | 0.23 | 3.35E-05 |  |
| *GRHL3* | 0.04 | 0.15 | 4.14 | 5.50E-03 |  | *UGT1A1* | 1.08 | | 0.24 | | 0.23 | 3.35E-05 |  |
| *GDPD5* | 0.13 | 0.53 | 4.13 | 4.71E-04 |  | *PDE9A* | 1.35 | | 0.30 | | 0.23 | 3.35E-05 |  |
| *ANO1* | 0.30 | 1.23 | 4.11 | 2.45E-04 |  | *B3GALT5* | 2.06 | | 0.47 | | 0.23 | 3.35E-05 |  |
| *UCN2* | 0.04 | 0.18 | 4.08 | 1.05E-02 |  | *TMEM82* | 0.12 | | 0.03 | | 0.23 | 8.73E-03 |  |
| *CEP55* | 0.23 | 0.94 | 4.08 | 3.57E-05 |  | *CA7* | 0.73 | | 0.17 | | 0.23 | 6.78E-05 |  |
| *RNF43* | 2.03 | 8.28 | 4.07 | 3.35E-05 |  | *LIFR* | 0.35 | | 0.08 | | 0.23 | 3.38E-05 |  |
| *NPM3* | 0.26 | 1.04 | 4.06 | 5.98E-03 |  | *FCRL2* | 0.42 | | 0.10 | | 0.23 | 3.35E-05 |  |
| *EFNA3* | 0.21 | 0.84 | 4.05 | 2.97E-02 |  | *CLEC4F* | 0.10 | | 0.02 | | 0.23 | 2.97E-02 |  |
| *SP6* | 0.09 | 0.36 | 4.05 | 4.40E-04 |  | *UGT1A5* | 1.11 | | 0.26 | | 0.23 | 3.35E-05 |  |
| *MET* | 0.89 | 3.60 | 4.05 | 3.35E-05 |  | *GREM2* | 0.40 | | 0.09 | | 0.23 | 3.45E-05 |  |
| *MAD2L1* | 0.18 | 0.74 | 4.04 | 3.51E-03 |  | *TRPV3* | 0.24 | | 0.06 | | 0.23 | 3.36E-05 |  |
| *PAFAH1B3* | 0.21 | 0.85 | 4.02 | 1.21E-03 |  | *UGT1A10* | 1.09 | | 0.25 | | 0.23 | 3.35E-05 |  |
| *MLXIPL* | 0.30 | 1.20 | 4.01 | 3.69E-05 |  | *TNFRSF17* | 0.37 | | 0.09 | | 0.23 | 3.15E-03 |  |
| *STRIP2* | 0.07 | 0.27 | 4.00 | 4.65E-05 |  | *GFRA2* | 0.07 | | 0.02 | | 0.24 | 1.18E-04 |  |
| *CENPF* | 0.50 | 2.00 | 3.99 | 3.35E-05 |  | *B3GNT6* | 1.50 | | 0.35 | | 0.24 | 3.35E-05 |  |
| *SULT1C2* | 0.28 | 1.12 | 3.98 | 3.35E-05 |  | *TMEM100* | 0.11 | | 0.02 | | 0.24 | 8.84E-03 |  |
| *SLC22A3* | 0.17 | 0.70 | 3.98 | 3.39E-05 |  | *HMGCS2* | 5.37 | | 1.28 | | 0.24 | 3.42E-05 |  |
| *COL1A1* | 6.51 | 25.79 | 3.96 | 3.35E-05 |  | *ACKR1* | 0.18 | | 0.04 | | 0.24 | 9.40E-04 |  |
| *WISP1* | 0.10 | 0.39 | 3.96 | 2.72E-04 |  | *FGF9* | 0.14 | | 0.03 | | 0.24 | 5.21E-05 |  |
| *PPM1H* | 0.30 | 1.19 | 3.95 | 3.35E-05 |  | *GNG7* | 0.33 | | 0.08 | | 0.24 | 3.35E-05 |  |
| *REEP6* | 0.11 | 0.43 | 3.95 | 5.01E-03 |  | *IRF4* | 1.05 | | 0.25 | | 0.24 | 3.35E-05 |  |
| *SLC7A11* | 0.20 | 0.80 | 3.93 | 1.50E-04 |  | *MAMDC2* | 0.46 | | 0.11 | | 0.24 | 3.35E-05 |  |
| *JAG2* | 0.21 | 0.80 | 3.92 | 4.37E-05 |  | *FCRL5* | 0.64 | | 0.16 | | 0.24 | 3.35E-05 |  |
| *TRIP13* | 0.14 | 0.56 | 3.92 | 3.36E-05 |  | *MUC4* | 7.94 | | 1.92 | | 0.24 | 3.35E-05 |  |
| *ARNTL2* | 0.24 | 0.93 | 3.91 | 3.35E-05 |  | *FCRL1* | 0.32 | | 0.08 | | 0.24 | 3.35E-05 |  |
| *HES4* | 0.17 | 0.66 | 3.90 | 3.56E-02 |  | *RIC3* | 0.12 | | 0.03 | | 0.24 | 9.88E-05 |  |
| *GPT2* | 0.29 | 1.12 | 3.90 | 3.57E-05 |  | *FHL1* | 1.22 | | 0.30 | | 0.24 | 3.35E-05 |  |
| *MCM10* | 0.09 | 0.34 | 3.90 | 5.52E-05 |  | *IL1RAPL1* | 0.06 | | 0.01 | | 0.24 | 4.82E-02 |  |
| *CLCN4* | 0.05 | 0.18 | 3.88 | 8.13E-03 |  | *ALOX12B* | 0.22 | | 0.05 | | 0.24 | 2.02E-03 |  |
| *HIST1H3J* | 0.56 | 2.16 | 3.88 | 7.09E-05 |  | *MYH15* | 0.27 | | 0.07 | | 0.24 | 3.35E-05 |  |
| *CEACAM6* | 8.42 | 32.70 | 3.88 | 3.36E-05 |  | *ABI3BP* | 0.67 | | 0.16 | | 0.24 | 3.35E-05 |  |
| *IL1A* | 0.09 | 0.37 | 3.88 | 1.10E-03 |  | *TCF21* | 0.41 | | 0.10 | | 0.24 | 3.58E-05 |  |
| *HIST3H2BB* | 0.11 | 0.43 | 3.88 | 3.67E-02 |  | *UGT1A3* | 1.07 | | 0.26 | | 0.24 | 3.35E-05 |  |
| *KPNA2* | 0.54 | 2.10 | 3.88 | 3.35E-05 |  | *CNR1* | 0.11 | | 0.03 | | 0.25 | 3.38E-05 |  |
| *SLC13A3* | 0.03 | 0.13 | 3.87 | 4.84E-02 |  | *P2RY14* | 0.40 | | 0.10 | | 0.25 | 3.95E-05 |  |
| *MMP3* | 0.19 | 0.72 | 3.87 | 1.47E-02 |  | *ISX* | 0.97 | | 0.24 | | 0.25 | 3.35E-05 |  |
| *GTF2IRD1* | 0.39 | 1.50 | 3.86 | 3.35E-05 |  | *NR3C2* | 2.67 | | 0.66 | | 0.25 | 3.35E-05 |  |
| *ALPK3* | 0.14 | 0.53 | 3.85 | 4.06E-02 |  | *CDH19* | 0.13 | | 0.03 | | 0.25 | 2.04E-04 |  |
| *S100P* | 0.62 | 2.41 | 3.85 | 2.11E-03 |  | *UGT1A6* | 0.74 | | 0.18 | | 0.25 | 3.35E-05 |  |
| *QPCT* | 0.08 | 0.31 | 3.85 | 1.51E-03 |  | *EDNRB* | 0.52 | | 0.13 | | 0.25 | 3.35E-05 |  |
| *IFI6* | 1.68 | 6.44 | 3.84 | 3.37E-05 |  | *NRGN* | 0.88 | | 0.22 | | 0.25 | 3.35E-05 |  |
| *EDAR* | 0.17 | 0.64 | 3.84 | 2.23E-02 |  | *SEMA6A* | 2.11 | | 0.52 | | 0.25 | 3.35E-05 |  |
| *SKA3* | 0.19 | 0.73 | 3.84 | 6.62E-05 |  | *UGT1A4* | 1.08 | | 0.27 | | 0.25 | 3.35E-05 |  |
| *EFNA3* | 0.25 | 0.97 | 3.83 | 2.95E-04 |  | *HSPB6* | 0.97 | | 0.24 | | 0.25 | 3.58E-05 |  |
| *SORD* | 0.25 | 0.94 | 3.83 | 3.36E-05 |  | *KIAA2022* | 0.04 | | 0.01 | | 0.25 | 2.71E-03 |  |
| *CCDC113* | 0.07 | 0.27 | 3.83 | 3.48E-05 |  | *POU2AF1* | 0.82 | | 0.21 | | 0.25 | 3.35E-05 |  |
| *RRM2* | 0.41 | 1.56 | 3.83 | 3.35E-05 |  | *UGT1A8* | 0.72 | | 0.18 | | 0.25 | 3.35E-05 |  |
| *EIF5A2* | 0.06 | 0.22 | 3.81 | 3.64E-05 |  | *SPATA22* | 0.06 | | 0.01 | | 0.25 | 6.39E-03 |  |
| *FAM216B* | 0.04 | 0.17 | 3.81 | 1.46E-03 |  | *UGT2A3* | 0.82 | | 0.21 | | 0.25 | 3.35E-05 |  |
| *LRP8* | 0.16 | 0.59 | 3.80 | 1.25E-04 |  | *TRPC7* | 0.11 | | 0.03 | | 0.25 | 1.25E-03 |  |
| *GGH* | 0.30 | 1.15 | 3.79 | 3.82E-05 |  | *CCL28* | 2.11 | | 0.53 | | 0.25 | 3.35E-05 |  |
| *CKS2* | 0.43 | 1.63 | 3.79 | 1.60E-03 |  | *P2RX1* | 0.61 | | 0.15 | | 0.25 | 3.35E-05 |  |
| *ZAK* | 0.36 | 1.34 | 3.77 | 3.35E-05 |  | *SEC14L5* | 0.06 | | 0.01 | | 0.25 | 8.71E-03 |  |
| *HSPD1* | 1.86 | 6.97 | 3.76 | 3.35E-05 |  | *HPSE2* | 0.12 | | 0.03 | | 0.26 | 1.72E-03 |  |
| *IFITM1* | 1.49 | 5.60 | 3.75 | 3.70E-04 |  | *NKX2-3* | 0.33 | | 0.08 | | 0.26 | 1.49E-04 |  |
| *UBE2T* | 0.17 | 0.65 | 3.75 | 1.82E-03 |  | *BCAS1* | 1.69 | | 0.43 | | 0.26 | 3.35E-05 |  |
| *RPL22L1* | 0.19 | 0.69 | 3.72 | 1.19E-04 |  | *RSPO2* | 0.06 | | 0.02 | | 0.26 | 5.67E-03 |  |
| *DNAH2* | 0.03 | 0.10 | 3.71 | 2.49E-02 |  | *CNR2* | 0.10 | | 0.03 | | 0.26 | 1.19E-03 |  |
| *HSPH1* | 1.18 | 4.37 | 3.71 | 3.35E-05 |  | *GRIA4* | 0.04 | | 0.01 | | 0.26 | 4.05E-05 |  |
| *TEAD4* | 0.20 | 0.74 | 3.69 | 3.68E-05 |  | *NR5A2* | 0.87 | | 0.23 | | 0.26 | 3.35E-05 |  |
| *ASB9* | 0.06 | 0.22 | 3.69 | 3.69E-03 |  | *ITM2C* | 17.88 | | 4.65 | | 0.26 | 3.35E-05 |  |
| *WDR72* | 0.07 | 0.25 | 3.69 | 5.51E-03 |  | *TBX10* | 0.34 | | 0.09 | | 0.26 | 3.46E-03 |  |
| *DGAT2* | 0.07 | 0.25 | 3.69 | 7.34E-05 |  | *COX6B2* | 0.70 | | 0.18 | | 0.26 | 6.63E-05 |  |
| *KIAA1549* | 0.15 | 0.55 | 3.69 | 3.35E-05 |  | *CDHR5* | 6.31 | | 1.66 | | 0.26 | 3.35E-05 |  |
| *LRRC15* | 0.07 | 0.27 | 3.68 | 3.76E-04 |  | *CD163L1* | 0.56 | | 0.15 | | 0.27 | 3.35E-05 |  |
| *APLN* | 0.03 | 0.12 | 3.68 | 4.15E-02 |  | *SPINK4* | 3.31 | | 0.88 | | 0.27 | 1.60E-03 |  |
| *APCDD1* | 0.48 | 1.77 | 3.68 | 3.58E-02 |  | *TMEM37* | 0.71 | | 0.19 | | 0.27 | 3.36E-05 |  |
| *IL20RA* | 0.17 | 0.63 | 3.68 | 2.23E-04 |  | *HSD11B2* | 7.59 | | 2.02 | | 0.27 | 3.35E-05 |  |
| *PCDHB9* | 0.04 | 0.15 | 3.67 | 1.48E-03 |  | *SEMA3D* | 0.07 | | 0.02 | | 0.27 | 7.09E-05 |  |
| *PHGDH* | 0.09 | 0.33 | 3.67 | 2.28E-02 |  | *HSD17B2* | 0.90 | | 0.24 | | 0.27 | 4.86E-05 |  |
| *HES6* | 0.32 | 1.18 | 3.67 | 3.49E-04 |  | *ANO5* | 0.20 | | 0.06 | | 0.27 | 3.37E-05 |  |
| *SLC2A12* | 0.11 | 0.41 | 3.66 | 7.02E-04 |  | *CD79A* | 1.02 | | 0.28 | | 0.27 | 4.20E-05 |  |
| *MTHFD1L* | 0.35 | 1.30 | 3.66 | 3.35E-05 |  | *FAM189A2* | 0.12 | | 0.03 | | 0.27 | 4.48E-03 |  |
| *CSE1L* | 0.88 | 3.24 | 3.66 | 3.35E-05 |  | *LHFPL3* | 0.52 | | 0.14 | | 0.27 | 4.43E-05 |  |
| *CCNF* | 0.25 | 0.91 | 3.66 | 4.54E-05 |  | *CSMD1* | 0.04 | | 0.01 | | 0.27 | 3.55E-05 |  |
| *CPZ* | 0.08 | 0.28 | 3.66 | 3.66E-03 |  | *PDE6A* | 0.14 | | 0.04 | | 0.27 | 3.38E-05 |  |
| *CKAP2L* | 0.12 | 0.46 | 3.66 | 3.69E-05 |  | *SLITRK2* | 0.02 | | 0.01 | | 0.27 | 2.53E-02 |  |
| *PALD1* | 0.35 | 1.27 | 3.65 | 5.60E-05 |  | *CNTN4* | 0.15 | | 0.04 | | 0.27 | 5.32E-05 |  |
| *PMEPA1* | 0.96 | 3.49 | 3.65 | 4.30E-05 |  | *UNC5D* | 0.03 | | 0.01 | | 0.27 | 1.49E-02 |  |
| *BIRC5* | 0.29 | 1.05 | 3.65 | 6.47E-05 |  | *TENM1* | 0.08 | | 0.02 | | 0.27 | 3.70E-05 |  |
| *MYBL2* | 0.57 | 2.07 | 3.63 | 3.37E-05 |  | *KIAA1683* | 0.63 | | 0.17 | | 0.27 | 3.35E-05 |  |
| *CDK1* | 0.26 | 0.94 | 3.63 | 5.78E-05 |  | *CAPN13* | 0.56 | | 0.15 | | 0.27 | 3.58E-05 |  |
| *GINS1* | 0.20 | 0.74 | 3.61 | 4.76E-05 |  | *NRG1* | 0.20 | | 0.06 | | 0.28 | 1.96E-04 |  |
| *E2F7* | 0.09 | 0.32 | 3.61 | 4.68E-04 |  | *TMEM220* | 0.54 | | 0.15 | | 0.28 | 3.35E-05 |  |
| *ULBP3* | 0.03 | 0.13 | 3.61 | 4.98E-02 |  | *PLCXD3* | 0.04 | | 0.01 | | 0.28 | 5.15E-03 |  |
| *SPOCK1* | 0.10 | 0.36 | 3.60 | 3.37E-05 |  | *METTL7A* | 3.16 | | 0.88 | | 0.28 | 3.35E-05 |  |
| *PAQR4* | 0.43 | 1.53 | 3.58 | 3.48E-05 |  | *HHIP* | 0.38 | | 0.11 | | 0.28 | 3.35E-05 |  |
| *MYC* | 1.33 | 4.75 | 3.58 | 3.72E-05 |  | *LILRB5* | 0.22 | | 0.06 | | 0.28 | 3.69E-05 |  |
| *TMEM97* | 0.56 | 2.02 | 3.58 | 3.35E-05 |  | *EYA2* | 0.35 | | 0.10 | | 0.28 | 3.59E-04 |  |
| *CKAP2* | 0.41 | 1.47 | 3.57 | 3.37E-05 |  | *PLCE1* | 2.32 | | 0.65 | | 0.28 | 3.35E-05 |  |
| *S100A11* | 3.91 | 13.94 | 3.56 | 3.35E-05 |  | *DPF3* | 0.22 | | 0.06 | | 0.28 | 3.35E-05 |  |
| *SPTBN5* | 0.11 | 0.38 | 3.56 | 2.05E-02 |  | *STOX2* | 0.17 | | 0.05 | | 0.28 | 2.37E-04 |  |
| *SIX4* | 0.02 | 0.06 | 3.56 | 5.31E-03 |  | *CDKL2* | 0.10 | | 0.03 | | 0.28 | 2.67E-02 |  |
| *BUB1* | 0.23 | 0.82 | 3.56 | 3.35E-05 |  | *ADAMTS1* | 1.17 | | 0.33 | | 0.29 | 3.35E-05 |  |
| *KIF14* | 0.16 | 0.56 | 3.55 | 5.41E-05 |  | *VAT1L* | 0.17 | | 0.05 | | 0.29 | 9.84E-05 |  |
| *STMN1* | 0.32 | 1.13 | 3.55 | 3.35E-05 |  | *TRIM40* | 0.29 | | 0.08 | | 0.29 | 7.46E-04 |  |
| *TRNP1* | 0.12 | 0.42 | 3.55 | 1.97E-04 |  | *PTGDS* | 0.70 | | 0.20 | | 0.29 | 1.04E-03 |  |
| *LGR6* | 0.13 | 0.46 | 3.55 | 2.83E-03 |  | *ANO7* | 1.88 | | 0.54 | | 0.29 | 3.35E-05 |  |
| *BMP4* | 0.68 | 2.40 | 3.55 | 1.57E-03 |  | *GP9* | 0.41 | | 0.12 | | 0.29 | 1.15E-04 |  |
| *KIAA0895* | 0.07 | 0.26 | 3.54 | 1.58E-04 |  | *LY9* | 0.17 | | 0.05 | | 0.29 | 3.48E-05 |  |
| *FIBIN* | 0.11 | 0.41 | 3.54 | 8.89E-04 |  | *CR2* | 0.28 | | 0.08 | | 0.29 | 6.87E-05 |  |
| *MORC4* | 0.27 | 0.95 | 3.54 | 3.57E-05 |  | *ADAMTSL1* | 0.24 | | 0.07 | | 0.29 | 3.35E-05 |  |
| *PKMYT1* | 0.35 | 1.22 | 3.54 | 4.44E-05 |  | *ITGA8* | 0.32 | | 0.09 | | 0.29 | 3.35E-05 |  |
| *PTK7* | 0.28 | 0.98 | 3.53 | 3.35E-05 |  | *CASP5* | 0.59 | | 0.17 | | 0.29 | 1.24E-03 |  |
| *SOX4* | 1.25 | 4.40 | 3.53 | 3.35E-05 |  | *FCRL4* | 0.05 | | 0.01 | | 0.29 | 4.15E-02 |  |
| *CBX8* | 0.08 | 0.28 | 3.53 | 3.82E-04 |  | *C1orf200* | 0.16 | | 0.05 | | 0.29 | 5.70E-03 |  |
| *LDHB* | 1.17 | 4.11 | 3.53 | 2.05E-04 |  | *CLDN23* | 0.86 | | 0.25 | | 0.29 | 3.48E-05 |  |
| *RGS16* | 0.20 | 0.69 | 3.52 | 5.19E-05 |  | *CD36* | 0.19 | | 0.06 | | 0.29 | 1.37E-04 |  |
| *PUS7* | 0.31 | 1.08 | 3.52 | 3.38E-05 |  | *FAM177B* | 0.44 | | 0.13 | | 0.29 | 3.42E-05 |  |
| *SLC7A5* | 0.68 | 2.40 | 3.52 | 4.80E-05 |  | *IL6R* | 1.03 | | 0.30 | | 0.29 | 3.35E-05 |  |
| *PRR11* | 0.34 | 1.18 | 3.51 | 4.44E-05 |  | *OSTN* | 0.04 | | 0.01 | | 0.29 | 1.67E-02 |  |
| *ARHGAP11A* | 0.28 | 0.97 | 3.51 | 3.35E-05 |  | *CYP2C19* | 0.35 | | 0.10 | | 0.29 | 1.59E-04 |  |
| *CELSR3* | 0.14 | 0.48 | 3.51 | 3.57E-05 |  | *MOBP* | 0.04 | | 0.01 | | 0.29 | 3.05E-04 |  |
| *C10orf113* | 0.28 | 0.99 | 3.51 | 1.13E-03 |  | *DIRAS1* | 0.05 | | 0.02 | | 0.29 | 1.16E-03 |  |
| *RHPN1* | 0.31 | 1.07 | 3.50 | 5.62E-05 |  | *AKR1B10* | 1.01 | | 0.30 | | 0.29 | 4.74E-04 |  |
| *SLC11A1* | 0.09 | 0.30 | 3.50 | 6.69E-05 |  | *GPT* | 1.05 | | 0.31 | | 0.29 | 3.47E-05 |  |
| *RNASEH2A* | 0.25 | 0.88 | 3.49 | 3.48E-05 |  | *NPHS2* | 0.19 | | 0.05 | | 0.29 | 1.73E-03 |  |
| *RFC3* | 0.25 | 0.88 | 3.49 | 6.76E-05 |  | *PZP* | 0.24 | | 0.07 | | 0.29 | 4.53E-04 |  |
| *FAM83D* | 0.24 | 0.85 | 3.49 | 3.45E-05 |  | *GRIK3* | 0.05 | | 0.02 | | 0.29 | 3.35E-03 |  |
| *DTL* | 0.19 | 0.66 | 3.49 | 3.36E-05 |  | *COL4A6* | 0.11 | | 0.03 | | 0.29 | 1.81E-03 |  |
| *SLC6A20* | 0.09 | 0.31 | 3.49 | 5.65E-04 |  | *PPARGC1A* | 0.78 | | 0.23 | | 0.30 | 3.35E-05 |  |
| *MZT1* | 0.24 | 0.82 | 3.49 | 5.27E-05 |  | *CACNA1G* | 0.04 | | 0.01 | | 0.30 | 5.34E-03 |  |
| *CDC6* | 0.24 | 0.83 | 3.48 | 3.55E-05 |  | *EMR1* | 0.09 | | 0.03 | | 0.30 | 1.60E-02 |  |
| *MME* | 0.10 | 0.35 | 3.48 | 8.98E-03 |  | *LPAR1* | 0.71 | | 0.21 | | 0.30 | 3.35E-05 |  |
| *UBE2S* | 0.14 | 0.50 | 3.48 | 3.67E-03 |  | *DES* | 4.78 | | 1.42 | | 0.30 | 1.35E-03 |  |
| *FSCN1* | 0.22 | 0.78 | 3.48 | 1.32E-03 |  | *FAM46C* | 2.83 | | 0.84 | | 0.30 | 3.35E-05 |  |
| *TWIST1* | 0.03 | 0.12 | 3.48 | 7.09E-03 |  | *SLAMF7* | 0.83 | | 0.25 | | 0.30 | 3.35E-05 |  |
| *GPR115* | 0.07 | 0.23 | 3.48 | 8.78E-03 |  | *PLA2G10* | 0.27 | | 0.08 | | 0.30 | 4.76E-05 |  |
| *EXO1* | 0.12 | 0.40 | 3.47 | 1.95E-04 |  | *ADAM33* | 0.58 | | 0.17 | | 0.30 | 3.95E-05 |  |
| *GDF15* | 1.01 | 3.51 | 3.47 | 6.97E-05 |  | *SLC13A2* | 0.30 | | 0.09 | | 0.30 | 1.17E-03 |  |
| *IQGAP3* | 0.55 | 1.92 | 3.47 | 3.35E-05 |  | *FCRL3* | 0.21 | | 0.06 | | 0.30 | 8.62E-04 |  |
| *MELK* | 0.29 | 1.02 | 3.46 | 1.48E-04 |  | *FER1L6* | 0.63 | | 0.19 | | 0.30 | 4.01E-05 |  |
| *EVA1A* | 0.06 | 0.21 | 3.46 | 8.54E-03 |  | *MT1X* | 2.10 | | 0.63 | | 0.30 | 2.15E-02 |  |
| *TNFRSF12A* | 0.71 | 2.44 | 3.45 | 3.75E-05 |  | *PDZD3* | 1.12 | | 0.34 | | 0.30 | 3.35E-05 |  |
| *COL8A1* | 0.18 | 0.61 | 3.45 | 3.35E-05 |  | *KLF4* | 4.43 | | 1.34 | | 0.30 | 3.35E-05 |  |
| *PTPRU* | 0.11 | 0.39 | 3.44 | 6.58E-04 |  | *FCRLA* | 0.09 | | 0.03 | | 0.30 | 4.63E-03 |  |
| *HKDC1* | 0.30 | 1.04 | 3.42 | 5.65E-04 |  | *NEGR1* | 0.22 | | 0.07 | | 0.30 | 3.42E-05 |  |
| *HIST1H2BJ* | 0.42 | 1.44 | 3.42 | 1.75E-03 |  | *PCSK2* | 0.03 | | 0.01 | | 0.30 | 5.27E-03 |  |
| *GPR78* | 0.05 | 0.17 | 3.42 | 4.52E-03 |  | *SULT1B1* | 2.53 | | 0.77 | | 0.30 | 3.35E-05 |  |
| *PLEKHN1* | 0.07 | 0.24 | 3.42 | 4.90E-04 |  | *TRIM9* | 0.07 | | 0.02 | | 0.30 | 1.39E-03 |  |
| *MFI2* | 0.26 | 0.88 | 3.41 | 8.20E-04 |  | *TIFAB* | 0.09 | | 0.03 | | 0.30 | 1.15E-03 |  |
| *CDKN3* | 0.22 | 0.74 | 3.41 | 1.74E-04 |  | *TAT* | 0.04 | | 0.01 | | 0.30 | 3.12E-03 |  |
| *HJURP* | 0.21 | 0.73 | 3.41 | 3.60E-04 |  | *AMN* | 2.87 | | 0.88 | | 0.30 | 3.35E-05 |  |
| *PMAIP1* | 0.12 | 0.41 | 3.40 | 1.25E-03 |  | *MZB1* | 1.61 | | 0.49 | | 0.30 | 3.35E-05 |  |
| *MAPK15* | 0.22 | 0.76 | 3.40 | 1.47E-02 |  | *MAPK10* | 0.20 | | 0.06 | | 0.31 | 3.36E-05 |  |
| *ADAMTS2* | 0.27 | 0.92 | 3.40 | 3.37E-05 |  | *ABCA10* | 0.31 | | 0.10 | | 0.31 | 3.38E-05 |  |
| *NME1* | 0.26 | 0.89 | 3.39 | 3.37E-05 |  | *JAM2* | 0.27 | | 0.08 | | 0.31 | 6.51E-05 |  |
| *TOMM34* | 0.45 | 1.52 | 3.39 | 3.47E-05 |  | *RBFOX1* | 0.09 | | 0.03 | | 0.31 | 3.82E-03 |  |
| *ITGA11* | 0.28 | 0.95 | 3.38 | 3.41E-05 |  | *TPSAB1* | 0.35 | | 0.11 | | 0.31 | 7.88E-05 |  |
| *HOXB8* | 0.36 | 1.21 | 3.37 | 6.98E-04 |  | *CCDC153* | 0.49 | | 0.15 | | 0.31 | 3.37E-05 |  |
| *TTK* | 0.17 | 0.57 | 3.37 | 5.65E-05 |  | *LRRC19* | 1.74 | | 0.54 | | 0.31 | 3.35E-05 |  |
| *KIF23* | 0.28 | 0.95 | 3.37 | 5.84E-05 |  | *ALK* | 0.05 | | 0.01 | | 0.31 | 5.85E-03 |  |
| *EPHB3* | 0.81 | 2.71 | 3.36 | 7.76E-04 |  | *TSC22D3* | 2.01 | | 0.62 | | 0.31 | 3.35E-05 |  |
| *SLC12A2* | 3.58 | 12.02 | 3.36 | 3.35E-05 |  | *RSPO1* | 0.04 | | 0.01 | | 0.31 | 3.93E-02 |  |
| *LY6E* | 0.50 | 1.68 | 3.35 | 7.90E-04 |  | *ACTG2* | 1.89 | | 0.59 | | 0.31 | 5.73E-05 |  |
| *GPSM2* | 0.69 | 2.32 | 3.35 | 3.39E-05 |  | *AK1* | 1.20 | | 0.37 | | 0.31 | 5.14E-05 |  |
| *CDCA7* | 0.87 | 2.91 | 3.34 | 3.35E-05 |  | *MS4A2* | 0.16 | | 0.05 | | 0.31 | 2.59E-02 |  |
| *DNMT3B* | 0.08 | 0.28 | 3.34 | 5.04E-05 |  | *AHCYL2* | 3.65 | | 1.14 | | 0.31 | 3.35E-05 |  |
| *CHAF1B* | 0.13 | 0.43 | 3.34 | 5.15E-04 |  | *FABP2* | 0.42 | | 0.13 | | 0.31 | 4.66E-05 |  |
| *OR51E1* | 0.03 | 0.12 | 3.34 | 8.47E-03 |  | *SYNPO2* | 1.42 | | 0.44 | | 0.31 | 3.36E-05 |  |
| *TIMP1* | 1.34 | 4.46 | 3.33 | 3.65E-05 |  | *RMDN2* | 0.43 | | 0.14 | | 0.31 | 3.45E-05 |  |
| *MCM2* | 0.23 | 0.77 | 3.32 | 3.42E-05 |  | *IL1RL1* | 0.33 | | 0.10 | | 0.31 | 3.35E-05 |  |
| *KIF2C* | 0.20 | 0.67 | 3.32 | 5.22E-05 |  | *NGFR* | 0.14 | | 0.04 | | 0.31 | 8.97E-03 |  |
| *HMGA1* | 2.12 | 7.02 | 3.31 | 3.35E-05 |  | *MT1E* | 2.42 | | 0.76 | | 0.31 | 1.91E-04 |  |
| *PLA2G16* | 0.13 | 0.44 | 3.30 | 3.68E-05 |  | *RNF112* | 0.15 | | 0.05 | | 0.31 | 3.68E-04 |  |
| *BUB1B* | 0.30 | 1.00 | 3.29 | 3.75E-05 |  | *GPR171* | 0.19 | | 0.06 | | 0.31 | 1.73E-02 |  |
| *PLEKHS1* | 0.25 | 0.83 | 3.29 | 6.83E-05 |  | *CYP2C9* | 0.17 | | 0.05 | | 0.31 | 2.27E-03 |  |
| *XRCC2* | 0.18 | 0.59 | 3.27 | 4.53E-05 |  | *AFF3* | 0.24 | | 0.08 | | 0.32 | 3.35E-05 |  |
| *PCNA* | 0.69 | 2.25 | 3.26 | 4.20E-05 |  | *NRG2* | 0.04 | | 0.01 | | 0.32 | 3.89E-02 |  |
| *PLS3* | 0.46 | 1.51 | 3.26 | 3.35E-05 |  | *GNAO1* | 0.16 | | 0.05 | | 0.32 | 1.47E-02 |  |
| *ATP11A* | 0.42 | 1.37 | 3.26 | 3.35E-05 |  | *C15orf48* | 5.25 | | 1.66 | | 0.32 | 3.36E-05 |  |
| *PSRC1* | 0.10 | 0.33 | 3.25 | 3.45E-04 |  | *IGSF10* | 0.10 | | 0.03 | | 0.32 | 3.89E-05 |  |
| *KLHL31* | 0.04 | 0.12 | 3.25 | 5.63E-05 |  | *ABCA9* | 0.20 | | 0.06 | | 0.32 | 1.01E-04 |  |
| *TMEM132A* | 0.16 | 0.53 | 3.25 | 3.38E-05 |  | *IGLL5* | 27.48 | | 8.75 | | 0.32 | 3.35E-05 |  |
| *TLX1* | 0.04 | 0.14 | 3.25 | 1.76E-03 |  | *RASGRP2* | 0.25 | | 0.08 | | 0.32 | 3.53E-05 |  |
| *KIF18A* | 0.11 | 0.35 | 3.25 | 4.41E-03 |  | *RNF152* | 0.88 | | 0.28 | | 0.32 | 3.35E-05 |  |
| *CXCL1* | 0.92 | 2.98 | 3.25 | 1.29E-04 |  | *ANK2* | 0.25 | | 0.08 | | 0.32 | 3.35E-05 |  |
| *RAD54B* | 0.14 | 0.45 | 3.24 | 1.77E-03 |  | *DCLK1* | 0.11 | | 0.03 | | 0.32 | 3.42E-05 |  |
| *ZNRF3* | 0.31 | 1.01 | 3.24 | 3.38E-05 |  | *KCNG3* | 0.15 | | 0.05 | | 0.32 | 9.59E-05 |  |
| *CDCA2* | 0.19 | 0.61 | 3.24 | 5.70E-04 |  | *PLA2G2A* | 10.16 | | 3.25 | | 0.32 | 3.35E-05 |  |
| *CCND1* | 1.62 | 5.25 | 3.24 | 3.35E-05 |  | *PBLD* | 1.52 | | 0.49 | | 0.32 | 3.35E-05 |  |
| *FAM222A* | 0.10 | 0.32 | 3.23 | 1.75E-02 |  | *CHL1* | 0.17 | | 0.05 | | 0.32 | 5.29E-05 |  |
| *PTTG1* | 0.18 | 0.60 | 3.23 | 3.00E-02 |  | *CYSLTR1* | 0.10 | | 0.03 | | 0.32 | 1.49E-03 |  |
| *KIF4A* | 0.19 | 0.61 | 3.22 | 3.35E-05 |  | *ABCD2* | 0.08 | | 0.02 | | 0.32 | 7.39E-04 |  |
| *ASPM* | 0.36 | 1.16 | 3.22 | 3.35E-05 |  | *CD209* | 0.34 | | 0.11 | | 0.32 | 6.70E-05 |  |
| *AHNAK2* | 0.21 | 0.69 | 3.22 | 3.35E-05 |  | *LDB3* | 0.12 | | 0.04 | | 0.32 | 1.16E-04 |  |
| *MCM4* | 0.47 | 1.52 | 3.22 | 3.59E-05 |  | *VIPR1* | 1.99 | | 0.64 | | 0.32 | 3.35E-05 |  |
| *CENPI* | 0.16 | 0.51 | 3.22 | 5.09E-05 |  | *FOSB* | 4.10 | | 1.33 | | 0.32 | 3.68E-05 |  |
| *PRRX1* | 0.21 | 0.68 | 3.22 | 3.35E-05 |  | *NTRK3* | 0.04 | | 0.01 | | 0.32 | 2.53E-04 |  |
| *ISM1* | 0.07 | 0.23 | 3.21 | 1.31E-04 |  | *PNOC* | 0.13 | | 0.04 | | 0.32 | 1.75E-03 |  |
| *AHCY* | 1.15 | 3.69 | 3.21 | 3.35E-05 |  | *PDE4C* | 0.42 | | 0.14 | | 0.32 | 3.36E-05 |  |
| *CXCL3* | 0.56 | 1.78 | 3.20 | 3.29E-02 |  | *FAM129C* | 0.16 | | 0.05 | | 0.32 | 2.04E-04 |  |
| *PPIL1* | 0.32 | 1.03 | 3.20 | 1.95E-04 |  | *SLC25A34* | 0.25 | | 0.08 | | 0.32 | 4.34E-05 |  |
| *NPM1* | 1.34 | 4.28 | 3.20 | 3.35E-05 |  | *LINGO3* | 0.10 | | 0.03 | | 0.33 | 1.96E-02 |  |
| *NAP1L1* | 1.58 | 5.06 | 3.20 | 3.35E-05 |  | *PDGFD* | 0.32 | | 0.10 | | 0.33 | 3.79E-05 |  |
| *DKC1* | 0.68 | 2.17 | 3.20 | 3.35E-05 |  | *MPP2* | 0.10 | | 0.03 | | 0.33 | 5.97E-03 |  |
| *AURKA* | 0.27 | 0.86 | 3.18 | 5.64E-05 |  | *SLCO2A1* | 0.89 | | 0.29 | | 0.33 | 3.35E-05 |  |
| *FERMT1* | 1.97 | 6.26 | 3.18 | 3.35E-05 |  | *ADCY5* | 0.25 | | 0.08 | | 0.33 | 9.60E-05 |  |
| *MRPS23* | 0.40 | 1.27 | 3.18 | 8.24E-05 |  | *TMEM156* | 0.20 | | 0.07 | | 0.33 | 1.31E-04 |  |
| *FZD3* | 0.11 | 0.35 | 3.17 | 4.93E-05 |  | *ABCA6* | 0.53 | | 0.17 | | 0.33 | 3.36E-05 |  |
| *DIAPH3* | 0.19 | 0.60 | 3.17 | 1.38E-04 |  | *BANK1* | 0.30 | | 0.10 | | 0.33 | 3.70E-05 |  |
| *C6orf123* | 0.08 | 0.27 | 3.17 | 3.44E-02 |  | *ARHGAP20* | 0.14 | | 0.05 | | 0.33 | 4.52E-05 |  |
| *DLGAP5* | 0.26 | 0.83 | 3.17 | 3.36E-05 |  | *ADAM28* | 1.07 | | 0.35 | | 0.33 | 3.35E-05 |  |
| *MCM3* | 0.75 | 2.39 | 3.16 | 3.36E-05 |  | *KLHL6* | 0.63 | | 0.21 | | 0.33 | 3.35E-05 |  |
| *C19orf48* | 0.82 | 2.60 | 3.16 | 4.85E-05 |  | *CD22* | 0.59 | | 0.19 | | 0.33 | 3.35E-05 |  |
| *FGFRL1* | 0.46 | 1.46 | 3.15 | 4.47E-04 |  | *CPNE8* | 0.57 | | 0.19 | | 0.33 | 5.19E-03 |  |
| *CCNA2* | 0.42 | 1.31 | 3.15 | 3.55E-05 |  | *PKNOX2* | 0.08 | | 0.03 | | 0.33 | 1.91E-04 |  |
| *ADAM12* | 0.16 | 0.49 | 3.15 | 3.39E-05 |  | *CD40LG* | 0.12 | | 0.04 | | 0.33 | 7.31E-03 |  |
| *AC021218.2* | 0.52 | 1.63 | 3.15 | 3.46E-04 |  | *REP15* | 0.78 | | 0.26 | | 0.33 | 4.82E-05 |  |
| *LYZ* | 4.07 | 12.76 | 3.14 | 8.49E-04 |  | *SDCBP2* | 2.24 | | 0.75 | | 0.33 | 3.35E-05 |  |
| *IER5L* | 0.16 | 0.49 | 3.13 | 3.72E-05 |  | *KCNK10* | 0.07 | | 0.02 | | 0.33 | 1.35E-04 |  |
| *SHCBP1* | 0.12 | 0.37 | 3.13 | 4.73E-04 |  | *NLRP9* | 0.10 | | 0.03 | | 0.33 | 3.17E-03 |  |
| *KNSTRN* | 0.25 | 0.79 | 3.13 | 4.14E-05 |  | *PTGDR* | 0.94 | | 0.31 | | 0.33 | 3.38E-05 |  |
| *CENPE* | 0.26 | 0.80 | 3.12 | 6.74E-05 |  | *SCIN* | 0.52 | | 0.17 | | 0.33 | 3.41E-05 |  |
| *CDCA3* | 0.11 | 0.33 | 3.11 | 1.28E-03 |  | *LRRC18* | 0.19 | | 0.06 | | 0.33 | 1.32E-02 |  |
| *NUSAP1* | 0.60 | 1.88 | 3.11 | 3.40E-05 |  | *AGPAT9* | 0.44 | | 0.15 | | 0.33 | 1.76E-04 |  |
| *E2F1* | 0.25 | 0.77 | 3.11 | 1.49E-04 |  | *MFSD4* | 0.72 | | 0.24 | | 0.34 | 5.57E-05 |  |
| *FNDC1* | 0.17 | 0.52 | 3.11 | 3.35E-05 |  | *FAM189A1* | 0.23 | | 0.08 | | 0.34 | 3.38E-05 |  |
| *CENPA* | 0.12 | 0.37 | 3.11 | 4.31E-05 |  | *ITK* | 0.28 | | 0.09 | | 0.34 | 3.35E-05 |  |
| *EIF4EBP1* | 0.56 | 1.73 | 3.10 | 5.37E-05 |  | *PRKCB* | 0.37 | | 0.12 | | 0.34 | 3.35E-05 |  |
| *MEGF6* | 0.27 | 0.83 | 3.09 | 5.06E-05 |  | *TLR10* | 0.18 | | 0.06 | | 0.34 | 2.27E-04 |  |
| *C2orf70* | 0.09 | 0.29 | 3.09 | 3.14E-02 |  | *GIMAP7* | 0.32 | | 0.11 | | 0.34 | 1.21E-02 |  |
| *PMCH* | 0.23 | 0.72 | 3.09 | 3.38E-04 |  | *MEP1A* | 2.02 | | 0.68 | | 0.34 | 3.35E-05 |  |
| *LEF1* | 0.20 | 0.63 | 3.09 | 3.61E-05 |  | *SULT1A2* | 0.21 | | 0.07 | | 0.34 | 2.57E-04 |  |
| *CETN2* | 0.28 | 0.86 | 3.09 | 3.38E-05 |  | *SLIT3* | 0.32 | | 0.11 | | 0.34 | 3.48E-05 |  |
| *GLO1* | 1.14 | 3.52 | 3.09 | 3.35E-05 |  | *C8orf46* | 0.03 | | 0.01 | | 0.34 | 2.29E-02 |  |
| *DBNDD1* | 0.17 | 0.54 | 3.09 | 1.02E-04 |  | *WNT5B* | 0.23 | | 0.08 | | 0.34 | 3.03E-04 |  |
| *PBK* | 0.13 | 0.40 | 3.09 | 1.71E-03 |  | *SLC15A2* | 0.43 | | 0.15 | | 0.34 | 3.35E-05 |  |
| *GINS4* | 0.13 | 0.40 | 3.09 | 1.49E-04 |  | *BMP6* | 0.32 | | 0.11 | | 0.34 | 2.27E-04 |  |
| *PKM* | 4.21 | 12.99 | 3.08 | 3.35E-05 |  | *LCN6* | 0.07 | | 0.02 | | 0.34 | 1.37E-03 |  |
| *HIST1H2BL* | 0.64 | 1.96 | 3.08 | 1.98E-03 |  | *BHLHA15* | 0.19 | | 0.07 | | 0.34 | 2.23E-02 |  |
| *PODXL* | 0.74 | 2.26 | 3.07 | 3.35E-05 |  | *CCDC13* | 0.09 | | 0.03 | | 0.34 | 5.11E-03 |  |
| *SRCRB4D* | 0.08 | 0.23 | 3.07 | 2.91E-03 |  | *CD79B* | 0.23 | | 0.08 | | 0.34 | 6.70E-03 |  |
| *SPC24* | 0.20 | 0.61 | 3.07 | 2.47E-02 |  | *GPR174* | 0.31 | | 0.10 | | 0.34 | 9.18E-03 |  |
| *MKI67* | 1.11 | 3.41 | 3.07 | 3.35E-05 |  | *P2RY10* | 0.23 | | 0.08 | | 0.34 | 2.14E-03 |  |
| *FANCB* | 0.09 | 0.27 | 3.07 | 1.76E-04 |  | *MYOT* | 0.13 | | 0.05 | | 0.34 | 1.98E-03 |  |
| *ZNF469* | 0.12 | 0.37 | 3.07 | 3.35E-05 |  | *SCUBE2* | 0.16 | | 0.05 | | 0.34 | 1.43E-04 |  |
| *ANXA3* | 0.30 | 0.93 | 3.06 | 2.04E-04 |  | *NUGGC* | 0.18 | | 0.06 | | 0.34 | 7.56E-05 |  |
| *UTP14A* | 0.28 | 0.85 | 3.06 | 3.55E-05 |  | *CXCL12* | 0.71 | | 0.25 | | 0.34 | 3.39E-05 |  |
| *ALDH4A1* | 0.15 | 0.45 | 3.06 | 3.45E-04 |  | *RCSD1* | 0.62 | | 0.21 | | 0.34 | 3.35E-05 |  |
| *COL1A2* | 4.17 | 12.76 | 3.06 | 3.35E-05 |  | *PAX5* | 0.16 | | 0.06 | | 0.34 | 4.98E-04 |  |
| *LZTS3* | 0.23 | 0.69 | 3.05 | 3.36E-05 |  | *FAIM3* | 0.48 | | 0.16 | | 0.34 | 3.35E-05 |  |
| *HSP90AB1* | 5.23 | 15.96 | 3.05 | 3.35E-05 |  | *VIT* | 0.07 | | 0.03 | | 0.34 | 1.84E-02 |  |
| *ORC6* | 0.20 | 0.62 | 3.05 | 1.64E-04 |  | *HPGDS* | 0.15 | | 0.05 | | 0.35 | 1.56E-03 |  |
| *GGCT* | 0.62 | 1.87 | 3.05 | 3.35E-05 |  | *COL4A5* | 0.31 | | 0.11 | | 0.35 | 3.35E-05 |  |
| *CDK4* | 1.22 | 3.73 | 3.04 | 3.37E-05 |  | *MEI1* | 0.11 | | 0.04 | | 0.35 | 3.75E-05 |  |
| *AURKB* | 0.27 | 0.82 | 3.04 | 4.56E-03 |  | *ASB2* | 0.19 | | 0.07 | | 0.35 | 2.54E-03 |  |
| *FAM111B* | 0.27 | 0.83 | 3.04 | 3.82E-05 |  | *AC129492.6* | 0.21 | | 0.07 | | 0.35 | 4.46E-02 |  |
| *CXCL2* | 0.56 | 1.70 | 3.04 | 2.65E-02 |  | *DERL3* | 1.40 | | 0.49 | | 0.35 | 3.35E-05 |  |
| *NT5DC4* | 0.02 | 0.07 | 3.03 | 1.47E-02 |  | *TSPAN1* | 7.31 | | 2.54 | | 0.35 | 3.35E-05 |  |
| *SNRPF* | 0.26 | 0.77 | 3.02 | 3.81E-05 |  | *SIGLEC6* | 0.14 | | 0.05 | | 0.35 | 4.46E-02 |  |
| *LUM* | 1.29 | 3.89 | 3.01 | 3.81E-05 |  | *MYH11* | 8.17 | | 2.85 | | 0.35 | 3.36E-05 |  |
| *MGAT5* | 1.22 | 3.67 | 3.01 | 3.35E-05 |  | *TM4SF2* | 0.85 | | 0.30 | | 0.35 | 9.87E-03 |  |
| *CCT4* | 1.06 | 3.20 | 3.01 | 3.35E-05 |  | *CD69* | 0.39 | | 0.14 | | 0.35 | 2.35E-04 |  |
| *TICRR* | 0.19 | 0.57 | 3.01 | 3.58E-05 |  | *ACVRL1* | 1.91 | | 0.67 | | 0.35 | 3.35E-05 |  |
| *GPR180* | 0.31 | 0.92 | 3.01 | 3.45E-05 |  | *ALDOB* | 1.14 | | 0.40 | | 0.35 | 4.84E-04 |  |
| *PLK4* | 0.16 | 0.47 | 3.01 | 7.09E-05 |  | *ARRDC5* | 0.23 | | 0.08 | | 0.35 | 1.55E-02 |  |
| *ENC1* | 1.43 | 4.29 | 3.00 | 3.35E-05 |  | *PYHIN1* | 0.21 | | 0.07 | | 0.35 | 1.84E-03 |  |
| *PTPLAD1* | 1.00 | 3.00 | 3.00 | 3.35E-05 |  | *STMN2* | 0.23 | | 0.08 | | 0.35 | 1.19E-03 |  |
| *KIF11* | 0.35 | 1.05 | 2.99 | 5.64E-04 |  | *HGD* | 0.44 | | 0.15 | | 0.35 | 1.87E-04 |  |
| *MIF* | 1.24 | 3.71 | 2.99 | 3.58E-04 |  | *IGF1* | 0.35 | | 0.12 | | 0.35 | 3.35E-05 |  |
| *BRCA1* | 0.29 | 0.88 | 2.99 | 3.39E-05 |  | *BFSP2* | 0.09 | | 0.03 | | 0.35 | 2.46E-02 |  |
| *CLSPN* | 0.15 | 0.43 | 2.99 | 3.38E-05 |  | *ARHGAP44* | 1.19 | | 0.42 | | 0.35 | 3.35E-05 |  |
| *ZWILCH* | 0.29 | 0.85 | 2.98 | 1.31E-04 |  | *SYT2* | 0.46 | | 0.16 | | 0.35 | 3.35E-05 |  |
| *SNAI1* | 0.07 | 0.22 | 2.98 | 3.35E-03 |  | *CAMK2A* | 0.24 | | 0.08 | | 0.35 | 4.79E-05 |  |
| *LAMC2* | 0.72 | 2.14 | 2.98 | 3.42E-05 |  | *GLIPR2* | 0.74 | | 0.26 | | 0.35 | 3.43E-05 |  |
| *MFAP2* | 0.10 | 0.29 | 2.98 | 6.29E-03 |  | *C11orf21* | 0.10 | | 0.04 | | 0.35 | 6.20E-03 |  |
| *DEPDC1B* | 0.14 | 0.41 | 2.97 | 3.47E-04 |  | *HMCN2* | 0.57 | | 0.20 | | 0.36 | 3.38E-05 |  |
| *ENO1* | 3.27 | 9.73 | 2.97 | 3.40E-05 |  | *TESPA1* | 0.15 | | 0.05 | | 0.36 | 6.46E-04 |  |
| *TCFL5* | 0.21 | 0.62 | 2.97 | 1.61E-04 |  | *SORCS1* | 0.07 | | 0.02 | | 0.36 | 2.61E-04 |  |
| *SHMT2* | 0.60 | 1.79 | 2.97 | 4.17E-05 |  | *FAM65B* | 0.37 | | 0.13 | | 0.36 | 3.35E-05 |  |
| *MCM6* | 0.39 | 1.14 | 2.96 | 3.35E-05 |  | *ZBTB7C* | 1.01 | | 0.36 | | 0.36 | 3.40E-05 |  |
| *ITGA2* | 0.78 | 2.30 | 2.96 | 3.38E-05 |  | *FLT3* | 0.08 | | 0.03 | | 0.36 | 4.81E-04 |  |
| *CDC45* | 0.16 | 0.49 | 2.96 | 1.33E-03 |  | *DMD* | 0.64 | | 0.23 | | 0.36 | 3.35E-05 |  |
| *ZNF239* | 0.13 | 0.39 | 2.95 | 2.88E-02 |  | *PLCL2* | 0.70 | | 0.25 | | 0.36 | 3.35E-05 |  |
| *PAICS* | 1.37 | 4.05 | 2.94 | 3.35E-05 |  | *CPA3* | 0.61 | | 0.22 | | 0.36 | 6.00E-05 |  |
| *ISLR* | 0.30 | 0.89 | 2.94 | 3.71E-05 |  | *EPHA10* | 0.93 | | 0.33 | | 0.36 | 3.35E-05 |  |
| *HOPX* | 0.07 | 0.20 | 2.94 | 2.52E-04 |  | *SCN4B* | 0.25 | | 0.09 | | 0.36 | 3.35E-05 |  |
| *CDR2L* | 0.15 | 0.45 | 2.93 | 3.91E-05 |  | *GIMAP5* | 0.44 | | 0.16 | | 0.36 | 3.43E-05 |  |
| *NCAPG2* | 0.37 | 1.09 | 2.93 | 3.37E-05 |  | *RYR1* | 0.07 | | 0.03 | | 0.36 | 3.51E-05 |  |
| *KIF20A* | 0.16 | 0.46 | 2.93 | 9.61E-05 |  | *LAX1* | 0.37 | | 0.13 | | 0.36 | 3.43E-05 |  |
| *C12orf75* | 0.85 | 2.48 | 2.92 | 1.14E-04 |  | *CYP2C18* | 0.64 | | 0.23 | | 0.36 | 1.27E-03 |  |
| *HSP90AA1* | 3.89 | 11.35 | 2.92 | 3.35E-05 |  | *NUTM1* | 0.11 | | 0.04 | | 0.36 | 6.86E-04 |  |
| *ARID3A* | 0.48 | 1.39 | 2.92 | 6.30E-03 |  | *KCNT2* | 0.10 | | 0.03 | | 0.37 | 1.60E-02 |  |
| *TRAIP* | 0.10 | 0.28 | 2.92 | 4.94E-04 |  | *BTLA* | 0.14 | | 0.05 | | 0.37 | 2.86E-03 |  |
| *TROAP* | 0.26 | 0.76 | 2.91 | 4.86E-05 |  | *CNKSR2* | 0.04 | | 0.02 | | 0.37 | 4.82E-03 |  |
| *CDC25B* | 1.05 | 3.06 | 2.91 | 3.35E-05 |  | *CD1D* | 0.22 | | 0.08 | | 0.37 | 3.88E-03 |  |
| *LRRC6* | 0.14 | 0.40 | 2.91 | 1.54E-02 |  | *SLC16A9* | 0.54 | | 0.20 | | 0.37 | 3.45E-05 |  |
| *SLC39A10* | 0.37 | 1.09 | 2.91 | 3.35E-05 |  | *FRMD1* | 0.79 | | 0.29 | | 0.37 | 3.35E-05 |  |
| *RCN1* | 0.41 | 1.20 | 2.91 | 3.35E-05 |  | *C5orf20* | 0.08 | | 0.03 | | 0.37 | 1.16E-02 |  |
| *MDK* | 0.88 | 2.54 | 2.90 | 3.55E-03 |  | *ARL14* | 1.68 | | 0.62 | | 0.37 | 3.35E-05 |  |
| *ATAD2* | 0.41 | 1.21 | 2.90 | 3.35E-05 |  | *SLC9A9* | 0.32 | | 0.12 | | 0.37 | 3.69E-05 |  |
| *CGREF1* | 0.14 | 0.42 | 2.90 | 9.17E-05 |  | *SELENBP1* | 8.72 | | 3.21 | | 0.37 | 3.35E-05 |  |
| *CENPH* | 0.20 | 0.57 | 2.90 | 4.47E-04 |  | *SMIM5* | 1.01 | | 0.37 | | 0.37 | 3.35E-05 |  |
| *JPH1* | 0.33 | 0.95 | 2.90 | 3.42E-05 |  | *MFSD6L* | 0.17 | | 0.06 | | 0.37 | 6.69E-04 |  |
| *FASN* | 1.39 | 4.02 | 2.89 | 3.35E-05 |  | *HCRTR1* | 0.14 | | 0.05 | | 0.37 | 3.13E-03 |  |
| *TTC26* | 0.08 | 0.24 | 2.88 | 4.33E-05 |  | *ITM2A* | 0.19 | | 0.07 | | 0.37 | 1.10E-03 |  |
| *RAN* | 0.77 | 2.23 | 2.88 | 3.35E-05 |  | *HOXD1* | 0.16 | | 0.06 | | 0.37 | 1.37E-02 |  |
| *RACGAP1* | 0.42 | 1.21 | 2.88 | 3.48E-05 |  | *FAM159A* | 0.13 | | 0.05 | | 0.37 | 1.70E-02 |  |
| *TACC3* | 0.44 | 1.26 | 2.88 | 1.57E-04 |  | *ZNF831* | 0.13 | | 0.05 | | 0.37 | 3.99E-05 |  |
| *PYCR1* | 0.58 | 1.67 | 2.88 | 1.77E-04 |  | *NDST3* | 0.07 | | 0.03 | | 0.37 | 2.53E-04 |  |
| *NUF2* | 0.14 | 0.41 | 2.87 | 2.59E-04 |  | *BMP2* | 1.18 | | 0.44 | | 0.37 | 3.62E-05 |  |
| *SLC38A5* | 0.23 | 0.66 | 2.87 | 1.53E-03 |  | *ATP2A3* | 4.83 | | 1.79 | | 0.37 | 3.35E-05 |  |
| *PARPBP* | 0.18 | 0.51 | 2.87 | 3.48E-04 |  | *GSTM2* | 0.22 | | 0.08 | | 0.37 | 5.27E-05 |  |
| *ALDOC* | 0.20 | 0.58 | 2.87 | 1.55E-02 |  | *SLC6A7* | 0.80 | | 0.30 | | 0.37 | 3.35E-05 |  |
| *FKBP10* | 0.41 | 1.19 | 2.87 | 3.38E-05 |  | *FAM196B* | 0.35 | | 0.13 | | 0.37 | 4.10E-05 |  |
| *SNRPD2* | 0.97 | 2.79 | 2.86 | 1.42E-03 |  | *F13A1* | 0.63 | | 0.24 | | 0.37 | 1.39E-03 |  |
| *PDCD2L* | 0.16 | 0.46 | 2.86 | 4.86E-05 |  | *PNMA3* | 0.06 | | 0.02 | | 0.37 | 1.05E-02 |  |
| *PSMA7* | 1.35 | 3.85 | 2.86 | 3.35E-05 |  | *CLEC17A* | 0.15 | | 0.06 | | 0.37 | 3.48E-03 |  |
| *GRHL1* | 0.05 | 0.14 | 2.86 | 2.25E-02 |  | *AMICA1* | 0.50 | | 0.19 | | 0.38 | 3.35E-05 |  |
| *RPS21* | 2.95 | 8.42 | 2.86 | 3.69E-05 |  | *NLRP14* | 0.06 | | 0.02 | | 0.38 | 2.67E-02 |  |
| *CENPQ* | 0.11 | 0.30 | 2.85 | 6.16E-05 |  | *GHR* | 0.23 | | 0.09 | | 0.38 | 3.91E-05 |  |
| *UCHL3* | 0.69 | 1.97 | 2.85 | 2.53E-02 |  | *LCN10* | 0.06 | | 0.02 | | 0.38 | 2.47E-02 |  |
| *CYP2S1* | 0.99 | 2.82 | 2.85 | 3.53E-05 |  | *SLC9A2* | 1.41 | | 0.53 | | 0.38 | 3.35E-05 |  |
| *TBC1D16* | 0.60 | 1.70 | 2.85 | 3.57E-05 |  | *CES3* | 2.24 | | 0.85 | | 0.38 | 3.35E-05 |  |
| *MRPS12* | 0.29 | 0.81 | 2.85 | 4.07E-02 |  | *C1orf115* | 1.02 | | 0.38 | | 0.38 | 3.58E-05 |  |
| *SGOL2* | 0.13 | 0.36 | 2.85 | 6.22E-05 |  | *HRCT1* | 0.49 | | 0.19 | | 0.38 | 1.50E-03 |  |
| *GTF3A* | 1.04 | 2.97 | 2.85 | 3.35E-05 |  | *SMPDL3A* | 1.04 | | 0.40 | | 0.38 | 5.48E-05 |  |
| *FAM19A5* | 0.06 | 0.17 | 2.84 | 4.34E-02 |  | *FCN1* | 0.10 | | 0.04 | | 0.38 | 2.39E-02 |  |
| *PABPC1L* | 0.52 | 1.49 | 2.84 | 6.34E-04 |  | *CAPN9* | 1.01 | | 0.38 | | 0.38 | 4.53E-05 |  |
| *SLC29A1* | 0.44 | 1.24 | 2.84 | 3.80E-05 |  | *TPSG1* | 1.22 | | 0.47 | | 0.38 | 3.52E-05 |  |
| *PDCD5* | 1.28 | 3.64 | 2.84 | 3.35E-05 |  | *MYPN* | 0.03 | | 0.01 | | 0.38 | 1.41E-02 |  |
| *TGFB2* | 0.07 | 0.19 | 2.84 | 3.79E-05 |  | *SDPR* | 0.30 | | 0.12 | | 0.38 | 1.12E-04 |  |
| *IFT22* | 0.08 | 0.22 | 2.84 | 4.55E-04 |  | *CNN1* | 1.40 | | 0.54 | | 0.38 | 1.18E-03 |  |
| *LMTK3* | 0.09 | 0.25 | 2.84 | 2.63E-04 |  | *AOC1* | 2.89 | | 1.11 | | 0.38 | 3.35E-05 |  |
| *CITED4* | 0.10 | 0.29 | 2.84 | 1.13E-02 |  | *C16orf54* | 0.35 | | 0.13 | | 0.38 | 3.95E-05 |  |
| *CCT6A* | 1.32 | 3.75 | 2.83 | 3.35E-05 |  | *GIMAP1* | 0.15 | | 0.06 | | 0.38 | 3.46E-04 |  |
| *FAM169A* | 0.14 | 0.39 | 2.83 | 3.76E-03 |  | *MFAP4* | 2.76 | | 1.06 | | 0.39 | 3.49E-05 |  |
| *KIF18B* | 0.24 | 0.68 | 2.83 | 1.87E-03 |  | *RHOH* | 0.44 | | 0.17 | | 0.39 | 3.35E-05 |  |
| *CCT3* | 1.11 | 3.14 | 2.83 | 3.35E-05 |  | *ENTPD5* | 2.57 | | 0.99 | | 0.39 | 3.35E-05 |  |
| *IFITM3* | 3.21 | 9.08 | 2.83 | 3.60E-05 |  | *PDCD4* | 5.95 | | 2.29 | | 0.39 | 3.35E-05 |  |
| *KCTD14* | 0.27 | 0.76 | 2.82 | 6.90E-05 |  | *PDE2A* | 0.11 | | 0.04 | | 0.39 | 4.19E-05 |  |
| *MRGBP* | 0.55 | 1.55 | 2.82 | 1.81E-04 |  | *AXDND1* | 0.13 | | 0.05 | | 0.39 | 6.41E-04 |  |
| *CENPN* | 0.19 | 0.54 | 2.82 | 1.00E-03 |  | *CACNA1A* | 0.08 | | 0.03 | | 0.39 | 6.22E-05 |  |
| *KIF15* | 0.18 | 0.50 | 2.82 | 3.55E-05 |  | *SGSM1* | 0.17 | | 0.06 | | 0.39 | 4.02E-04 |  |
| *PPA1* | 1.81 | 5.11 | 2.82 | 3.35E-05 |  | *SYNM* | 0.72 | | 0.28 | | 0.39 | 2.79E-02 |  |
| *TTC9* | 0.10 | 0.29 | 2.81 | 3.30E-03 |  | *MIA2* | 0.18 | | 0.07 | | 0.39 | 3.37E-02 |  |
| *C17orf53* | 0.10 | 0.29 | 2.81 | 4.81E-03 |  | *PDE1C* | 0.10 | | 0.04 | | 0.39 | 9.21E-05 |  |
| *LYAR* | 0.22 | 0.63 | 2.81 | 3.58E-03 |  | *C7orf31* | 0.36 | | 0.14 | | 0.39 | 8.22E-04 |  |
| *ACAN* | 0.04 | 0.11 | 2.81 | 3.92E-04 |  | *TMEM171* | 1.06 | | 0.41 | | 0.39 | 1.43E-04 |  |
| *ME1* | 0.29 | 0.81 | 2.81 | 4.80E-05 |  | *C2orf72* | 1.21 | | 0.47 | | 0.39 | 3.35E-05 |  |
| *TYRO3* | 0.08 | 0.22 | 2.81 | 3.22E-03 |  | *SRI* | 3.41 | | 1.33 | | 0.39 | 3.35E-05 |  |
| *KIAA0101* | 0.21 | 0.60 | 2.81 | 3.19E-04 |  | *PHGR1* | 28.53 | | 11.13 | | 0.39 | 3.35E-05 |  |
| *CCDC85B* | 0.28 | 0.79 | 2.80 | 1.64E-02 |  | *KBTBD12* | 0.15 | | 0.06 | | 0.39 | 4.25E-05 |  |
| *IPO5* | 0.84 | 2.36 | 2.80 | 3.35E-05 |  | *PIM2* | 3.19 | | 1.25 | | 0.39 | 3.35E-05 |  |
| *POMP* | 1.04 | 2.92 | 2.80 | 1.28E-04 |  | *RIMS4* | 0.05 | | 0.02 | | 0.39 | 2.93E-02 |  |
| *NCAPH* | 0.18 | 0.51 | 2.80 | 8.28E-04 |  | *MGLL* | 3.44 | | 1.35 | | 0.39 | 3.35E-05 |  |
| *WDHD1* | 0.14 | 0.40 | 2.80 | 4.47E-05 |  | *ABCA5* | 1.70 | | 0.67 | | 0.39 | 3.35E-05 |  |
| *OLA1* | 0.50 | 1.40 | 2.80 | 3.42E-05 |  | *PNPLA7* | 0.47 | | 0.19 | | 0.39 | 3.35E-05 |  |
| *HMMR* | 0.21 | 0.57 | 2.80 | 2.48E-04 |  | *LRRN2* | 0.23 | | 0.09 | | 0.39 | 4.94E-05 |  |
| *XPOT* | 0.59 | 1.65 | 2.79 | 3.35E-05 |  | *RAB37* | 0.16 | | 0.06 | | 0.39 | 1.95E-03 |  |
| *DPM1* | 0.78 | 2.17 | 2.79 | 4.17E-04 |  | *PLCD1* | 0.63 | | 0.25 | | 0.39 | 3.35E-05 |  |
| *AMIGO2* | 0.21 | 0.57 | 2.79 | 3.69E-05 |  | *PARP15* | 0.43 | | 0.17 | | 0.39 | 3.38E-05 |  |
| *SERPINE2* | 0.22 | 0.61 | 2.79 | 1.66E-03 |  | *PTPRT* | 0.04 | | 0.01 | | 0.39 | 6.11E-03 |  |
| *NDUFAF2* | 0.25 | 0.71 | 2.79 | 1.42E-03 |  | *PRPH2* | 0.16 | | 0.06 | | 0.39 | 1.15E-02 |  |
| *PLAU* | 0.46 | 1.28 | 2.79 | 1.22E-04 |  | *TDRD10* | 0.10 | | 0.04 | | 0.40 | 2.11E-02 |  |
| *GINS2* | 0.21 | 0.58 | 2.79 | 1.22E-04 |  | *ACADS* | 1.28 | | 0.51 | | 0.40 | 7.25E-05 |  |
| *RP4-734P14.4* | 0.44 | 1.24 | 2.79 | 2.69E-03 |  | *FGFR2* | 0.67 | | 0.26 | | 0.40 | 4.13E-05 |  |
| *SQLE* | 0.65 | 1.80 | 2.78 | 3.35E-05 |  | *TP53INP2* | 2.32 | | 0.92 | | 0.40 | 3.35E-05 |  |
| *FEN1* | 0.26 | 0.71 | 2.78 | 1.63E-04 |  | *IL7R* | 1.01 | | 0.40 | | 0.40 | 3.35E-05 |  |
| *FOXM1* | 0.65 | 1.80 | 2.78 | 3.35E-05 |  | *SALL1* | 0.19 | | 0.08 | | 0.40 | 3.96E-04 |  |
| *CDT1* | 0.31 | 0.86 | 2.77 | 4.14E-03 |  | *NEU4* | 0.74 | | 0.30 | | 0.40 | 3.35E-05 |  |
| *GARS* | 1.02 | 2.83 | 2.77 | 3.35E-05 |  | *CFH* | 0.59 | | 0.24 | | 0.40 | 6.75E-05 |  |
| *NUP37* | 0.37 | 1.03 | 2.77 | 1.48E-04 |  | *CD48* | 0.42 | | 0.17 | | 0.40 | 1.45E-04 |  |
| *HSPA8* | 3.88 | 10.73 | 2.77 | 3.35E-05 |  | *IL10RA* | 1.21 | | 0.49 | | 0.40 | 3.35E-05 |  |
| *CCT2* | 1.50 | 4.15 | 2.76 | 3.35E-05 |  | *SLC24A4* | 0.05 | | 0.02 | | 0.40 | 7.65E-03 |  |
| *YEATS4* | 0.26 | 0.73 | 2.76 | 1.48E-03 |  | *DPEP2* | 0.26 | | 0.10 | | 0.40 | 7.64E-04 |  |
| *CYP4A22* | 0.02 | 0.05 | 2.76 | 4.63E-02 |  | *KRT20* | 8.55 | | 3.44 | | 0.40 | 3.35E-05 |  |
| *H2AFZ* | 0.55 | 1.51 | 2.75 | 1.24E-04 |  | *CHGB* | 0.07 | | 0.03 | | 0.40 | 1.92E-04 |  |
| *COL7A1* | 0.51 | 1.40 | 2.75 | 9.50E-05 |  | *ZC3H12C* | 0.67 | | 0.27 | | 0.40 | 3.35E-05 |  |
| *GTF2F2* | 0.47 | 1.29 | 2.75 | 6.50E-05 |  | *WDFY4* | 0.51 | | 0.21 | | 0.40 | 3.35E-05 |  |
| *SHROOM4* | 0.30 | 0.82 | 2.75 | 4.73E-05 |  | *RHBDL2* | 0.78 | | 0.32 | | 0.40 | 7.51E-05 |  |
| *OSM* | 0.08 | 0.21 | 2.75 | 2.48E-02 |  | *ENPP2* | 0.34 | | 0.14 | | 0.40 | 3.42E-05 |  |
| *FADS1* | 0.16 | 0.43 | 2.74 | 1.14E-03 |  | *EDIL3* | 0.86 | | 0.35 | | 0.40 | 3.35E-05 |  |
| *FN1* | 2.35 | 6.44 | 2.74 | 3.35E-05 |  | *ADAMTS8* | 0.07 | | 0.03 | | 0.40 | 8.18E-03 |  |
| *CHEK1* | 0.18 | 0.49 | 2.74 | 1.97E-04 |  | *TLR7* | 0.09 | | 0.03 | | 0.40 | 1.51E-04 |  |
| *ANO6* | 0.52 | 1.42 | 2.74 | 3.35E-05 |  | *BBIP1* | 2.18 | | 0.88 | | 0.40 | 3.35E-05 |  |
| *WDR62* | 0.13 | 0.35 | 2.74 | 7.41E-05 |  | *ACKR2* | 0.09 | | 0.04 | | 0.40 | 1.29E-03 |  |
| *TSPAN5* | 0.14 | 0.39 | 2.73 | 2.63E-04 |  | *MAP4K1* | 0.53 | | 0.22 | | 0.41 | 6.51E-04 |  |
| *ZWINT* | 0.31 | 0.86 | 2.73 | 1.33E-02 |  | *GRIN1* | 0.44 | | 0.18 | | 0.41 | 3.37E-05 |  |
| *DEPDC1* | 0.13 | 0.35 | 2.73 | 5.77E-05 |  | *ACSM1* | 0.06 | | 0.02 | | 0.41 | 3.24E-02 |  |
| *CENPU* | 0.34 | 0.93 | 2.73 | 1.24E-04 |  | *ATP1B2* | 0.10 | | 0.04 | | 0.41 | 1.44E-02 |  |
| *CHI3L1* | 0.13 | 0.34 | 2.73 | 1.69E-02 |  | *SERPINA3* | 0.07 | | 0.03 | | 0.41 | 6.85E-03 |  |
| *DDIAS* | 0.09 | 0.24 | 2.72 | 1.20E-04 |  | *MPEG1* | 1.23 | | 0.50 | | 0.41 | 3.35E-05 |  |
| *STRAP* | 1.39 | 3.80 | 2.72 | 3.38E-05 |  | *ARHGAP15* | 0.29 | | 0.12 | | 0.41 | 3.35E-05 |  |
| *NPR3* | 0.02 | 0.06 | 2.72 | 1.30E-02 |  | *KIAA0513* | 0.75 | | 0.31 | | 0.41 | 3.72E-05 |  |
| *ZNF367* | 0.21 | 0.57 | 2.72 | 1.21E-04 |  | *C1orf168* | 0.06 | | 0.02 | | 0.41 | 1.09E-02 |  |
| *FBL* | 2.23 | 6.08 | 2.72 | 9.06E-05 |  | *UNC5C* | 0.18 | | 0.07 | | 0.41 | 3.39E-05 |  |
| *IFITM2* | 1.49 | 4.06 | 2.72 | 1.77E-03 |  | *EPHA7* | 0.14 | | 0.06 | | 0.41 | 7.23E-05 |  |
| *TENM4* | 0.05 | 0.13 | 2.72 | 1.65E-03 |  | *NCAM1* | 0.16 | | 0.07 | | 0.41 | 5.44E-05 |  |
| *BLM* | 0.19 | 0.51 | 2.71 | 1.22E-04 |  | *CD38* | 0.39 | | 0.16 | | 0.41 | 3.57E-05 |  |
| *POLD2* | 0.61 | 1.66 | 2.71 | 3.37E-05 |  | *IKZF1* | 0.61 | | 0.25 | | 0.41 | 3.35E-05 |  |
| *GOLT1A* | 0.15 | 0.41 | 2.71 | 8.72E-03 |  | *ZAP70* | 0.26 | | 0.11 | | 0.41 | 2.36E-04 |  |
| *DUSP10* | 0.27 | 0.74 | 2.71 | 3.15E-02 |  | *CDKN2B* | 1.41 | | 0.58 | | 0.41 | 3.56E-05 |  |
| *DDX21* | 1.65 | 4.48 | 2.71 | 3.35E-05 |  | *NAAA* | 0.76 | | 0.32 | | 0.41 | 3.36E-05 |  |
| *CDC20* | 0.34 | 0.92 | 2.71 | 3.94E-03 |  | *ADH1A* | 0.12 | | 0.05 | | 0.41 | 9.25E-03 |  |
| *CASC5* | 0.28 | 0.75 | 2.71 | 3.39E-05 |  | *FABP1* | 7.45 | | 3.09 | | 0.41 | 3.35E-05 |  |
| *TFAP2C* | 0.06 | 0.16 | 2.71 | 3.69E-02 |  | *CPNE5* | 0.34 | | 0.14 | | 0.42 | 3.35E-05 |  |
| *MOSPD1* | 0.16 | 0.42 | 2.71 | 4.76E-05 |  | *PRR33* | 0.41 | | 0.17 | | 0.42 | 1.08E-04 |  |
| *HOMER1* | 0.12 | 0.33 | 2.71 | 4.00E-05 |  | *PTPRH* | 2.07 | | 0.86 | | 0.42 | 3.35E-05 |  |
| *GTSE1* | 0.18 | 0.50 | 2.71 | 1.40E-02 |  | *PSTPIP1* | 0.85 | | 0.35 | | 0.42 | 1.93E-04 |  |
| *PTRH1* | 0.08 | 0.23 | 2.70 | 1.91E-02 |  | *OTUD7A* | 0.11 | | 0.05 | | 0.42 | 5.90E-03 |  |
| *POLQ* | 0.23 | 0.63 | 2.70 | 6.31E-05 |  | *C6orf201* | 0.30 | | 0.12 | | 0.42 | 3.59E-05 |  |
| *AMOT* | 0.23 | 0.63 | 2.70 | 3.74E-05 |  | *SSBP2* | 0.25 | | 0.10 | | 0.42 | 3.35E-05 |  |
| *NOLC1* | 1.26 | 3.39 | 2.70 | 3.36E-05 |  | *RNF125* | 0.50 | | 0.21 | | 0.42 | 7.35E-05 |  |
| *EIF2S3* | 2.62 | 7.06 | 2.70 | 3.35E-05 |  | *KLK3* | 0.13 | | 0.06 | | 0.42 | 1.50E-03 |  |
| *ORC1* | 0.14 | 0.38 | 2.70 | 7.19E-03 |  | *CTD-2228K2.5* | 5.06 | | 2.13 | | 0.42 | 9.97E-05 |  |
| *C2* | 0.33 | 0.88 | 2.70 | 8.25E-05 |  | *BTK* | 0.24 | | 0.10 | | 0.42 | 9.44E-04 |  |
| *SSB* | 0.47 | 1.27 | 2.69 | 3.35E-05 |  | *CSF2RB* | 0.91 | | 0.38 | | 0.42 | 3.35E-05 |  |
| *HIST1H1B* | 1.48 | 3.98 | 2.69 | 5.12E-04 |  | *PRKACB* | 1.89 | | 0.80 | | 0.42 | 3.35E-05 |  |
| *KRT18* | 4.54 | 12.22 | 2.69 | 5.75E-05 |  | *CCDC141* | 0.11 | | 0.05 | | 0.42 | 3.50E-05 |  |
| *SPARC* | 3.01 | 8.11 | 2.69 | 3.35E-05 |  | *CCR2* | 0.15 | | 0.06 | | 0.42 | 7.72E-03 |  |
| *LMNB2* | 0.82 | 2.19 | 2.69 | 3.37E-05 |  | *RCAN2* | 0.50 | | 0.21 | | 0.42 | 8.58E-05 |  |
| *C10orf35* | 0.05 | 0.12 | 2.68 | 2.74E-02 |  | *ITGAL* | 0.89 | | 0.38 | | 0.42 | 5.29E-05 |  |
| *CCDC86* | 0.35 | 0.95 | 2.68 | 4.03E-05 |  | *ESR1* | 0.12 | | 0.05 | | 0.42 | 3.43E-05 |  |
| *GALNT6* | 0.45 | 1.19 | 2.68 | 1.82E-03 |  | *MFAP5* | 0.25 | | 0.11 | | 0.42 | 1.57E-02 |  |
| *MEX3D* | 0.44 | 1.18 | 2.67 | 2.83E-04 |  | *MYOCD* | 0.31 | | 0.13 | | 0.42 | 4.41E-05 |  |
| *ALCAM* | 0.43 | 1.15 | 2.67 | 4.57E-05 |  | *THEMIS* | 0.17 | | 0.07 | | 0.43 | 2.22E-03 |  |
| *NUFIP1* | 0.10 | 0.27 | 2.67 | 1.97E-02 |  | *CLDN5* | 0.11 | | 0.05 | | 0.43 | 3.19E-02 |  |
| *SOX9* | 1.91 | 5.09 | 2.67 | 3.35E-05 |  | *WNT2B* | 0.67 | | 0.29 | | 0.43 | 3.38E-05 |  |
| *WDR43* | 0.85 | 2.28 | 2.67 | 3.35E-05 |  | *STYK1* | 0.88 | | 0.37 | | 0.43 | 3.39E-05 |  |
| *MPP6* | 0.18 | 0.47 | 2.67 | 3.11E-04 |  | *FAM150B* | 0.23 | | 0.10 | | 0.43 | 5.27E-03 |  |
| *TGIF2* | 0.43 | 1.16 | 2.67 | 3.94E-05 |  | *ACACB* | 0.83 | | 0.36 | | 0.43 | 3.35E-05 |  |
| *CIRH1A* | 0.61 | 1.62 | 2.67 | 3.48E-05 |  | *CCL21* | 1.19 | | 0.51 | | 0.43 | 1.92E-03 |  |
| *SSX2IP* | 0.22 | 0.57 | 2.66 | 3.58E-05 |  | *CFD* | 1.05 | | 0.45 | | 0.43 | 3.88E-02 |  |
| *TMEM74B* | 0.18 | 0.49 | 2.66 | 4.96E-03 |  | *PPP1R16B* | 0.46 | | 0.20 | | 0.43 | 3.55E-05 |  |
| *AMACR* | 0.57 | 1.50 | 2.65 | 2.68E-02 |  | *SDK2* | 0.19 | | 0.08 | | 0.43 | 7.22E-04 |  |
| *NLE1* | 0.27 | 0.73 | 2.65 | 3.19E-02 |  | *MPZ* | 0.17 | | 0.07 | | 0.43 | 3.50E-04 |  |
| *RSL1D1* | 2.41 | 6.38 | 2.65 | 3.35E-05 |  | *TSPAN32* | 0.16 | | 0.07 | | 0.43 | 1.81E-04 |  |
| *ESPL1* | 0.18 | 0.47 | 2.65 | 1.58E-04 |  | *CCDC68* | 1.01 | | 0.43 | | 0.43 | 3.35E-05 |  |
| *SMS* | 0.69 | 1.83 | 2.64 | 3.36E-05 |  | *PLIN1* | 0.11 | | 0.05 | | 0.43 | 3.01E-04 |  |
| *AP1S3* | 0.15 | 0.39 | 2.64 | 4.61E-04 |  | *FIGN* | 0.10 | | 0.04 | | 0.43 | 4.86E-05 |  |
| *DEK* | 2.10 | 5.55 | 2.64 | 3.35E-05 |  | *PER1* | 1.30 | | 0.56 | | 0.43 | 3.45E-05 |  |
| *GAS1* | 0.10 | 0.26 | 2.64 | 9.95E-04 |  | *KCNH1* | 0.06 | | 0.03 | | 0.43 | 2.14E-02 |  |
| *PTPN13* | 0.24 | 0.62 | 2.64 | 7.39E-03 |  | *C1orf95* | 0.15 | | 0.07 | | 0.43 | 3.32E-04 |  |
| *CENPK* | 0.22 | 0.57 | 2.64 | 8.07E-05 |  | *HBB* | 0.99 | | 0.43 | | 0.43 | 2.90E-02 |  |
| *ZFP69B* | 0.08 | 0.22 | 2.64 | 1.92E-04 |  | *PTPRZ1* | 0.04 | | 0.02 | | 0.43 | 6.92E-03 |  |
| *JADE3* | 0.23 | 0.62 | 2.64 | 3.64E-05 |  | *KLF9* | 1.18 | | 0.51 | | 0.43 | 3.35E-05 |  |
| *HELLS* | 0.47 | 1.24 | 2.63 | 3.36E-05 |  | *P2RY1* | 0.69 | | 0.30 | | 0.43 | 3.68E-05 |  |
| *BRIP1* | 0.17 | 0.46 | 2.63 | 1.32E-03 |  | *PPP2R3A* | 0.59 | | 0.26 | | 0.43 | 3.35E-05 |  |
| *PPFIA4* | 0.05 | 0.12 | 2.63 | 1.08E-03 |  | *CR1* | 0.11 | | 0.05 | | 0.44 | 1.71E-02 |  |
| *PTP4A3* | 0.69 | 1.81 | 2.63 | 3.08E-02 |  | *OSR1* | 0.07 | | 0.03 | | 0.44 | 1.06E-02 |  |
| *MND1* | 0.16 | 0.42 | 2.63 | 3.24E-02 |  | *CCDC17* | 0.42 | | 0.18 | | 0.44 | 3.72E-05 |  |
| *CELSR1* | 0.33 | 0.86 | 2.63 | 3.36E-05 |  | *CD3E* | 0.41 | | 0.18 | | 0.44 | 7.49E-05 |  |
| *CBX3* | 1.05 | 2.76 | 2.63 | 3.35E-05 |  | *PAQR5* | 1.10 | | 0.48 | | 0.44 | 3.35E-05 |  |
| *HTRA3* | 0.31 | 0.81 | 2.63 | 2.58E-03 |  | *PIGZ* | 1.98 | | 0.87 | | 0.44 | 3.35E-05 |  |
| *HSPE1* | 0.41 | 1.07 | 2.62 | 2.52E-04 |  | *C2orf88* | 1.08 | | 0.47 | | 0.44 | 3.35E-05 |  |
| *PRDX4* | 0.53 | 1.39 | 2.62 | 4.56E-05 |  | *EAF2* | 0.42 | | 0.19 | | 0.44 | 1.73E-02 |  |
| *MTHFD2* | 0.61 | 1.60 | 2.61 | 3.39E-05 |  | *TLCD2* | 0.75 | | 0.33 | | 0.44 | 6.67E-03 |  |
| *BRCA2* | 0.25 | 0.65 | 2.61 | 3.49E-05 |  | *FLVCR2* | 0.50 | | 0.22 | | 0.44 | 2.21E-04 |  |
| *C9orf50* | 0.23 | 0.59 | 2.61 | 2.96E-03 |  | *SYNE3* | 0.44 | | 0.19 | | 0.44 | 6.96E-05 |  |
| *FXYD5* | 0.85 | 2.21 | 2.61 | 9.50E-05 |  | *C10orf99* | 4.95 | | 2.19 | | 0.44 | 3.37E-05 |  |
| *PTGES* | 0.19 | 0.49 | 2.61 | 1.18E-02 |  | *LSP1* | 1.07 | | 0.47 | | 0.44 | 3.35E-05 |  |
| *HECW1* | 0.03 | 0.08 | 2.60 | 4.55E-03 |  | *PLCG2* | 0.81 | | 0.36 | | 0.44 | 3.35E-05 |  |
| *TTYH3* | 1.34 | 3.50 | 2.60 | 3.37E-05 |  | *SMPD3* | 1.53 | | 0.68 | | 0.44 | 3.35E-05 |  |
| *KHK* | 0.13 | 0.35 | 2.60 | 3.93E-04 |  | *DUSP1* | 5.39 | | 2.39 | | 0.44 | 9.73E-05 |  |
| *LDHA* | 2.16 | 5.61 | 2.60 | 3.35E-05 |  | *LTB* | 0.30 | | 0.14 | | 0.44 | 1.03E-02 |  |
| *NOP56* | 0.65 | 1.68 | 2.59 | 3.36E-05 |  | *DOCK2* | 0.60 | | 0.27 | | 0.44 | 3.35E-05 |  |
| *PAK1IP1* | 0.35 | 0.91 | 2.59 | 3.82E-05 |  | *CCDC69* | 0.81 | | 0.36 | | 0.45 | 3.43E-05 |  |
| *TOP1MT* | 0.22 | 0.56 | 2.59 | 2.47E-03 |  | *STEAP4* | 0.26 | | 0.12 | | 0.45 | 2.78E-04 |  |
| *CCDC150* | 0.05 | 0.14 | 2.59 | 6.70E-04 |  | *FOS* | 14.28 | | 6.39 | | 0.45 | 3.47E-05 |  |
| *IRAK1* | 1.36 | 3.52 | 2.59 | 3.35E-05 |  | *RNLS* | 0.16 | | 0.07 | | 0.45 | 8.92E-05 |  |
| *IPO4* | 0.54 | 1.39 | 2.59 | 3.96E-03 |  | *MASP1* | 0.04 | | 0.02 | | 0.45 | 1.00E-02 |  |
| *STX1A* | 0.17 | 0.43 | 2.59 | 4.97E-04 |  | *CITED2* | 1.65 | | 0.74 | | 0.45 | 3.35E-05 |  |
| *CD276* | 0.66 | 1.71 | 2.58 | 3.36E-05 |  | *MIPOL1* | 0.25 | | 0.11 | | 0.45 | 3.38E-05 |  |
| *EIF1AX* | 0.47 | 1.22 | 2.58 | 1.25E-03 |  | *SYNPO* | 1.11 | | 0.50 | | 0.45 | 3.35E-05 |  |
| *SPATA12* | 0.15 | 0.38 | 2.58 | 4.81E-04 |  | *MCOLN2* | 0.68 | | 0.30 | | 0.45 | 5.76E-05 |  |
| *MROH6* | 0.34 | 0.88 | 2.58 | 1.98E-02 |  | *APBB1IP* | 0.30 | | 0.13 | | 0.45 | 1.96E-03 |  |
| *HIST1H2AI* | 1.06 | 2.74 | 2.58 | 2.85E-02 |  | *MYH3* | 0.11 | | 0.05 | | 0.45 | 1.41E-03 |  |
| *RPF2* | 0.36 | 0.94 | 2.58 | 5.59E-05 |  | *ST6GALNAC1* | 5.70 | | 2.58 | | 0.45 | 3.35E-05 |  |
| *HIST1H2AG* | 0.36 | 0.93 | 2.58 | 1.22E-02 |  | *SLC38A4* | 0.35 | | 0.16 | | 0.45 | 8.40E-05 |  |
| *CHTF18* | 0.23 | 0.60 | 2.58 | 2.05E-04 |  | *CLNK* | 0.07 | | 0.03 | | 0.45 | 1.88E-04 |  |
| *C9orf117* | 0.11 | 0.29 | 2.58 | 1.37E-03 |  | *PROB1* | 0.80 | | 0.36 | | 0.45 | 3.51E-05 |  |
| *IMPDH1* | 0.48 | 1.24 | 2.57 | 4.65E-05 |  | *PTGER2* | 0.49 | | 0.22 | | 0.46 | 6.12E-05 |  |
| *ZDHHC9* | 0.78 | 2.00 | 2.57 | 3.35E-05 |  | *FMO5* | 0.83 | | 0.38 | | 0.46 | 3.53E-05 |  |
| *GRB10* | 0.30 | 0.78 | 2.57 | 8.38E-05 |  | *MEF2C* | 0.51 | | 0.23 | | 0.46 | 3.35E-05 |  |
| *NDC80* | 0.22 | 0.56 | 2.57 | 1.59E-04 |  | *DDX26B* | 0.86 | | 0.40 | | 0.46 | 3.35E-05 |  |
| *RAB36* | 0.11 | 0.28 | 2.57 | 4.26E-02 |  | *STAB1* | 1.97 | | 0.90 | | 0.46 | 3.35E-05 |  |
| *MYL6B* | 0.28 | 0.71 | 2.57 | 3.61E-02 |  | *IKZF3* | 0.78 | | 0.36 | | 0.46 | 3.35E-05 |  |
| *LRP11* | 0.76 | 1.95 | 2.56 | 3.35E-05 |  | *PYGM* | 0.06 | | 0.03 | | 0.46 | 1.63E-02 |  |
| *RRM1* | 0.49 | 1.25 | 2.56 | 3.38E-05 |  | *CHI3L2* | 0.17 | | 0.08 | | 0.46 | 1.24E-02 |  |
| *WDR90* | 0.71 | 1.82 | 2.56 | 3.36E-05 |  | *ARHGAP9* | 0.76 | | 0.35 | | 0.46 | 7.57E-04 |  |
| *PODXL2* | 0.36 | 0.93 | 2.56 | 7.37E-03 |  | *IL24* | 0.30 | | 0.14 | | 0.46 | 4.85E-05 |  |
| *KIFC1* | 0.32 | 0.83 | 2.56 | 3.80E-05 |  | *ARHGAP28* | 0.10 | | 0.05 | | 0.46 | 1.39E-04 |  |
| *XKRX* | 0.13 | 0.32 | 2.56 | 1.22E-02 |  | *SOX10* | 0.18 | | 0.08 | | 0.46 | 7.70E-03 |  |
| *ADRM1* | 0.76 | 1.94 | 2.56 | 1.10E-03 |  | *COL4A4* | 0.30 | | 0.14 | | 0.46 | 3.35E-05 |  |
| *AP000350.4* | 1.02 | 2.60 | 2.56 | 2.78E-03 |  | *IL16* | 0.58 | | 0.27 | | 0.46 | 3.35E-05 |  |
| *C1orf198* | 0.49 | 1.26 | 2.55 | 3.37E-05 |  | *EFCC1* | 0.58 | | 0.27 | | 0.46 | 3.48E-05 |  |
| *LCTL* | 0.06 | 0.16 | 2.55 | 1.13E-02 |  | *KLRB1* | 0.43 | | 0.20 | | 0.46 | 1.81E-03 |  |
| *CNN2* | 1.96 | 5.00 | 2.55 | 3.35E-05 |  | *PID1* | 0.83 | | 0.39 | | 0.46 | 4.16E-05 |  |
| *POLR2I* | 0.42 | 1.08 | 2.55 | 3.03E-02 |  | *CNTN1* | 0.06 | | 0.03 | | 0.46 | 2.82E-02 |  |
| *TP53RK* | 0.32 | 0.81 | 2.55 | 8.81E-03 |  | *FGD2* | 0.36 | | 0.17 | | 0.46 | 3.35E-05 |  |
| *IRAK2* | 0.17 | 0.42 | 2.55 | 2.87E-03 |  | *SETBP1* | 0.51 | | 0.24 | | 0.46 | 3.35E-05 |  |
| *COL5A2* | 1.33 | 3.39 | 2.55 | 3.39E-05 |  | *ANKDD1B* | 0.54 | | 0.25 | | 0.46 | 1.31E-04 |  |
| *COL27A1* | 0.37 | 0.95 | 2.54 | 4.44E-05 |  | *A1CF* | 1.36 | | 0.64 | | 0.47 | 4.10E-05 |  |
| *HAUS1* | 0.35 | 0.88 | 2.54 | 1.40E-03 |  | *PAPSS2* | 2.86 | | 1.34 | | 0.47 | 3.35E-05 |  |
| *WDR3* | 0.45 | 1.13 | 2.54 | 3.35E-05 |  | *LMOD1* | 0.75 | | 0.35 | | 0.47 | 5.16E-04 |  |
| *GEMIN5* | 0.26 | 0.65 | 2.54 | 4.56E-05 |  | *SYT9* | 0.05 | | 0.02 | | 0.47 | 1.05E-02 |  |
| *TUBA1C* | 0.85 | 2.16 | 2.54 | 5.97E-05 |  | *TCEA3* | 1.98 | | 0.93 | | 0.47 | 3.37E-05 |  |
| *ZC3HAV1L* | 0.39 | 0.98 | 2.54 | 3.66E-05 |  | *ZFYVE28* | 0.43 | | 0.20 | | 0.47 | 3.35E-05 |  |
| *H2AFX* | 0.67 | 1.69 | 2.54 | 9.62E-04 |  | *TBC1D10C* | 0.43 | | 0.20 | | 0.47 | 3.38E-05 |  |
| *CD46* | 2.65 | 6.71 | 2.54 | 3.35E-05 |  | *C4orf19* | 1.25 | | 0.59 | | 0.47 | 3.35E-05 |  |
| *ETV1* | 0.07 | 0.17 | 2.53 | 1.31E-03 |  | *RBMS3* | 0.30 | | 0.14 | | 0.48 | 3.99E-05 |  |
| *RBL1* | 0.28 | 0.71 | 2.53 | 5.82E-05 |  | *FUCA1* | 2.24 | | 1.07 | | 0.48 | 4.57E-05 |  |
| *CCNB1IP1* | 0.57 | 1.43 | 2.52 | 5.11E-05 |  | *FLI1* | 0.40 | | 0.19 | | 0.48 | 3.35E-05 |  |
| *HIST1H2AD* | 1.27 | 3.20 | 2.52 | 1.26E-02 |  | *TRAF3IP3* | 0.46 | | 0.22 | | 0.48 | 3.35E-05 |  |
| *TFAP4* | 0.34 | 0.86 | 2.52 | 9.97E-04 |  | *TFCP2L1* | 2.53 | | 1.21 | | 0.48 | 3.35E-05 |  |
| *ATIC* | 0.73 | 1.84 | 2.52 | 3.35E-05 |  | *ATP8A1* | 1.81 | | 0.87 | | 0.48 | 3.35E-05 |  |
| *THY1* | 0.46 | 1.16 | 2.52 | 3.36E-05 |  | *XDH* | 0.92 | | 0.44 | | 0.48 | 3.35E-05 |  |
| *TIMELESS* | 0.35 | 0.89 | 2.52 | 7.65E-05 |  | *PDGFRL* | 0.56 | | 0.27 | | 0.48 | 6.58E-05 |  |
| *RP11-93B14.6* | 0.11 | 0.27 | 2.52 | 1.93E-02 |  | *ARHGAP25* | 0.31 | | 0.15 | | 0.49 | 4.04E-05 |  |
| *KIF3C* | 0.08 | 0.21 | 2.52 | 1.95E-03 |  | *CLIC5* | 1.08 | | 0.53 | | 0.49 | 3.35E-05 |  |
| *CDCA4* | 0.24 | 0.61 | 2.52 | 4.95E-04 |  | *SPARCL1* | 1.58 | | 0.77 | | 0.49 | 3.66E-05 |  |
| *MCM7* | 1.00 | 2.52 | 2.52 | 3.37E-05 |  | *FAM101A* | 0.78 | | 0.38 | | 0.49 | 1.23E-04 |  |
| *TXN* | 1.75 | 4.41 | 2.52 | 2.11E-03 |  | *SHE* | 0.17 | | 0.08 | | 0.49 | 6.86E-04 |  |
| *NSDHL* | 0.23 | 0.57 | 2.52 | 5.48E-03 |  |  |  | |  | |  |  |  |
| *EBPL* | 0.46 | 1.16 | 2.52 | 1.70E-04 |  |  |  | |  | |  |  |  |
| *PLIN2* | 0.24 | 0.62 | 2.52 | 8.37E-04 |  |  |  | |  | |  |  |  |
| *ASUN* | 0.41 | 1.04 | 2.51 | 8.70E-05 |  |  |  | |  | |  |  |  |
| *CCNB2* | 0.51 | 1.27 | 2.51 | 2.61E-03 |  |  |  | |  | |  |  |  |
| *CEP72* | 0.44 | 1.10 | 2.51 | 3.97E-04 |  |  |  | |  | |  |  |  |
| *CIT* | 0.25 | 0.64 | 2.51 | 5.48E-04 |  |  |  | |  | |  |  |  |
| *YAE1D1* | 0.30 | 0.75 | 2.51 | 2.38E-03 |  |  |  | |  | |  |  |  |
| *TARS* | 0.74 | 1.85 | 2.51 | 3.39E-05 |  |  |  | |  | |  |  |  |
| *KIF20B* | 0.35 | 0.88 | 2.51 | 3.46E-05 |  |  |  | |  | |  |  |  |
| *HIST1H3D* | 1.13 | 2.84 | 2.51 | 1.46E-02 |  |  |  | |  | |  |  |  |
| *TWISTNB* | 0.32 | 0.80 | 2.51 | 3.72E-05 |  |  |  | |  | |  |  |  |
| *RP11-385D13.1* | 0.07 | 0.18 | 2.51 | 2.28E-02 |  |  |  | |  | |  |  |  |
| *CTPS1* | 0.34 | 0.86 | 2.51 | 3.39E-05 |  |  |  | |  | |  |  |  |
| *POLR1B* | 0.34 | 0.85 | 2.50 | 3.38E-05 |  |  |  | |  | |  |  |  |
| *SRM* | 0.57 | 1.43 | 2.50 | 2.15E-04 |  |  |  | |  | |  |  |  |
| *NCS1* | 0.21 | 0.52 | 2.50 | 3.89E-05 |  |  |  | |  | |  |  |  |
| *PSPH* | 0.20 | 0.50 | 2.50 | 1.72E-03 |  |  |  | |  | |  |  |  |
| *LAMA5* | 0.80 | 1.99 | 2.49 | 4.28E-04 |  |  |  | |  | |  |  |  |
| *VBP1* | 0.43 | 1.07 | 2.49 | 3.33E-04 |  |  |  | |  | |  |  |  |
| *TPD52L1* | 0.16 | 0.39 | 2.49 | 9.01E-04 |  |  |  | |  | |  |  |  |
| *QPRT* | 0.57 | 1.41 | 2.49 | 2.69E-02 |  |  |  | |  | |  |  |  |
| *PLEKHB1* | 0.19 | 0.46 | 2.49 | 3.25E-02 |  |  |  | |  | |  |  |  |
| *EIF3E* | 1.90 | 4.72 | 2.48 | 3.35E-05 |  |  |  | |  | |  |  |  |
| *RMI2* | 0.35 | 0.87 | 2.48 | 1.88E-03 |  |  |  | |  | |  |  |  |
| *OXTR* | 0.04 | 0.09 | 2.47 | 1.16E-02 |  |  |  | |  | |  |  |  |
| *PCMTD2* | 0.82 | 2.03 | 2.47 | 1.34E-03 |  |  |  | |  | |  |  |  |
| *NOB1* | 0.80 | 1.98 | 2.47 | 3.75E-05 |  |  |  | |  | |  |  |  |
| *SKP2* | 0.45 | 1.11 | 2.47 | 7.71E-05 |  |  |  | |  | |  |  |  |
| *TMEM9* | 0.28 | 0.68 | 2.47 | 1.80E-03 |  |  |  | |  | |  |  |  |
| *CFB* | 0.45 | 1.10 | 2.47 | 5.41E-04 |  |  |  | |  | |  |  |  |
| *NAE1* | 0.95 | 2.35 | 2.47 | 2.99E-04 |  |  |  | |  | |  |  |  |
| *OAS3* | 0.98 | 2.41 | 2.47 | 3.35E-05 |  |  |  | |  | |  |  |  |
| *C17orf89* | 0.73 | 1.81 | 2.47 | 6.12E-04 |  |  |  | |  | |  |  |  |
| *DHCR7* | 0.28 | 0.70 | 2.47 | 3.01E-04 |  |  |  | |  | |  |  |  |
| *AGRN* | 0.82 | 2.01 | 2.47 | 3.35E-05 |  |  |  | |  | |  |  |  |
| *DDIT4* | 1.15 | 2.83 | 2.46 | 9.95E-04 |  |  |  | |  | |  |  |  |
| *MTBP* | 0.12 | 0.30 | 2.46 | 3.05E-03 |  |  |  | |  | |  |  |  |
| *TFDP1* | 1.06 | 2.61 | 2.46 | 3.35E-05 |  |  |  | |  | |  |  |  |
| *PSMG1* | 0.47 | 1.15 | 2.46 | 4.90E-04 |  |  |  | |  | |  |  |  |
| *BCL2L1* | 1.33 | 3.25 | 2.46 | 3.41E-05 |  |  |  | |  | |  |  |  |
| *SET* | 1.71 | 4.19 | 2.45 | 3.35E-05 |  |  |  | |  | |  |  |  |
| *HAPLN3* | 0.17 | 0.41 | 2.45 | 3.02E-02 |  |  |  | |  | |  |  |  |
| *SNRPC* | 0.50 | 1.22 | 2.45 | 1.42E-04 |  |  |  | |  | |  |  |  |
| *SAMD5* | 1.08 | 2.65 | 2.45 | 4.80E-03 |  |  |  | |  | |  |  |  |
| *LAPTM4B* | 0.74 | 1.81 | 2.45 | 6.90E-04 |  |  |  | |  | |  |  |  |
| *RAD54L* | 0.28 | 0.69 | 2.45 | 5.97E-03 |  |  |  | |  | |  |  |  |
| *VMA21* | 0.61 | 1.50 | 2.45 | 3.95E-05 |  |  |  | |  | |  |  |  |
| *PPAT* | 0.16 | 0.40 | 2.45 | 4.09E-05 |  |  |  | |  | |  |  |  |
| *CFB* | 0.70 | 1.71 | 2.45 | 8.03E-04 |  |  |  | |  | |  |  |  |
| *NCL* | 2.08 | 5.09 | 2.45 | 3.35E-05 |  |  |  | |  | |  |  |  |
| *WDR12* | 0.30 | 0.72 | 2.45 | 4.37E-05 |  |  |  | |  | |  |  |  |
| *PLXNA1* | 0.53 | 1.29 | 2.45 | 3.36E-05 |  |  |  | |  | |  |  |  |
| *FCGR3A* | 0.25 | 0.62 | 2.45 | 9.36E-04 |  |  |  | |  | |  |  |  |
| *FAM84B* | 0.76 | 1.85 | 2.45 | 3.36E-05 |  |  |  | |  | |  |  |  |
| *PALM2* | 0.07 | 0.17 | 2.45 | 2.22E-04 |  |  |  | |  | |  |  |  |
| *EIF2S2* | 0.94 | 2.31 | 2.45 | 3.49E-05 |  |  |  | |  | |  |  |  |
| *PRKDC* | 2.08 | 5.09 | 2.44 | 3.35E-05 |  |  |  | |  | |  |  |  |
| *FAM81A* | 0.10 | 0.25 | 2.44 | 4.55E-02 |  |  |  | |  | |  |  |  |
| *SMC2* | 0.44 | 1.06 | 2.44 | 5.45E-05 |  |  |  | |  | |  |  |  |
| *NUP62CL* | 0.17 | 0.42 | 2.44 | 2.59E-02 |  |  |  | |  | |  |  |  |
| *TNFAIP8L3* | 0.19 | 0.46 | 2.44 | 6.82E-03 |  |  |  | |  | |  |  |  |
| *FBXO41* | 0.25 | 0.60 | 2.44 | 1.91E-03 |  |  |  | |  | |  |  |  |
| *GJB3* | 0.22 | 0.53 | 2.44 | 5.54E-03 |  |  |  | |  | |  |  |  |
| *C12orf45* | 0.56 | 1.36 | 2.44 | 4.33E-05 |  |  |  | |  | |  |  |  |
| *RIPK2* | 0.27 | 0.65 | 2.43 | 3.95E-05 |  |  |  | |  | |  |  |  |
| *PDRG1* | 0.21 | 0.50 | 2.43 | 1.39E-02 |  |  |  | |  | |  |  |  |
| *GAPDH* | 10.44 | 25.40 | 2.43 | 3.39E-05 |  |  |  | |  | |  |  |  |
| *SPAG5* | 0.46 | 1.13 | 2.43 | 3.87E-03 |  |  |  | |  | |  |  |  |
| *C10orf2* | 0.21 | 0.50 | 2.43 | 1.77E-04 |  |  |  | |  | |  |  |  |
| *NXT1* | 0.19 | 0.47 | 2.43 | 3.37E-03 |  |  |  | |  | |  |  |  |
| *RRS1* | 0.58 | 1.42 | 2.43 | 4.54E-05 |  |  |  | |  | |  |  |  |
| *RNF219* | 0.28 | 0.69 | 2.43 | 3.68E-05 |  |  |  | |  | |  |  |  |
| *ALYREF* | 0.73 | 1.77 | 2.43 | 4.23E-05 |  |  |  | |  | |  |  |  |
| *PFDN2* | 0.49 | 1.18 | 2.42 | 7.70E-04 |  |  |  | |  | |  |  |  |
| *TLCD1* | 0.24 | 0.59 | 2.42 | 2.16E-03 |  |  |  | |  | |  |  |  |
| *PLAGL2* | 0.78 | 1.88 | 2.42 | 6.98E-03 |  |  |  | |  | |  |  |  |
| *TUBB* | 3.33 | 8.07 | 2.42 | 3.35E-05 |  |  |  | |  | |  |  |  |
| *ETV5* | 0.15 | 0.35 | 2.42 | 9.48E-05 |  |  |  | |  | |  |  |  |
| *SRPK1* | 1.66 | 4.01 | 2.42 | 3.35E-05 |  |  |  | |  | |  |  |  |
| *DNTTIP1* | 0.59 | 1.42 | 2.42 | 3.16E-04 |  |  |  | |  | |  |  |  |
| *SGOL1* | 0.15 | 0.37 | 2.42 | 1.61E-03 |  |  |  | |  | |  |  |  |
| *RUVBL2* | 0.54 | 1.31 | 2.41 | 4.59E-02 |  |  |  | |  | |  |  |  |
| *RNF128* | 0.46 | 1.11 | 2.41 | 4.14E-03 |  |  |  | |  | |  |  |  |
| *KLHL23* | 0.37 | 0.89 | 2.41 | 3.90E-05 |  |  |  | |  | |  |  |  |
| *ABHD2* | 1.69 | 4.06 | 2.41 | 3.35E-05 |  |  |  | |  | |  |  |  |
| *TIPIN* | 0.17 | 0.42 | 2.41 | 5.34E-03 |  |  |  | |  | |  |  |  |
| *ANTXR1* | 1.04 | 2.49 | 2.40 | 3.35E-05 |  |  |  | |  | |  |  |  |
| *C1QBP* | 1.62 | 3.88 | 2.40 | 1.65E-03 |  |  |  | |  | |  |  |  |
| *VCAN* | 0.72 | 1.73 | 2.40 | 3.36E-05 |  |  |  | |  | |  |  |  |
| *NUP155* | 0.53 | 1.28 | 2.40 | 4.38E-05 |  |  |  | |  | |  |  |  |
| *DBN1* | 0.29 | 0.69 | 2.40 | 5.58E-04 |  |  |  | |  | |  |  |  |
| *EPHB4* | 0.79 | 1.90 | 2.40 | 3.35E-05 |  |  |  | |  | |  |  |  |
| *RP11-101E3.5* | 0.58 | 1.38 | 2.40 | 3.90E-02 |  |  |  | |  | |  |  |  |
| *STXBP1* | 0.33 | 0.79 | 2.40 | 6.53E-04 |  |  |  | |  | |  |  |  |
| *IPO7* | 1.28 | 3.08 | 2.40 | 3.35E-05 |  |  |  | |  | |  |  |  |
| *CMSS1* | 0.25 | 0.60 | 2.40 | 1.10E-02 |  |  |  | |  | |  |  |  |
| *TM4SF1* | 0.68 | 1.63 | 2.40 | 5.32E-05 |  |  |  | |  | |  |  |  |
| *RFC4* | 0.19 | 0.46 | 2.40 | 4.12E-03 |  |  |  | |  | |  |  |  |
| *KIAA1524* | 0.25 | 0.60 | 2.40 | 1.25E-04 |  |  |  | |  | |  |  |  |
| *HAUS6* | 0.20 | 0.48 | 2.40 | 3.45E-05 |  |  |  | |  | |  |  |  |
| *EEF1E1* | 0.22 | 0.54 | 2.40 | 7.38E-04 |  |  |  | |  | |  |  |  |
| *RAB31* | 0.55 | 1.33 | 2.39 | 5.87E-05 |  |  |  | |  | |  |  |  |
| *MMP14* | 1.67 | 4.01 | 2.39 | 3.35E-05 |  |  |  | |  | |  |  |  |
| *PNPT1* | 0.44 | 1.06 | 2.39 | 5.84E-05 |  |  |  | |  | |  |  |  |
| *NIFK* | 0.51 | 1.23 | 2.39 | 3.42E-05 |  |  |  | |  | |  |  |  |
| *LSM5* | 0.27 | 0.66 | 2.39 | 3.35E-05 |  |  |  | |  | |  |  |  |
| *GRAMD1A* | 0.88 | 2.11 | 2.39 | 1.13E-03 |  |  |  | |  | |  |  |  |
| *LDLRAD3* | 0.17 | 0.40 | 2.39 | 9.18E-03 |  |  |  | |  | |  |  |  |
| *DNAJA1* | 1.38 | 3.30 | 2.39 | 3.35E-05 |  |  |  | |  | |  |  |  |
| *DKFZP686D09174* | 0.52 | 1.24 | 2.39 | 3.47E-05 |  |  |  | |  | |  |  |  |
| *PHLDA2* | 0.49 | 1.17 | 2.39 | 1.68E-02 |  |  |  | |  | |  |  |  |
| *CCT5* | 0.84 | 2.00 | 2.39 | 3.35E-05 |  |  |  | |  | |  |  |  |
| *LTV1* | 0.44 | 1.06 | 2.39 | 2.29E-04 |  |  |  | |  | |  |  |  |
| *KARS* | 1.61 | 3.84 | 2.38 | 3.35E-05 |  |  |  | |  | |  |  |  |
| *RCC1* | 0.71 | 1.69 | 2.38 | 3.46E-05 |  |  |  | |  | |  |  |  |
| *RNFT2* | 0.06 | 0.15 | 2.38 | 8.01E-03 |  |  |  | |  | |  |  |  |
| *PLEK2* | 0.88 | 2.10 | 2.38 | 4.37E-05 |  |  |  | |  | |  |  |  |
| *HN1L* | 1.12 | 2.65 | 2.38 | 3.35E-05 |  |  |  | |  | |  |  |  |
| *MANEAL* | 0.23 | 0.55 | 2.38 | 1.35E-02 |  |  |  | |  | |  |  |  |
| *CDH24* | 0.19 | 0.45 | 2.38 | 6.95E-03 |  |  |  | |  | |  |  |  |
| *MEST* | 0.58 | 1.37 | 2.38 | 3.61E-05 |  |  |  | |  | |  |  |  |
| *ESCO2* | 0.10 | 0.24 | 2.38 | 5.64E-03 |  |  |  | |  | |  |  |  |
| *DNA2* | 0.20 | 0.49 | 2.38 | 2.23E-04 |  |  |  | |  | |  |  |  |
| *SIGMAR1* | 1.14 | 2.70 | 2.37 | 3.38E-05 |  |  |  | |  | |  |  |  |
| *RPN2* | 2.88 | 6.81 | 2.37 | 3.39E-05 |  |  |  | |  | |  |  |  |
| *SLC5A6* | 0.48 | 1.14 | 2.37 | 3.36E-05 |  |  |  | |  | |  |  |  |
| *GRINA* | 1.57 | 3.73 | 2.37 | 3.82E-05 |  |  |  | |  | |  |  |  |
| *CD44* | 2.11 | 5.00 | 2.37 | 3.35E-05 |  |  |  | |  | |  |  |  |
| *ASF1B* | 0.33 | 0.79 | 2.36 | 7.66E-03 |  |  |  | |  | |  |  |  |
| *KDM1A* | 1.36 | 3.21 | 2.36 | 3.46E-05 |  |  |  | |  | |  |  |  |
| *HSD17B10* | 0.59 | 1.39 | 2.36 | 3.26E-03 |  |  |  | |  | |  |  |  |
| *PA2G4* | 1.48 | 3.50 | 2.36 | 3.94E-05 |  |  |  | |  | |  |  |  |
| *C11orf95* | 0.26 | 0.62 | 2.36 | 3.78E-04 |  |  |  | |  | |  |  |  |
| *RANBP1* | 0.31 | 0.74 | 2.36 | 4.67E-02 |  |  |  | |  | |  |  |  |
| *NME1-NME2* | 4.17 | 9.83 | 2.36 | 3.35E-05 |  |  |  | |  | |  |  |  |
| *NLN* | 0.20 | 0.47 | 2.36 | 3.75E-05 |  |  |  | |  | |  |  |  |
| *MOCS3* | 0.18 | 0.41 | 2.36 | 2.07E-03 |  |  |  | |  | |  |  |  |
| *SLC39A6* | 0.44 | 1.03 | 2.35 | 3.07E-04 |  |  |  | |  | |  |  |  |
| *COL3A1* | 10.26 | 24.14 | 2.35 | 3.35E-05 |  |  |  | |  | |  |  |  |
| *PPP1R14B* | 0.82 | 1.92 | 2.35 | 1.85E-02 |  |  |  | |  | |  |  |  |
| *NKRF* | 0.26 | 0.61 | 2.35 | 9.75E-05 |  |  |  | |  | |  |  |  |
| *NUDCD1* | 0.27 | 0.64 | 2.35 | 6.75E-05 |  |  |  | |  | |  |  |  |
| *CDH11* | 0.45 | 1.07 | 2.35 | 4.12E-05 |  |  |  | |  | |  |  |  |
| *KCNH8* | 0.13 | 0.31 | 2.35 | 1.22E-02 |  |  |  | |  | |  |  |  |
| *GSTP1* | 2.04 | 4.80 | 2.35 | 8.01E-05 |  |  |  | |  | |  |  |  |
| *SUGT1* | 0.74 | 1.74 | 2.35 | 9.71E-04 |  |  |  | |  | |  |  |  |
| *BCAN* | 0.21 | 0.49 | 2.34 | 2.42E-02 |  |  |  | |  | |  |  |  |
| *CHPF* | 0.82 | 1.91 | 2.34 | 3.51E-05 |  |  |  | |  | |  |  |  |
| *FOXRED2* | 0.21 | 0.49 | 2.34 | 2.69E-04 |  |  |  | |  | |  |  |  |
| *NOP2* | 0.40 | 0.95 | 2.34 | 1.79E-03 |  |  |  | |  | |  |  |  |
| *SRD5A1* | 0.23 | 0.53 | 2.34 | 4.21E-04 |  |  |  | |  | |  |  |  |
| *PGK1* | 1.64 | 3.83 | 2.34 | 3.60E-05 |  |  |  | |  | |  |  |  |
| *CPNE1* | 1.33 | 3.12 | 2.34 | 2.20E-04 |  |  |  | |  | |  |  |  |
| *NQO1* | 0.98 | 2.29 | 2.34 | 6.20E-04 |  |  |  | |  | |  |  |  |
| *RFWD3* | 0.60 | 1.40 | 2.34 | 3.78E-05 |  |  |  | |  | |  |  |  |
| *CCT7* | 1.03 | 2.40 | 2.34 | 3.35E-05 |  |  |  | |  | |  |  |  |
| *P4HA1* | 0.87 | 2.02 | 2.34 | 8.18E-05 |  |  |  | |  | |  |  |  |
| *SHH* | 0.19 | 0.45 | 2.34 | 4.92E-02 |  |  |  | |  | |  |  |  |
| *EIF4A3* | 0.73 | 1.71 | 2.34 | 5.87E-05 |  |  |  | |  | |  |  |  |
| *CYB5B* | 1.03 | 2.41 | 2.33 | 3.51E-05 |  |  |  | |  | |  |  |  |
| *MCM8* | 0.44 | 1.03 | 2.33 | 7.84E-04 |  |  |  | |  | |  |  |  |
| *ANKRD30BL* | 1.78 | 4.16 | 2.33 | 3.39E-05 |  |  |  | |  | |  |  |  |
| *PRC1* | 0.62 | 1.44 | 2.33 | 3.80E-05 |  |  |  | |  | |  |  |  |
| *NME2* | 2.09 | 4.88 | 2.33 | 3.35E-05 |  |  |  | |  | |  |  |  |
| *RPL23* | 3.22 | 7.50 | 2.33 | 3.38E-05 |  |  |  | |  | |  |  |  |
| *C12orf5* | 0.27 | 0.62 | 2.33 | 4.28E-04 |  |  |  | |  | |  |  |  |
| *ZNF503* | 0.23 | 0.53 | 2.33 | 7.31E-04 |  |  |  | |  | |  |  |  |
| *DSN1* | 0.34 | 0.78 | 2.33 | 2.86E-03 |  |  |  | |  | |  |  |  |
| *UBA2* | 1.10 | 2.56 | 2.33 | 3.35E-05 |  |  |  | |  | |  |  |  |
| *MID1IP1* | 0.41 | 0.97 | 2.33 | 2.31E-03 |  |  |  | |  | |  |  |  |
| *C11orf84* | 0.27 | 0.62 | 2.33 | 1.38E-03 |  |  |  | |  | |  |  |  |
| *RTKN* | 0.60 | 1.39 | 2.33 | 8.94E-05 |  |  |  | |  | |  |  |  |
| *HDAC2* | 0.27 | 0.64 | 2.33 | 3.82E-04 |  |  |  | |  | |  |  |  |
| *EPDR1* | 0.26 | 0.61 | 2.32 | 8.87E-03 |  |  |  | |  | |  |  |  |
| *ZC3H15* | 1.17 | 2.72 | 2.32 | 3.39E-05 |  |  |  | |  | |  |  |  |
| *MRPL36* | 0.13 | 0.30 | 2.32 | 1.34E-02 |  |  |  | |  | |  |  |  |
| *OLFML2B* | 0.17 | 0.39 | 2.32 | 3.50E-04 |  |  |  | |  | |  |  |  |
| *TKT* | 0.70 | 1.62 | 2.32 | 3.35E-05 |  |  |  | |  | |  |  |  |
| *LMNB1* | 0.89 | 2.07 | 2.32 | 3.47E-05 |  |  |  | |  | |  |  |  |
| *TK1* | 0.54 | 1.25 | 2.32 | 5.77E-04 |  |  |  | |  | |  |  |  |
| *DCUN1D5* | 0.42 | 0.98 | 2.32 | 3.97E-03 |  |  |  | |  | |  |  |  |
| *TRIM28* | 2.50 | 5.81 | 2.32 | 5.46E-05 |  |  |  | |  | |  |  |  |
| *CKAP5* | 0.93 | 2.16 | 2.32 | 3.35E-05 |  |  |  | |  | |  |  |  |
| *CDC123* | 0.76 | 1.76 | 2.32 | 3.42E-05 |  |  |  | |  | |  |  |  |
| *DNAJB1* | 1.60 | 3.72 | 2.32 | 7.25E-04 |  |  |  | |  | |  |  |  |
| *MCTS1* | 0.31 | 0.73 | 2.32 | 8.29E-03 |  |  |  | |  | |  |  |  |
| *EIF3B* | 0.89 | 2.07 | 2.32 | 3.35E-05 |  |  |  | |  | |  |  |  |
| *SPIN4* | 0.22 | 0.50 | 2.32 | 4.79E-04 |  |  |  | |  | |  |  |  |
| *CAD* | 0.63 | 1.46 | 2.32 | 3.35E-05 |  |  |  | |  | |  |  |  |
| *CSTF2* | 0.29 | 0.67 | 2.31 | 4.14E-03 |  |  |  | |  | |  |  |  |
| *TIMM50* | 0.49 | 1.14 | 2.31 | 7.82E-03 |  |  |  | |  | |  |  |  |
| *TOMM20* | 1.68 | 3.89 | 2.31 | 3.35E-05 |  |  |  | |  | |  |  |  |
| *MTCL1* | 0.22 | 0.52 | 2.31 | 3.13E-02 |  |  |  | |  | |  |  |  |
| *FRMD5* | 0.15 | 0.36 | 2.31 | 8.06E-03 |  |  |  | |  | |  |  |  |
| *PPL* | 0.39 | 0.91 | 2.31 | 1.01E-03 |  |  |  | |  | |  |  |  |
| *OIP5* | 0.19 | 0.45 | 2.31 | 2.84E-02 |  |  |  | |  | |  |  |  |
| *EBNA1BP2* | 0.34 | 0.78 | 2.31 | 1.37E-04 |  |  |  | |  | |  |  |  |
| *ABCE1* | 0.51 | 1.18 | 2.31 | 3.35E-05 |  |  |  | |  | |  |  |  |
| *SNRPB* | 1.24 | 2.87 | 2.31 | 3.51E-05 |  |  |  | |  | |  |  |  |
| *C20orf24* | 1.34 | 3.09 | 2.31 | 5.09E-05 |  |  |  | |  | |  |  |  |
| *PRMT1* | 0.44 | 1.01 | 2.31 | 5.48E-04 |  |  |  | |  | |  |  |  |
| *CCDC138* | 0.18 | 0.41 | 2.31 | 6.52E-04 |  |  |  | |  | |  |  |  |
| *CCT8* | 0.90 | 2.07 | 2.31 | 5.75E-05 |  |  |  | |  | |  |  |  |
| *MTHFD1* | 0.86 | 1.98 | 2.31 | 4.02E-05 |  |  |  | |  | |  |  |  |
| *MALSU1* | 0.18 | 0.41 | 2.31 | 2.35E-03 |  |  |  | |  | |  |  |  |
| *RTN4RL2* | 0.05 | 0.12 | 2.31 | 3.46E-02 |  |  |  | |  | |  |  |  |
| *FIGNL1* | 0.25 | 0.57 | 2.30 | 3.95E-04 |  |  |  | |  | |  |  |  |
| *HDGF* | 2.74 | 6.30 | 2.30 | 3.35E-05 |  |  |  | |  | |  |  |  |
| *EEPD1* | 0.32 | 0.73 | 2.30 | 3.69E-04 |  |  |  | |  | |  |  |  |
| *RAB15* | 0.88 | 2.03 | 2.30 | 2.22E-04 |  |  |  | |  | |  |  |  |
| *FTSJ2* | 0.27 | 0.61 | 2.30 | 6.34E-04 |  |  |  | |  | |  |  |  |
| *DBF4* | 0.18 | 0.42 | 2.30 | 4.08E-05 |  |  |  | |  | |  |  |  |
| *MSANTD3* | 0.21 | 0.47 | 2.30 | 1.17E-03 |  |  |  | |  | |  |  |  |
| *SNRPB2* | 0.51 | 1.17 | 2.30 | 8.44E-05 |  |  |  | |  | |  |  |  |
| *POLE2* | 0.22 | 0.50 | 2.29 | 2.42E-03 |  |  |  | |  | |  |  |  |
| *SLC7A1* | 0.97 | 2.23 | 2.29 | 3.35E-05 |  |  |  | |  | |  |  |  |
| *TRAP1* | 1.47 | 3.38 | 2.29 | 1.75E-04 |  |  |  | |  | |  |  |  |
| *MRPS35* | 1.13 | 2.59 | 2.29 | 3.93E-04 |  |  |  | |  | |  |  |  |
| *CCNE1* | 0.16 | 0.36 | 2.29 | 3.91E-02 |  |  |  | |  | |  |  |  |
| *RRP9* | 0.38 | 0.86 | 2.29 | 8.01E-03 |  |  |  | |  | |  |  |  |
| *CSTF1* | 0.37 | 0.85 | 2.29 | 8.85E-04 |  |  |  | |  | |  |  |  |
| *KIAA1430* | 0.50 | 1.15 | 2.29 | 3.40E-05 |  |  |  | |  | |  |  |  |
| *SERPINH1* | 0.72 | 1.65 | 2.29 | 3.58E-05 |  |  |  | |  | |  |  |  |
| *C16orf13* | 0.92 | 2.10 | 2.29 | 5.68E-03 |  |  |  | |  | |  |  |  |
| *BHLHE40* | 1.87 | 4.29 | 2.29 | 6.06E-05 |  |  |  | |  | |  |  |  |
| *AADAT* | 0.15 | 0.34 | 2.29 | 9.53E-04 |  |  |  | |  | |  |  |  |
| *ACSL1* | 0.36 | 0.82 | 2.29 | 4.16E-05 |  |  |  | |  | |  |  |  |
| *C6orf48* | 1.02 | 2.32 | 2.29 | 7.54E-05 |  |  |  | |  | |  |  |  |
| *NOP58* | 1.12 | 2.55 | 2.29 | 3.35E-05 |  |  |  | |  | |  |  |  |
| *POP7* | 0.39 | 0.89 | 2.29 | 1.37E-02 |  |  |  | |  | |  |  |  |
| *NANP* | 0.17 | 0.40 | 2.29 | 8.85E-04 |  |  |  | |  | |  |  |  |
| *AP3M2* | 0.22 | 0.51 | 2.28 | 3.11E-02 |  |  |  | |  | |  |  |  |
| *YWHAG* | 2.25 | 5.13 | 2.28 | 3.35E-05 |  |  |  | |  | |  |  |  |
| *NCAPG* | 0.33 | 0.75 | 2.28 | 1.51E-03 |  |  |  | |  | |  |  |  |
| *C20orf27* | 0.33 | 0.75 | 2.28 | 3.40E-04 |  |  |  | |  | |  |  |  |
| *FAM83H* | 1.53 | 3.48 | 2.28 | 5.97E-05 |  |  |  | |  | |  |  |  |
| *NSA2* | 0.70 | 1.58 | 2.28 | 9.86E-05 |  |  |  | |  | |  |  |  |
| *NT5DC3* | 0.51 | 1.17 | 2.28 | 1.08E-04 |  |  |  | |  | |  |  |  |
| *CENPM* | 0.22 | 0.50 | 2.28 | 4.05E-02 |  |  |  | |  | |  |  |  |
| *BZW2* | 0.92 | 2.10 | 2.28 | 3.38E-05 |  |  |  | |  | |  |  |  |
| *RCN2* | 0.62 | 1.41 | 2.27 | 2.39E-03 |  |  |  | |  | |  |  |  |
| *DARS* | 0.78 | 1.77 | 2.27 | 3.35E-05 |  |  |  | |  | |  |  |  |
| *RPL4* | 6.72 | 15.27 | 2.27 | 3.35E-05 |  |  |  | |  | |  |  |  |
| *PRPS2* | 0.78 | 1.78 | 2.27 | 1.55E-04 |  |  |  | |  | |  |  |  |
| *WDR34* | 0.61 | 1.38 | 2.27 | 1.78E-02 |  |  |  | |  | |  |  |  |
| *WDR35* | 0.15 | 0.35 | 2.27 | 1.52E-03 |  |  |  | |  | |  |  |  |
| *ASPH* | 1.07 | 2.43 | 2.27 | 3.35E-05 |  |  |  | |  | |  |  |  |
| *TUBA1B* | 1.23 | 2.80 | 2.27 | 3.35E-05 |  |  |  | |  | |  |  |  |
| *TGS1* | 0.49 | 1.10 | 2.27 | 3.06E-04 |  |  |  | |  | |  |  |  |
| *EPB41L2* | 1.50 | 3.39 | 2.27 | 3.46E-05 |  |  |  | |  | |  |  |  |
| *SLIRP* | 1.35 | 3.05 | 2.27 | 1.82E-04 |  |  |  | |  | |  |  |  |
| *SCLY* | 0.15 | 0.35 | 2.27 | 3.59E-05 |  |  |  | |  | |  |  |  |
| *CPSF3* | 0.42 | 0.95 | 2.27 | 2.58E-04 |  |  |  | |  | |  |  |  |
| *PSMD14* | 0.32 | 0.72 | 2.27 | 5.23E-05 |  |  |  | |  | |  |  |  |
| *ASNSD1* | 0.56 | 1.26 | 2.27 | 1.35E-04 |  |  |  | |  | |  |  |  |
| *CCDC59* | 0.40 | 0.91 | 2.26 | 5.35E-03 |  |  |  | |  | |  |  |  |
| *GLRX3* | 0.62 | 1.39 | 2.26 | 1.64E-04 |  |  |  | |  | |  |  |  |
| *CSTB* | 1.01 | 2.29 | 2.26 | 4.19E-05 |  |  |  | |  | |  |  |  |
| *FANCI* | 0.64 | 1.44 | 2.26 | 5.38E-05 |  |  |  | |  | |  |  |  |
| *RPL26* | 10.66 | 24.12 | 2.26 | 3.45E-05 |  |  |  | |  | |  |  |  |
| *RPL14* | 1.85 | 4.18 | 2.26 | 3.47E-05 |  |  |  | |  | |  |  |  |
| *ITGB8* | 0.32 | 0.72 | 2.26 | 2.58E-03 |  |  |  | |  | |  |  |  |
| *PSMA2* | 0.39 | 0.88 | 2.26 | 3.64E-05 |  |  |  | |  | |  |  |  |
| *ICT1* | 0.49 | 1.11 | 2.26 | 2.52E-03 |  |  |  | |  | |  |  |  |
| *GMPS* | 0.67 | 1.50 | 2.26 | 3.37E-05 |  |  |  | |  | |  |  |  |
| *MIS18A* | 0.27 | 0.60 | 2.25 | 2.48E-03 |  |  |  | |  | |  |  |  |
| *S100A6* | 10.02 | 22.57 | 2.25 | 4.70E-05 |  |  |  | |  | |  |  |  |
| *RPL27* | 13.57 | 30.55 | 2.25 | 3.35E-05 |  |  |  | |  | |  |  |  |
| *DPY19L1* | 0.41 | 0.92 | 2.25 | 3.91E-05 |  |  |  | |  | |  |  |  |
| *DTD1* | 0.25 | 0.56 | 2.25 | 4.92E-04 |  |  |  | |  | |  |  |  |
| *VRK1* | 0.45 | 1.02 | 2.25 | 3.80E-05 |  |  |  | |  | |  |  |  |
| *PRDX1* | 2.40 | 5.38 | 2.24 | 3.46E-05 |  |  |  | |  | |  |  |  |
| *TSN* | 0.79 | 1.77 | 2.24 | 3.40E-05 |  |  |  | |  | |  |  |  |
| *IARS* | 1.27 | 2.84 | 2.24 | 3.37E-05 |  |  |  | |  | |  |  |  |
| *FLVCR1* | 0.31 | 0.70 | 2.24 | 3.35E-05 |  |  |  | |  | |  |  |  |
| *PYCRL* | 0.22 | 0.50 | 2.24 | 3.38E-03 |  |  |  | |  | |  |  |  |
| *PBDC1* | 0.36 | 0.80 | 2.24 | 4.22E-03 |  |  |  | |  | |  |  |  |
| *CTNNAL1* | 0.33 | 0.73 | 2.24 | 1.10E-02 |  |  |  | |  | |  |  |  |
| *NCAPD2* | 0.81 | 1.82 | 2.24 | 3.36E-05 |  |  |  | |  | |  |  |  |
| *CSNK2A2* | 0.99 | 2.22 | 2.24 | 1.11E-03 |  |  |  | |  | |  |  |  |
| *PTPN11* | 1.14 | 2.55 | 2.23 | 3.35E-05 |  |  |  | |  | |  |  |  |
| *SLC4A2* | 0.56 | 1.24 | 2.23 | 3.65E-05 |  |  |  | |  | |  |  |  |
| *FAM217B* | 0.24 | 0.53 | 2.23 | 2.44E-03 |  |  |  | |  | |  |  |  |
| *NUTF2* | 0.67 | 1.50 | 2.23 | 3.11E-03 |  |  |  | |  | |  |  |  |
| *SNRNP25* | 0.14 | 0.31 | 2.23 | 3.76E-03 |  |  |  | |  | |  |  |  |
| *TUBG1* | 0.35 | 0.77 | 2.23 | 1.95E-02 |  |  |  | |  | |  |  |  |
| *ZNF280C* | 0.16 | 0.36 | 2.23 | 5.25E-03 |  |  |  | |  | |  |  |  |
| *MRPL3* | 0.95 | 2.11 | 2.23 | 3.52E-05 |  |  |  | |  | |  |  |  |
| *NELFCD* | 0.56 | 1.25 | 2.23 | 3.42E-05 |  |  |  | |  | |  |  |  |
| *TCOF1* | 0.39 | 0.87 | 2.23 | 6.91E-05 |  |  |  | |  | |  |  |  |
| *POLA1* | 0.43 | 0.95 | 2.23 | 5.46E-03 |  |  |  | |  | |  |  |  |
| *RUNX1* | 0.64 | 1.42 | 2.23 | 3.35E-05 |  |  |  | |  | |  |  |  |
| *URB2* | 0.30 | 0.67 | 2.22 | 4.60E-05 |  |  |  | |  | |  |  |  |
| *KCNJ14* | 0.12 | 0.27 | 2.22 | 1.85E-02 |  |  |  | |  | |  |  |  |
| *MAPRE1* | 1.55 | 3.45 | 2.22 | 3.40E-05 |  |  |  | |  | |  |  |  |
| *CLDN12* | 0.70 | 1.57 | 2.22 | 4.81E-05 |  |  |  | |  | |  |  |  |
| *PAIP1* | 0.38 | 0.84 | 2.22 | 3.23E-04 |  |  |  | |  | |  |  |  |
| *HTRA1* | 0.55 | 1.22 | 2.22 | 1.27E-03 |  |  |  | |  | |  |  |  |
| *CLVS1* | 0.08 | 0.18 | 2.22 | 8.17E-03 |  |  |  | |  | |  |  |  |
| *IFT52* | 0.33 | 0.73 | 2.22 | 3.88E-04 |  |  |  | |  | |  |  |  |
| *GINS3* | 0.14 | 0.31 | 2.22 | 1.60E-02 |  |  |  | |  | |  |  |  |
| *ERO1L* | 1.63 | 3.62 | 2.22 | 3.35E-05 |  |  |  | |  | |  |  |  |
| *CHRNA5* | 0.09 | 0.19 | 2.22 | 6.79E-04 |  |  |  | |  | |  |  |  |
| *ENAH* | 0.65 | 1.45 | 2.22 | 3.45E-05 |  |  |  | |  | |  |  |  |
| *PSTPIP2* | 0.22 | 0.49 | 2.22 | 7.18E-03 |  |  |  | |  | |  |  |  |
| *TSR1* | 0.53 | 1.17 | 2.22 | 6.36E-04 |  |  |  | |  | |  |  |  |
| *PRR7* | 0.19 | 0.43 | 2.21 | 4.50E-02 |  |  |  | |  | |  |  |  |
| *NOTCH3* | 0.73 | 1.61 | 2.21 | 3.58E-05 |  |  |  | |  | |  |  |  |
| *EFNA4* | 0.47 | 1.03 | 2.21 | 1.02E-03 |  |  |  | |  | |  |  |  |
| *TMEM147* | 0.72 | 1.59 | 2.21 | 8.90E-04 |  |  |  | |  | |  |  |  |
| *SLC3A2* | 0.66 | 1.46 | 2.21 | 1.66E-04 |  |  |  | |  | |  |  |  |
| *MMS22L* | 0.16 | 0.35 | 2.21 | 1.74E-04 |  |  |  | |  | |  |  |  |
| *RRP1B* | 0.85 | 1.88 | 2.21 | 3.37E-05 |  |  |  | |  | |  |  |  |
| *FANCG* | 0.22 | 0.49 | 2.21 | 2.78E-02 |  |  |  | |  | |  |  |  |
| *IL1RN* | 0.15 | 0.33 | 2.21 | 5.12E-03 |  |  |  | |  | |  |  |  |
| *LBR* | 1.51 | 3.32 | 2.21 | 3.35E-05 |  |  |  | |  | |  |  |  |
| *HMGB2* | 0.65 | 1.42 | 2.21 | 5.75E-03 |  |  |  | |  | |  |  |  |
| *RFC2* | 0.34 | 0.75 | 2.20 | 2.19E-02 |  |  |  | |  | |  |  |  |
| *RPS4X* | 6.20 | 13.65 | 2.20 | 3.36E-05 |  |  |  | |  | |  |  |  |
| *AP1S1* | 0.99 | 2.17 | 2.20 | 8.46E-04 |  |  |  | |  | |  |  |  |
| *LIPG* | 0.67 | 1.48 | 2.20 | 4.33E-02 |  |  |  | |  | |  |  |  |
| *GPR56* | 1.34 | 2.95 | 2.20 | 3.35E-05 |  |  |  | |  | |  |  |  |
| *CDK6* | 0.96 | 2.12 | 2.20 | 3.38E-05 |  |  |  | |  | |  |  |  |
| *RPL36* | 13.33 | 29.26 | 2.20 | 3.67E-05 |  |  |  | |  | |  |  |  |
| *POLE3* | 0.56 | 1.22 | 2.20 | 2.82E-04 |  |  |  | |  | |  |  |  |
| *CBFB* | 0.89 | 1.96 | 2.19 | 4.06E-05 |  |  |  | |  | |  |  |  |
| *ZNF74* | 0.15 | 0.34 | 2.19 | 3.59E-02 |  |  |  | |  | |  |  |  |
| *OSER1* | 0.77 | 1.69 | 2.19 | 3.41E-02 |  |  |  | |  | |  |  |  |
| *RTKN2* | 0.14 | 0.30 | 2.19 | 1.74E-02 |  |  |  | |  | |  |  |  |
| *SMC4* | 0.93 | 2.05 | 2.19 | 3.35E-05 |  |  |  | |  | |  |  |  |
| *KIAA0020* | 0.95 | 2.07 | 2.19 | 1.82E-04 |  |  |  | |  | |  |  |  |
| *SUPT16H* | 1.02 | 2.23 | 2.19 | 3.42E-05 |  |  |  | |  | |  |  |  |
| *CLIC1* | 3.42 | 7.46 | 2.19 | 3.37E-05 |  |  |  | |  | |  |  |  |
| *FAM60A* | 0.54 | 1.17 | 2.18 | 4.70E-05 |  |  |  | |  | |  |  |  |
| *ACTL6A* | 0.46 | 1.01 | 2.18 | 2.05E-04 |  |  |  | |  | |  |  |  |
| *EIF3I* | 1.46 | 3.18 | 2.18 | 8.38E-05 |  |  |  | |  | |  |  |  |
| *TMEM237* | 0.10 | 0.23 | 2.18 | 3.33E-04 |  |  |  | |  | |  |  |  |
| *LIF* | 0.73 | 1.60 | 2.18 | 9.75E-04 |  |  |  | |  | |  |  |  |
| *ATP6V1F* | 1.31 | 2.85 | 2.18 | 8.94E-04 |  |  |  | |  | |  |  |  |
| *RPS12* | 8.50 | 18.47 | 2.17 | 3.38E-05 |  |  |  | |  | |  |  |  |
| *BBC3* | 0.35 | 0.77 | 2.17 | 9.39E-03 |  |  |  | |  | |  |  |  |
| *TRIM24* | 0.38 | 0.82 | 2.17 | 4.11E-05 |  |  |  | |  | |  |  |  |
| *EPRS* | 1.45 | 3.15 | 2.17 | 3.35E-05 |  |  |  | |  | |  |  |  |
| *MAD2L2* | 0.10 | 0.21 | 2.17 | 5.95E-03 |  |  |  | |  | |  |  |  |
| *FANCD2* | 0.28 | 0.61 | 2.17 | 2.02E-04 |  |  |  | |  | |  |  |  |
| *STAU1* | 1.79 | 3.88 | 2.17 | 3.36E-05 |  |  |  | |  | |  |  |  |
| *CDK2* | 0.69 | 1.49 | 2.17 | 3.48E-05 |  |  |  | |  | |  |  |  |
| *POLR3K* | 0.20 | 0.43 | 2.17 | 9.38E-03 |  |  |  | |  | |  |  |  |
| *DENR* | 0.81 | 1.75 | 2.17 | 8.07E-05 |  |  |  | |  | |  |  |  |
| *KNOP1* | 0.40 | 0.88 | 2.17 | 8.73E-04 |  |  |  | |  | |  |  |  |
| *EZH2* | 0.55 | 1.18 | 2.17 | 2.58E-04 |  |  |  | |  | |  |  |  |
| *LHFPL5* | 0.30 | 0.64 | 2.16 | 3.87E-02 |  |  |  | |  | |  |  |  |
| *RBM4B* | 0.42 | 0.92 | 2.16 | 4.44E-04 |  |  |  | |  | |  |  |  |
| *NAA10* | 0.29 | 0.63 | 2.16 | 7.55E-04 |  |  |  | |  | |  |  |  |
| *ENOPH1* | 0.65 | 1.40 | 2.16 | 1.46E-04 |  |  |  | |  | |  |  |  |
| *CDK7* | 0.43 | 0.93 | 2.16 | 2.65E-02 |  |  |  | |  | |  |  |  |
| *AKR1E2* | 0.14 | 0.30 | 2.16 | 4.35E-02 |  |  |  | |  | |  |  |  |
| *SPNS2* | 0.81 | 1.75 | 2.16 | 5.81E-04 |  |  |  | |  | |  |  |  |
| *ZBTB9* | 0.16 | 0.35 | 2.16 | 9.62E-04 |  |  |  | |  | |  |  |  |
| *RUVBL1* | 0.45 | 0.98 | 2.15 | 6.32E-05 |  |  |  | |  | |  |  |  |
| *VARS* | 0.61 | 1.31 | 2.15 | 2.04E-03 |  |  |  | |  | |  |  |  |
| *PDZD8* | 1.83 | 3.95 | 2.15 | 3.35E-05 |  |  |  | |  | |  |  |  |
| *ARHGEF19* | 0.16 | 0.35 | 2.15 | 2.08E-02 |  |  |  | |  | |  |  |  |
| *CDC25A* | 0.19 | 0.41 | 2.15 | 4.24E-03 |  |  |  | |  | |  |  |  |
| *UBFD1* | 0.54 | 1.16 | 2.15 | 3.79E-05 |  |  |  | |  | |  |  |  |
| *WDR76* | 0.24 | 0.51 | 2.15 | 1.11E-02 |  |  |  | |  | |  |  |  |
| *POLD1* | 0.40 | 0.85 | 2.15 | 3.51E-02 |  |  |  | |  | |  |  |  |
| *GPX1* | 0.87 | 1.88 | 2.15 | 5.20E-04 |  |  |  | |  | |  |  |  |
| *MARCKSL1* | 3.58 | 7.70 | 2.15 | 3.65E-05 |  |  |  | |  | |  |  |  |
| *PTGES3* | 2.83 | 6.07 | 2.14 | 3.35E-05 |  |  |  | |  | |  |  |  |
| *RPL35* | 3.28 | 7.01 | 2.14 | 2.37E-04 |  |  |  | |  | |  |  |  |
| *SF3B3* | 1.20 | 2.57 | 2.14 | 3.35E-05 |  |  |  | |  | |  |  |  |
| *DNMT1* | 0.95 | 2.04 | 2.14 | 1.42E-04 |  |  |  | |  | |  |  |  |
| *NABP2* | 0.34 | 0.74 | 2.14 | 7.32E-04 |  |  |  | |  | |  |  |  |
| *GPX2* | 8.12 | 17.36 | 2.14 | 5.91E-04 |  |  |  | |  | |  |  |  |
| *C4orf46* | 0.13 | 0.28 | 2.14 | 2.47E-02 |  |  |  | |  | |  |  |  |
| *CALU* | 1.53 | 3.27 | 2.13 | 3.35E-05 |  |  |  | |  | |  |  |  |
| *SMARCC1* | 1.48 | 3.15 | 2.13 | 3.35E-05 |  |  |  | |  | |  |  |  |
| *TXNRD3* | 0.15 | 0.32 | 2.13 | 2.43E-02 |  |  |  | |  | |  |  |  |
| *ILF2* | 1.73 | 3.69 | 2.13 | 5.61E-05 |  |  |  | |  | |  |  |  |
| *PDPN* | 0.25 | 0.54 | 2.13 | 6.83E-03 |  |  |  | |  | |  |  |  |
| *RSL24D1* | 0.82 | 1.75 | 2.13 | 1.38E-04 |  |  |  | |  | |  |  |  |
| *LOX* | 0.17 | 0.35 | 2.13 | 1.51E-04 |  |  |  | |  | |  |  |  |
| *TCEB1* | 0.28 | 0.60 | 2.13 | 7.43E-03 |  |  |  | |  | |  |  |  |
| *TGIF1* | 0.84 | 1.79 | 2.13 | 1.21E-04 |  |  |  | |  | |  |  |  |
| *OCIAD2* | 0.57 | 1.21 | 2.13 | 3.30E-02 |  |  |  | |  | |  |  |  |
| *RHBDF1* | 0.44 | 0.94 | 2.13 | 4.51E-03 |  |  |  | |  | |  |  |  |
| *NONO* | 2.10 | 4.47 | 2.13 | 3.35E-05 |  |  |  | |  | |  |  |  |
| *NUP107* | 0.44 | 0.94 | 2.13 | 6.28E-04 |  |  |  | |  | |  |  |  |
| *TSEN15* | 0.46 | 0.98 | 2.13 | 2.17E-03 |  |  |  | |  | |  |  |  |
| *WDR4* | 0.58 | 1.23 | 2.12 | 4.37E-04 |  |  |  | |  | |  |  |  |
| *ZNF146* | 1.34 | 2.85 | 2.12 | 3.35E-05 |  |  |  | |  | |  |  |  |
| *RPL12* | 2.62 | 5.56 | 2.12 | 3.36E-05 |  |  |  | |  | |  |  |  |
| *RPS5* | 8.77 | 18.62 | 2.12 | 7.74E-05 |  |  |  | |  | |  |  |  |
| *POP1* | 0.15 | 0.32 | 2.12 | 2.89E-03 |  |  |  | |  | |  |  |  |
| *TXLNG* | 0.62 | 1.31 | 2.11 | 1.07E-03 |  |  |  | |  | |  |  |  |
| *ARL6IP1* | 2.83 | 5.98 | 2.11 | 3.35E-05 |  |  |  | |  | |  |  |  |
| *TMSB10* | 30.44 | 64.31 | 2.11 | 3.36E-05 |  |  |  | |  | |  |  |  |
| *RPP25* | 0.33 | 0.70 | 2.11 | 9.42E-04 |  |  |  | |  | |  |  |  |
| *EEF1B2* | 0.67 | 1.43 | 2.11 | 6.87E-04 |  |  |  | |  | |  |  |  |
| *KIF24* | 0.20 | 0.41 | 2.11 | 2.22E-04 |  |  |  | |  | |  |  |  |
| *TRIM59* | 0.35 | 0.74 | 2.11 | 3.58E-05 |  |  |  | |  | |  |  |  |
| *PDIA6* | 1.99 | 4.19 | 2.11 | 3.35E-05 |  |  |  | |  | |  |  |  |
| *LMBR1* | 0.62 | 1.30 | 2.11 | 3.38E-05 |  |  |  | |  | |  |  |  |
| *POLR2D* | 0.69 | 1.44 | 2.10 | 9.97E-05 |  |  |  | |  | |  |  |  |
| *RPS24* | 4.07 | 8.55 | 2.10 | 3.47E-05 |  |  |  | |  | |  |  |  |
| *GNL3* | 1.03 | 2.17 | 2.10 | 9.53E-04 |  |  |  | |  | |  |  |  |
| *RPS2* | 2.20 | 4.61 | 2.10 | 3.56E-05 |  |  |  | |  | |  |  |  |
| *IL17D* | 0.11 | 0.23 | 2.10 | 4.16E-02 |  |  |  | |  | |  |  |  |
| *TXNRD1* | 0.81 | 1.69 | 2.10 | 4.79E-05 |  |  |  | |  | |  |  |  |
| *FKBP4* | 1.18 | 2.48 | 2.09 | 2.86E-03 |  |  |  | |  | |  |  |  |
| *INCENP* | 0.41 | 0.86 | 2.09 | 1.62E-03 |  |  |  | |  | |  |  |  |
| *EPHB2* | 1.42 | 2.97 | 2.09 | 1.52E-02 |  |  |  | |  | |  |  |  |
| *DRAM1* | 0.33 | 0.69 | 2.09 | 1.10E-03 |  |  |  | |  | |  |  |  |
| *SDCCAG3* | 0.56 | 1.17 | 2.09 | 1.99E-03 |  |  |  | |  | |  |  |  |
| *PARD6B* | 0.47 | 0.98 | 2.09 | 1.48E-04 |  |  |  | |  | |  |  |  |
| *BCAP31* | 0.96 | 2.00 | 2.09 | 4.83E-05 |  |  |  | |  | |  |  |  |
| *PES1* | 0.38 | 0.79 | 2.08 | 1.25E-03 |  |  |  | |  | |  |  |  |
| *SLBP* | 0.70 | 1.45 | 2.08 | 1.44E-03 |  |  |  | |  | |  |  |  |
| *PAM* | 0.70 | 1.45 | 2.08 | 7.67E-05 |  |  |  | |  | |  |  |  |
| *CCND2* | 4.54 | 9.42 | 2.07 | 1.88E-04 |  |  |  | |  | |  |  |  |
| *DNTTIP2* | 0.34 | 0.70 | 2.07 | 3.48E-05 |  |  |  | |  | |  |  |  |
| *MRPL51* | 0.74 | 1.53 | 2.07 | 8.42E-05 |  |  |  | |  | |  |  |  |
| *GLB1L2* | 0.25 | 0.52 | 2.07 | 3.52E-03 |  |  |  | |  | |  |  |  |
| *RPL31* | 2.08 | 4.30 | 2.06 | 4.46E-05 |  |  |  | |  | |  |  |  |
| *SSRP1* | 0.76 | 1.57 | 2.06 | 3.51E-05 |  |  |  | |  | |  |  |  |
| *STIL* | 0.32 | 0.67 | 2.06 | 1.59E-04 |  |  |  | |  | |  |  |  |
| *SLC35B2* | 0.73 | 1.51 | 2.06 | 2.13E-04 |  |  |  | |  | |  |  |  |
| *GAS2L3* | 0.39 | 0.81 | 2.06 | 5.62E-03 |  |  |  | |  | |  |  |  |
| *MSH2* | 0.43 | 0.89 | 2.06 | 4.12E-03 |  |  |  | |  | |  |  |  |
| *XPO5* | 0.62 | 1.28 | 2.06 | 3.42E-04 |  |  |  | |  | |  |  |  |
| *SPAG17* | 0.03 | 0.06 | 2.05 | 4.60E-02 |  |  |  | |  | |  |  |  |
| *YWHAQ* | 1.90 | 3.90 | 2.05 | 3.35E-05 |  |  |  | |  | |  |  |  |
| *MXRA5* | 0.70 | 1.44 | 2.05 | 2.60E-04 |  |  |  | |  | |  |  |  |
| *HSPA9* | 1.93 | 3.96 | 2.05 | 3.35E-05 |  |  |  | |  | |  |  |  |
| *PRDX2* | 1.16 | 2.37 | 2.05 | 2.61E-03 |  |  |  | |  | |  |  |  |
| *UBQLN4* | 0.38 | 0.77 | 2.04 | 5.30E-03 |  |  |  | |  | |  |  |  |
| *FBXL16* | 0.18 | 0.37 | 2.04 | 4.83E-02 |  |  |  | |  | |  |  |  |
| *COL4A1* | 2.18 | 4.45 | 2.04 | 3.38E-05 |  |  |  | |  | |  |  |  |
| *NIP7* | 0.49 | 1.00 | 2.03 | 3.20E-02 |  |  |  | |  | |  |  |  |
| *SH3BP4* | 0.74 | 1.49 | 2.03 | 6.22E-05 |  |  |  | |  | |  |  |  |
| *RBBP7* | 0.63 | 1.28 | 2.03 | 1.43E-04 |  |  |  | |  | |  |  |  |
|  |  |  |  |  |  |  |  | |  | |  |  |  |
| *Two class paired* | |  |  |  |  |  |  | |  | |  |  |  |
| *P-value in train and test sets <= 0.05* | | | |  |  |  |  | |  | |  |  |  |
| *Fold change >=2 (<=1/2 for down-regulated); sorted by fold change* | | | | | | | |  | |  | |  |  |
|  |  |  |  |  |  |  |  | |  | |  |  |  |
| *nperms=1000* | |  |  |  |  |  |  | |  | |  |  |  |
| *nresamp=20* | |  |  |  |  |  |  | |  | |  |  |  |
| *random.seed=6352* | | |  |  |  |  |  | |  | |  |  |  |
