# Supplementary material for: Improved survival among colon cancer patients with increased differentially expressed pathways
Source: BMC Med. 2015 Apr 8;13:75. doi: 10.1186/s12916-015-0292-9 (PMC4389992; doi:10.1186/s12916-015-0292-9)
Supplement: Additional file 2: — Major Canonical Pathway and related gene enrichment and key molecules; pathways have a significant gene enrichment after adjustment for multiple comparisons. [file 12916_2015_292_MOESM2_ESM.docx]

| Online Supplement 2. Major Canonical Pathway and related gene enrichment and key molecules; pathways have a significant gene enrichment after adjustment for multiple comparisons. | | | | | | | | |  |
| --- | --- | --- | --- | --- | --- | --- | --- | --- | --- |
| Ingenuity Canonical Pathways | -log(B-H p-value) | Ratio for Enrichment | | Down-  regulated | | Up-regulated | | Molecules | |
| Cell Cycle Control of Chromosomal Replication | 6.47E00 | 5.56E-01 | 0/27 (0%) | | 15/27 (56%) | | *CDK4,CDK7,MCM7,MCM2,CDK2,ORC6,DBF4,ORC1,CDC45,MCM3,MCM6,CDT1,CDK6,MCM4,CDC6* | |  |
| Estrogen-mediated S-phase Entry | 5.44E00 | 5.42E-01 | 1/24 (4%) | | 12/24 (50%) | | *CDK4,CDK2,CDC25A,E2F1,MYC,CCND1,SKP2,CCNE1,RBL1,CCNA2,TFDP1,ESR1,CDK1* | |  |
| Thyroid Hormone Metabolism II (via Conjugation and/or Degradation) | 4.01E00 | 4.19E-01 | 11/31 (35%) | | 2/31 (6%) | | *SULT1C3,UGT1A5,UGT1A4,UGT1A9 (includes others),UGT2B17,SULT1A2,UGT1A1,SULT1B1,DIO2,UGT2B15,UGT2A3,UGT1A3,UGT1A6* | |  |
| Hepatic Fibrosis / Hepatic Stellate Cell Activation | 4.01E00 | 1.98E-01 | 18/197 (9%) | | 21/197 (11%) | | *COL19A1,FGFR2,IL1RL1,COL7A1,PDGFD,IL6R,TNFSF9,COL27A1,EDNRB,COL4A4,COL1A1,IL10RA,MYH3,COL12A1,FN1,MYH11,MET,IL1RAPL1,LTB,COL4A1,COL3A1,TGFB2,COL4A5,MYL6B,COL1A2,COL8A1,CCL21,IGF1,IGF2,CXCL8,COL4A6,CD40LG,COL5A2,NGFR,COL11A1,IL1A,CXCL3,TIMP1,MMP1* | |  |
| Cell Cycle: G1/S Checkpoint Regulation | 3.99E00 | 2.97E-01 | 2/64 (3%) | | 17/64 (27%) | | *HDAC2,CDK4,CCND2,CDK2,CDC25A,E2F1,TGFB2,NRG1,GNL3,MYC,CCND1,SKP2,CCNE1,CDKN2B,PA2G4,RBL1,PAK1IP1,CDK6,TFDP1* | |  |
| Melatonin Degradation I | 3.99E00 | 3.21E-01 | 14/53 (26%) | | 3/53 (6%) | | *SULT1C3,UGT1A5,UGT1A4,UGT1A9 (includes others),UGT2B17,SULT1A2,CYP2C19,UGT1A1,CYP2C9,SULT1B1,CYP19A1,CYP2C18,UGT2B15,UGT2A3,UGT1A3,CYP2S1,UGT1A6* | |  |
| Serotonin Degradation | 3.89E00 | 3E-01 | 15/60 (25%) | | 3/60 (5%) | | *SULT1C3,HSD17B10,ADH1C,DHRS9,UGT1A5,UGT1A4,UGT1A9 (includes others),UGT2B17,SULT1A2,ADH1A,UGT1A1,SULT1B1,ALDH4A1,ADH1B,UGT2B15,UGT2A3,UGT1A3,UGT1A6* | |  |
| Superpathway of Melatonin Degradation | 3.51E00 | 2.93E-01 | 14/58 (24%) | | 3/58 (5%) | | *SULT1C3,UGT1A5,UGT1A4,UGT1A9 (includes others),UGT2B17,SULT1A2,CYP2C19,UGT1A1,CYP2C9,SULT1B1,CYP19A1,CYP2C18,UGT2B15,UGT2A3,UGT1A3,CYP2S1,UGT1A6* | |  |
| Cyclins and Cell Cycle Regulation | 3.4E00 | 2.56E-01 | 2/78 (3%) | | 18/78 (23%) | | *HDAC2,CDK4,CDK7,CCND2,CDK2,CDC25A,E2F1,TGFB2,PPP2R3A,CCNB2,CCND1,SKP2,CCNE1,CDKN2B,PA2G4,CCNA2,CDK6,TFDP1,CDK1,CCNB1* | |  |
| Cell Cycle: G2/M DNA Damage Checkpoint Regulation | 3.31E00 | 3.06E-01 | 0/49 (0%) | | 15/49 (31%) | | *TOP2A,BRCA1,YWHAQ,YWHAG,CDK7,AURKA,CKS2,CCNB2,PKMYT1,SKP2,PRKDC,CDK1,CCNB1,CHEK1,CDC25B* | |  |
| GADD45 Signaling | 3.31E00 | 4.74E-01 | 0/19 (0%) | | 9/19 (47%) | | *BRCA1,CDK4,CCND2,CCNE1,CCND1,CDK2,PCNA,CDK1,CCNB1* | |  |
| Mismatch Repair in Eukaryotes | 3.06E00 | 5E-01 | 0/16 (0%) | | 8/16 (50%) | | *FEN1,RFC3,EXO1,RFC2,POLD1,RFC4,PCNA,MSH2* | |  |
| Mitotic Roles of Polo-Like Kinase | 2.94E00 | 2.58E-01 | 1/66 (2%) | | 16/66 (24%) | | *KIF23,PLK5,ESPL1,CDC25A,PRC1,CDC20,PPP2R3A,CCNB2,KIF11,PKMYT1,PLK4,HSP90AB1,PTTG1,HSP90AA1,CDK1,CCNB1,CDC25B* | |  |
| Agranulocyte Adhesion and Diapedesis | 2.94E00 | 1.8E-01 | 12/189 (6%) | | 22/189 (12%) | | *CXCL12,CCL8,ITGA2,CLDN8,PODXL,CLDN1,PODXL2,CLDN2,CXCL11,MYH3,FN1,IL1RN,MYH11,CXCL1,CLDN5,ACTG2,CCL28,MMP11,CXCL10,CCL24,CCL13,CLDN12,MYL6B,CCL21,CXCL2,CXCL8,MMP7,CCL19,MMP3,IL1A,CLDN23,CXCL3,MMP1,MMP14* | |  |
| Hereditary Breast Cancer Signaling | 2.87E00 | 2.09E-01 | 0/115 (0%) | | 24/115 (21%) | | *BRCA1,RFC3,HDAC2,CDK4,FANCB,TUBG1,UBD,E2F1,H2AFX,BLM,FANCD2,POLR2D,CCND1,RFC2,FANCG,BRCA2,POLR2I,CDK6,RFC4,NPM1,CDK1,CCNB1,MSH2,CHEK1* | |  |
| Granulocyte Adhesion and Diapedesis | 2.81E00 | 1.81E-01 | 13/177 (7%) | | 19/177 (11%) | | *IL1RL1,CXCL12,ITGAL,CCL8,ITGA2,CLDN8,THY1,CLDN1,CLDN2,CXCL11,IL1RN,CXCL1,CLDN5,IL1RAPL1,CCL28,MMP11,CXCL10,CCL24,CCL13,CLDN12,CCL21,CXCL2,CXCL8,MMP7,CCL19,MMP3,NGFR,IL1A,CLDN23,CXCL3,MMP1,MMP14* | |  |
| Nicotine Degradation III | 2.76E00 | 2.8E-01 | 12/50 (24%) | | 2/50 (4%) | | *UGT1A5,UGT1A4,UGT1A9 (includes others),UGT2B17,CYP2C19,UGT1A1,CYP2C9,CYP19A1,CYP2C18,UGT2B15,UGT2A3,UGT1A3,CYP2S1,UGT1A6* | |  |
| Nicotine Degradation II | 2.52E00 | 2.54E-01 | 13/59 (22%) | | 2/59 (3%) | | *UGT1A5,UGT1A4,UGT1A9 (includes others),UGT2B17,CYP2C19,UGT1A1,CYP2C9,FMO5,CYP19A1,CYP2C18,UGT2B15,UGT2A3,UGT1A3,CYP2S1,UGT1A6* | |  |
| p53 Signaling | 2.21E00 | 2.04E-01 | 0/98 (0%) | | 20/98 (20%) | | *SERPINB5,BRCA1,CDK4,BBC3,CCND2,BIRC5,SERPINE2,CDK2,E2F1,PCNA,GNL3,PERP,DRAM1,TRIM29,CCND1,BCL2L1,PRKDC,PMAIP1,C12orf5,CHEK1* | |  |
| Axonal Guidance Signaling | 2.09E00 | 1.36E-01 | 33/433 (8%) | | 26/433 (6%) | | *EFNA4,BMP5,TUBB3,GNG7,TUBG1,SLIT3,PLCE1,ADAMTS8,TUBA1C,BMP2,PRKACB,ADAMTS2,WNT2B,BMP4,GNG4,PRKCB,ADAM12,EFNA3,UNC5D,MMP11,EPHB3,SEMA6A,MYL6B,EPHB2,SEMA3D,IGF1,ADAMTS1,HHIP,PLXNA1,TUBA1B,SHH,ADAM28,BMP3,CXCL12,PDGFD,GNAO1,ITGA2,SEMA6D,ADAM29,PTPN11,WNT2,MET,PSMD14,NTRK3,BMP6,FZD3,PAPPA2,BMP7,EPHA10,PLCD1,TUBB,EPHB4,WNT5B,EPHA7,PLCG2,MMP7,UNC5C,NGFR,PLCL2* | |  |
| Aryl Hydrocarbon Receptor Signaling | 2.04E00 | 1.79E-01 | 3/140 (2%) | | 22/140 (16%) | | *POLA1,MCM7,E2F1,NQO1,FOS,CCNE1,GSTM2,RBL1,CDK6,GSTP1,HSP90AA1,ESR1,CDK4,CCND2,CDK2,TGFB2,ALDH4A1,MYC,CCND1,PTGES3,IL1A,CCNA2,HSP90AB1,TFDP1,CHEK1* | |  |
| Role of BRCA1 in DNA Damage Response | 1.67E00 | 2.19E-01 | 0/64 (0%) | | 14/64 (22%) | | *BRCA1,RFC3,FANCB,E2F1,BRIP1,BLM,FANCD2,RFC2,FANCG,RBL1,BRCA2,RFC4,MSH2,CHEK1* | |  |
| ATM Signaling | 1.54E00 | 2.2E-01 | 1/59 (2%) | | 12/59 (20%) | | *BRCA1,MAPK10,CDK2,CDC25A,CCNB2,SMC2,H2AFX,BLM,TRIM28,FANCD2,CDK1,CCNB1,CHEK1* | |  |
| Complement System | 1.51E00 | 2.73E-01 | 6/33 (18%) | | 3/33 (9%) | | *MASP1,CFB,C2,CR2,CD46,C7,CFD,CR1,CFH* | |  |
| Superpathway of Serine and Glycine Biosynthesis I | 1.51E00 | 5.71E-01 | 0/7 (0%) | | 4/7 (57%) | | *PSAT1,PSPH,PHGDH,SHMT2* | |  |
| Atherosclerosis Signaling | 1.43E00 | 1.71E-01 | 9/123 (7%) | | 12/123 (10%) | | *CXCL12,CCR2,PDGFD,PLA2G16,PLA2G10,COL3A1,COL1A2,TPSAB1/TPSB2,COL1A1,CXCL8,LYZ,MMP3,CD36,CD40LG,IL1RN,PNPLA3,IL1A,TNFRSF12A,ALOX12B,MMP1,PLA2G2A* | |  |
| Role of CHK Proteins in Cell Cycle Checkpoint Control | 1.41E00 | 2.18E-01 | 1/55 (2%) | | 11/55 (20%) | | *BRCA1,RFC3,RFC2,CDK2,CDC25A,E2F1,CLSPN,PPP2R3A,RFC4,PCNA,CDK1,CHEK1* | |  |
| RAN Signaling | 1.41E00 | 3.53E-01 | 0/17 (0%) | | 6/17 (35%) | | *CSE1L,KPNA2,RCC1,IPO5,RAN,RANBP1* | |  |
| Purine Nucleotides De Novo Biosynthesis II | 1.41E00 | 4.17E-01 | 0/12 (0%) | | 5/12 (42%) | | *IMPDH1,PAICS,PPAT,GMPS,ATIC* | |  |
| Eicosanoid Signaling | 1.38E00 | 2.06E-01 | 9/63 (14%) | | 4/63 (6%) | | *PLA2G16,PLA2G10,DPEP2,DPEP1,HPGDS,PTGER2,PTGDS,PNPLA3,CYSLTR1,ALOX12B,PTGES,PTGDR,PLA2G2A* | |  |
| Wnt/β-catenin Signaling | 1.34E00 | 1.54E-01 | 8/169 (5%) | | 18/169 (11%) | | *GNAO1,DKK2,UBD,CSNK2A2,SFRP1,PPP2R3A,NR5A2,WNT2B,AXIN2,WNT2,FZD3,LEF1,MAP4K1,WNT5B,TGFB2,CDH3,CD44,MMP7,SOX10,RUVBL2,MYC,SFRP4,SOX11,CCND1,SOX9,SOX4* | |  |
|  |  |  |  | |  | |  | |  |
