# Supplementary material for: Improved survival among colon cancer patients with increased differentially expressed pathways
Source: BMC Med. 2015 Apr 8;13:75. doi: 10.1186/s12916-015-0292-9 (PMC4389992; doi:10.1186/s12916-015-0292-9)
Supplement: Additional file 3: Figure S1. — Kaplan-Meier curve for de-regulated genes in the Thyroid Hormone Metabolism II in the IPA Canonical Pathway. [file 12916_2015_292_MOESM3_ESM.pdf]

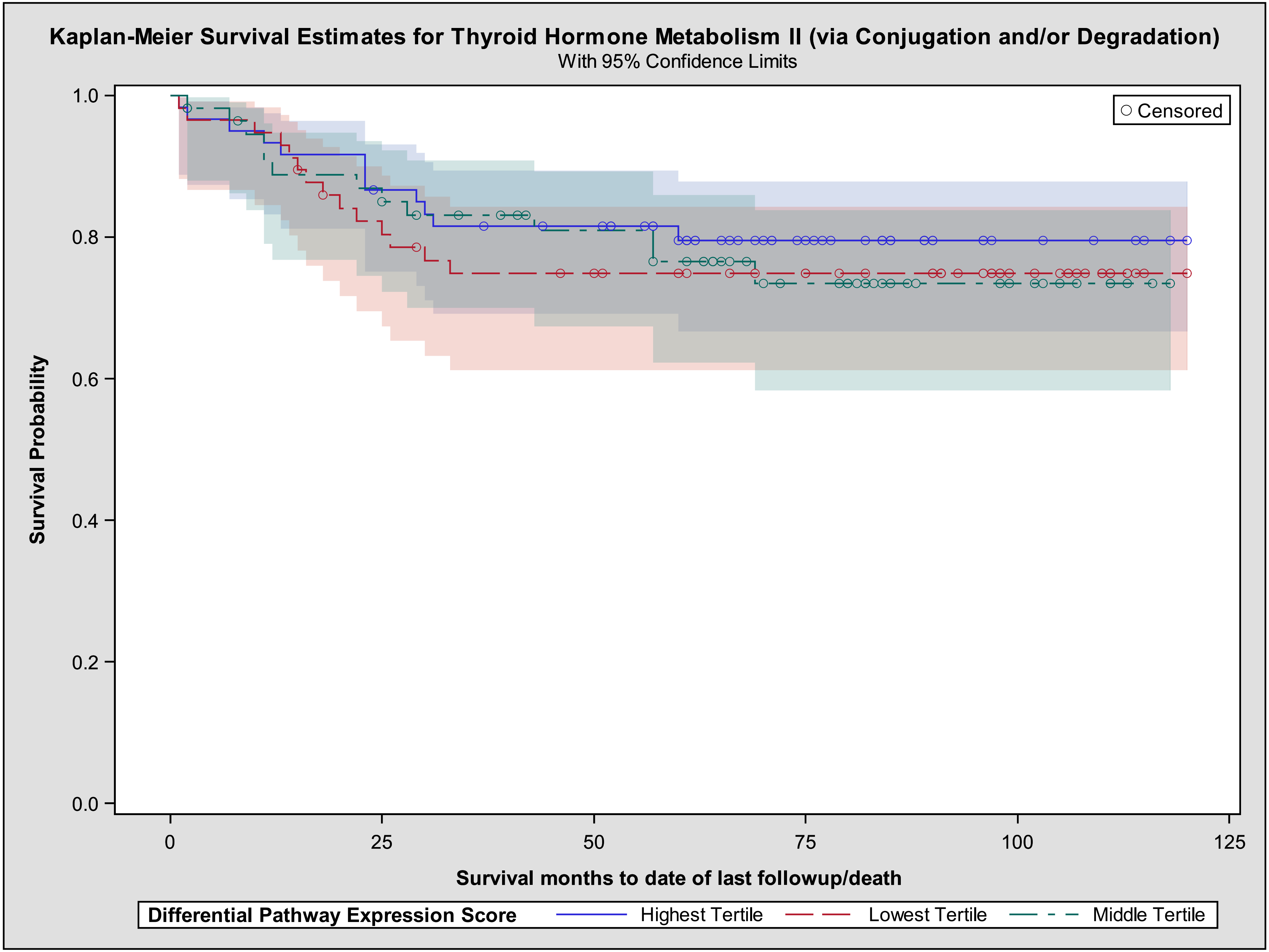

Kaplan-Meier Survival Estimates for Thyroid Hormone Metabolism II (via Conjugation and/or Degradation)
With 95% Confidence Limits
Censored
Survival Probability
1.0
0.8
0.6
0.4
0.2
0.0
0
25
50
75
100
125
Survival months to date of last followup/death
Differential Pathway Expression Score
Highest Tertile
Lowest Tertile
Middle Tertile
