# Supplementary material for: Improved survival among colon cancer patients with increased differentially expressed pathways
Source: BMC Med. 2015 Apr 8;13:75. doi: 10.1186/s12916-015-0292-9 (PMC4389992; doi:10.1186/s12916-015-0292-9)
Supplement: Additional file 4: Figure S2. — Kaplan-Meier curve for de-regulated genes in the Cell Cycle Control of Chromosomal Replication Pathway IPA Canonical Pathway. [file 12916_2015_292_MOESM4_ESM.pdf]

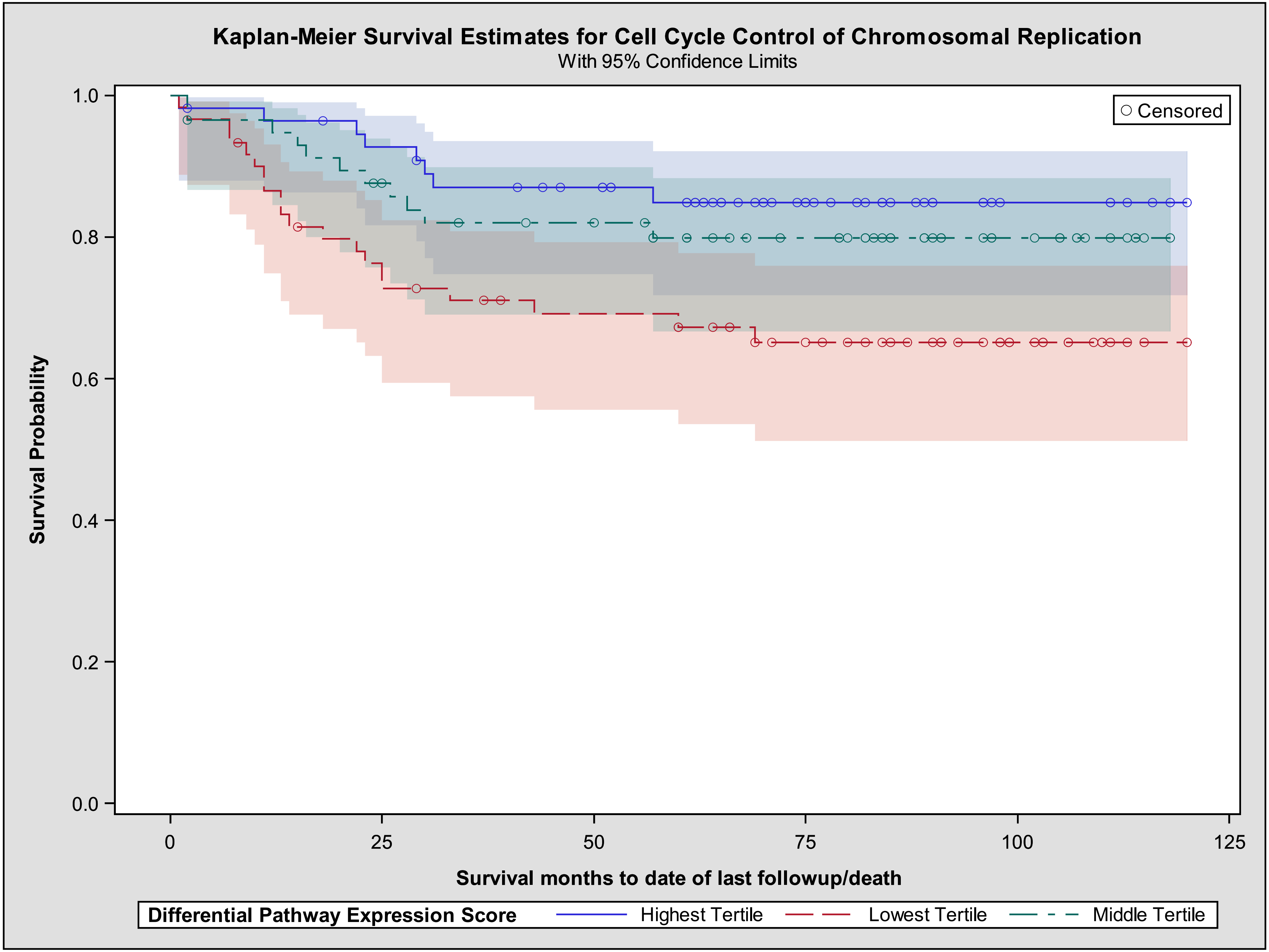
Kaplan-Meier Survival Estimates for Cell Cycle Control of Chromosomal Replication
With 95% Confidence Limits
Censored
Survival Probability
1.0
0.8
0.6
0.4
0.2
0.0
0
25
50
75
100
125
Survival months to date of last followup/death
Differential Pathway Expression Score
Highest Tertile
Lowest Tertile
Middle Tertile
