# Supplementary figures and images for: Improved survival among colon cancer patients with increased differentially expressed pathways
Source: BMC Med. 2015 Apr 8;13:75. doi: 10.1186/s12916-015-0292-9 (PMC4389992; doi:10.1186/s12916-015-0292-9)

# Kaplan-Meier Survival Estimates for Estrogen-mediated S-phase Entry

With 95% Confidence Limits

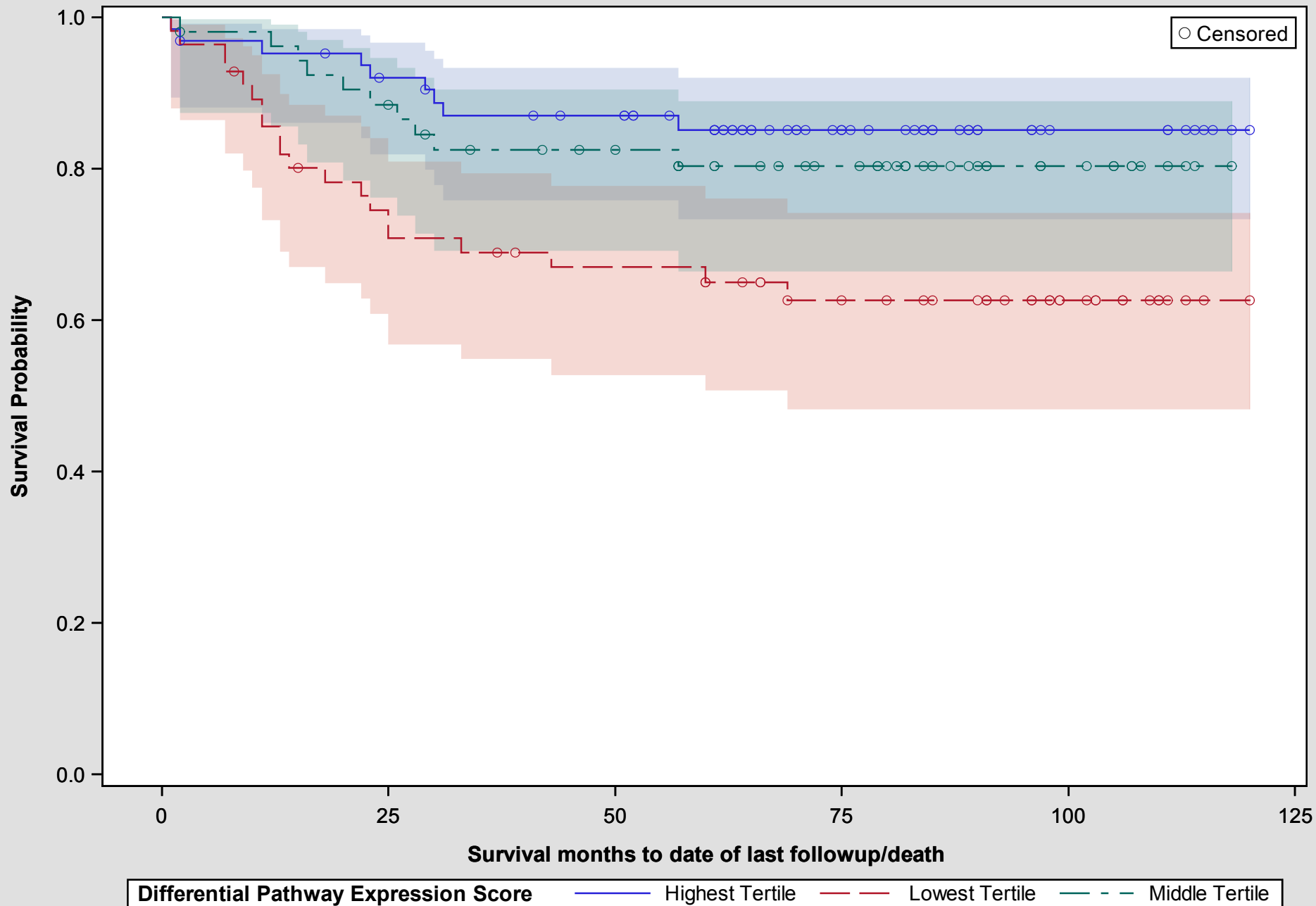

Supplement: Additional file 5: Figure S3. — Kaplan-Meier curve for de-regulated genes in the Estrogen-mediated S-phase Entry IPA Canonical Pathway. [file 12916_2015_292_MOESM5_ESM.pdf]
