# Supplementary material for: Improved survival among colon cancer patients with increased differentially expressed pathways
Source: BMC Med. 2015 Apr 8;13:75. doi: 10.1186/s12916-015-0292-9 (PMC4389992; doi:10.1186/s12916-015-0292-9)
Supplement: Additional file 6: Table S3. — Description of major upstream regulators and their targets in our data. [file 12916_2015_292_MOESM6_ESM.docx]

| Online Supplemental Table 3. Description of major upstream regulators and their targets in our data. | | | | | | | |  |  |
| --- | --- | --- | --- | --- | --- | --- | --- | --- | --- |
|  | Molecule Type | Predicted Activation State | | p-value of overlap | | Target molecules in dataset | Mechanistic Network | | |
| TGFB1 | growth factor | Activated | 8.14E-46 | | ABCE1,ABCG2,ACAN,ACKR1,ACKR2,ACTG2,ACVRL1,ADAM12,ADAMTS12,ADAMTS2,AK1,ANPEP,APLN,AQP8,ARHGEF19,ASPM,ASUN,BBC3,BCL2L1,BGN,BHLHE40,BIRC5,BMP2,BMP4,BMP6,BMP7,BRIP1,BUB1,BUB1B,C2,C20orf24,CBFB,CCDC85B,CCNA2,CCNB1,CCNB2,CCND1,CCND2,CCNE1,CCR2,CCT2,CCT5,CCT6A,CD36,CD44,CD46,CD69,CDC20,CDC25A,CDH11,CDH19,CDK1,CDK2,CDK4,CDKN2B,CDKN3,CDT1,CEMIP,CENPA,CENPE,CENPF,CHI3L1,CITED2,CKS2,CNN1,CNN2,COL11A1,COL1A1,COL1A2,COL3A1,COL4A1,COL4A6,COL7A1,COL8A1,COMP,CTPS1,CXCL1,CXCL10,CXCL12,CXCL2,CXCL3,CXCL8,DDIT4,DDX21,DES,DISP2,DKC1,DNAH2,DNAJA1,DNMT1,DNMT3B,DOCK2,DUSP1,DUSP4,E2F1,EDNRB,EIF4A3,EPHB2,EPRS,EREG,ESM1,ESPL1,F13A1,FAM107A,FCGR3A/FCGR3B,FERMT1,FGF9,FGFR2,FHL1,FLI1,FN1,FOS,FOSB,FSCN1,FXYD5,GAD1,GAL,GARS,GAS1,GDF15,GDPD5,GNAO1,GNG7,GPT,GRHL1,HAPLN3,HDAC2,HDGF,HMGA1,HOXD1,HS6ST2,HSD17B10,HSD3B2,HSP90AA1,HTRA1,IARS,IGF1,IGF2,IL10RA,IL11,IL17D,IL1A,IL1RL1,IL1RN,IL6R,IMPDH1,INHBA,IRAK2,ITGA11,ITGA2,ITGAL,ITGBL1,JAG2,KIF3C,KLF4,KLF9,KLK3,KLRB1,KRT17,KRT18,KRT7,LAMC2,LBR,LDHA,LIF,LIFR,LOX,LPAR1,LYVE1,MCM2,MEF2C,MET,MFAP2,MFAP4,MFI2,MGAT5,MIA2,MKI67,MLXIPL,MMP1,MMP11,MMP14,MMP3,MMP7,MPP6,MPZ,MS4A2,MS4A8,MTHFD2,MUC4,MYBL2,MYC,MYH11,MYOCD,NCAM1,NCAPD2,NCAPG,NDC80,NEK2,NME1,NOP58,NOTCH3,NOX4,OLR1,ORC1,OSM,P2RY1,P2RY14,P4HA1,PA2G4,PCNA,PDPN,PGK1,PHGDH,PHLDA2,PLAGL2,PLAU,PLEK2,PLS3,PMEPA1,PNOC,PODXL,POLD1,POLE2,PRC1,PRDX4,PRSS22,PSPH,PTGDS,PTGER2,PTGES,RAB31,RACGAP1,RAD51AP1,RBL1,RBMS3,RFC4,RNF152,RPN2,RUNX1,S100A11,S100A6,SCD,SDPR,SELENBP1,SERPINA3,SERPINB5,SERPINE2,SERPINH1,SFRP1,SLC13A3,SLC16A9,SLC2A1,SLC4A2,SLC51B,SLC7A1,SLC7A5,SLIT3,SMC2,SMC4,SNAI1,SOX4,SOX9,SPARC,SPARCL1,SPOCK1,SPP1,SRI,SRM,SSRP1,STC2,STRAP,TENM4,TGFB2,TGFBI,TGIF1,THY1,TIMP1,TLL1,TMIGD1,TNFRSF12A,TOP2A,TPSAB1/TPSB2,TRIM9,TSC22D3,TSPAN7,TUBB3,TWIST1,TXNRD1,VAT1L,VCAN,VIPR1,WISP1,WNT5B,XDH,ZWINT | | | 743 (20) |  |
| beta-estradiol | chemical - endogenous mammalian | Activated | 1.21E-41 | | ABCB11,ABCG2,ACKR2,ACSL1,ACTL6A,ADAMDEC1,ADH1C,AGRN,ALDH4A1,ALK,ANPEP,ANXA3,APCDD1,ASB2,ASB9,ATAD2,ATP6V1F,AURKA,BCAS1,BCL2L1,BHLHE40,BIRC5,BMP2,BMP4,BMP5,BRCA1,BRCA2,BUB1,CA12,CA2,CACNA1G,CAD,CAPN13,CCL21,CCNA2,CCNB1,CCNB2,CCND1,CCND2,CCNE1,CCR2,CCT2,CD276,CD44,CD69,CDC123,CDC20,CDC25A,CDC45,CDC6,CDCA7,CDH11,CDK1,CDK2,CENPM,CFB,CHEK1,CHGA,CHGB,CHI3L1,CITED2,CLCA1,CLDN8,CNN1,CNN2,CNR1,COL1A1,COL1A2,COL3A1,COL4A5,COMP,CSE1L,CSTB,CTPS1,CXCL10,CXCL11,CXCL12,CXCL2,CXCL3,CXCL8,CYP19A1,DBN1,DCLK1,DDIT4,DHCR7,DHRS9,DUSP1,DUSP4,E2F1,EIF3B,ENC1,ENO1,ENPP2,ESR1,ETV4,FADS1,FASN,FEN1,FGF9,FGFR2,FMO5,FN1,FOS,FOSL1,FUCA1,FXYD5,GAL,GARS,GHR,GINS3,GNL3,GPX1,GRB10,GSTP1,GTF2IRD1,HBB,HCRTR1,HDAC2,HDGF,HELLS,HES6,HSD11B2,HSD17B2,HSP90AB1,HSPA8,HSPD1,HSPH1,HTR4,IARS,IGF1,IGF2,IL17RD,IL1A,IL24,IL6R,IL7R,INHBA,IPO4,IRF4,ITGA2,ITGBL1,KLF4,KLF9,KLK3,KLK6,KPNA2,KRT17,KRT18,KRT6B,KRT7,LDB3,LDHA,LRP8,LTB,LUM,LYZ,LZTS3,MAL,MCM2,MCM3,MCM6,MCM7,MET,MGLL,MKI67,MME,MMP1,MMP7,MMRN1,MPEG1,MPZ,MYBL2,MYC,MYH3,NCAM1,NME1,NOP56,NOP58,NOTCH3,NPM1,NPR3,NPY1R,NR3C2,NR5A2,NSDHL,NTRK3,ORC1,OXTR,PA2G4,PAM,PAPSS2,PARD6B,PCNA,PDCD4,PDK4,PES1,PGK1,PHLDA1,PIGR,PKIB,PKM,PKMYT1,PLA2G10,PLAU,PLIN2,PMAIP1,POLA1,POLE2,PPL,PRC1,PRKCB,PSMA2,PTGDS,PTGER2,PTGES,PTPN11,PTPN13,PTPRU,PTTG1,RAB31,RAN,RANBP1,RASGRP2,RBBP7,RCN2,RFC4,RPN2,RRM2,RUNX1,S100A6,SELENBP1,SERPINA3,SERPINB5,SET,SFRP1,SFRP4,SHH,SKP2,SLC12A2,SLC13A3,SLC2A1,SLC35D3,SLC39A6,SLC3A2,SLC6A20,SLC7A5,SMC2,SMPDL3A,SNAI1,SOX9,SPARC,SPOCK1,SQLE,SRD5A1,SSBP2,SST,SSTR5,STC2,STXBP1,SULT1C2,SYNPO,TACSTD2,TARS,TCEB1,TFAP2C,TGFB2,TGIF2,TK1,TM4SF1,TMEM37,TOP2A,TPD52L1,TRAIP,TRIM24,TSC22D3,TSPAN5,TTC9,TUBG1,TWIST1,TXN,TXNRD1,UGT1A1,VCAN,VIPR1,WNT5B,ZBTB9 | | | 779 (24) |  |
| TP53 | transcription regulator | Inhibited | 1.90E-38 | | AHCY,AK1,ALDH4A1,ANLN,ANTXR1,ANXA3,ARL6IP1,ASF1B,ASPM,ATAD2,AURKA,AURKB,AXIN2,BAI3,BBC3,BCAP31,BCL2L1,BHLHE40,BIRC5,BRCA1,BRCA2,BUB1,BUB1B,C12orf5,C2,CA9,CALU,CCNA2,CCNB1,CCNB1IP1,CCNB2,CCND1,CCND2,CCNE1,CD44,CDC20,CDC25A,CDC25B,CDC6,CDH3,CDK1,CDK2,CDK4,CDK7,CDKN3,CDT1,CENPF,CEP55,CGREF1,CHEK1,CKAP2,CNN1,CNN2,COL1A1,COL1A2,COL3A1,COL4A1,COL5A2,CSTB,CSTF1,CXCL1,CXCL12,CXCL8,DBF4,DDIAS,DDIT4,DHCR7,DHRS9,DLGAP5,DNMT1,DNTTIP1,DPEP1,DRAM1,DSN1,DUSP1,DUSP4,E2F1,EDIL3,ENPP2,ESPL1,ESR1,EXO1,EZH2,FAM83D,FANCI,FASN,FEN1,FHL1,FIGNL1,FKBP4,FN1,FOS,FOSL1,FOXM1,FUCA1,GAPDH,GAS1,GDF15,GJB3,GNL3,GPX1,GSTP1,GTSE1,H2AFX,H2AFZ,HDAC2,HDC,HJURP,HMGB2,HMMR,HSP90AA1,HSP90AB1,HSPA8,HSPD1,HSPH1,IGF1,IGF2,IL10RA,IL16,IL1A,INHBA,IPO7,ITGA2,KIAA0101,KIF23,KIF24,KIFC1,KLF4,KLK3,KPNA2,KRT18,LAMA5,LBR,LDHA,LIF,LSP1,LYZ,MAD2L1,MAP4K1,MCM2,MCM3,MCM4,MCM6,MCM7,ME1,MELK,MET,MFAP2,MIS18A,MKI67,MMP1,MMP3,MSH2,MYBL2,MYC,NCAPG,NCAPH,NDC80,NEK2,NKD1,NME1,NOX4,NPM1,NUSAP1,OTX1,P4HA1,PADI2,PARD6B,PBK,PCK1,PCNA,PDE6A,PDIA6,PDRG1,PERP,PHLDA1,PLA2G16,PLAU,PMAIP1,PMEPA1,PODXL,POLA1,POLD1,POLD2,POLE2,PRC1,PRDX2,PRKCB,PSRC1,PTGDS,PTP4A3,PTPN11,PTPRU,PTTG1,RACGAP1,RAD51AP1,RAD54B,RBBP7,RBL1,RFC3,RFC4,RGS16,RPN2,RRM1,RRM2,RTKN,RUNX1,RUVBL2,SEMA6A,SERPINA3,SERPINB5,SERPINE2,SERPINH1,SH3BP4,SLC2A1,SLC2A12,SLC6A6,SMC2,SMC4,SNAI1,SPP1,SQLE,STAU1,STMN1,TFDP1,TGFB2,TGFBI,THBS2,THY1,TMEM97,TMSB10/TMSB4X,TNFSF9,TOP2A,TPD52L1,TPX2,TRAP1,TRIB3,TRIM28,TSC22D3,TTK,TUBB3,TWIST1,UBE2C,UBE2T,VCAN,VRK1,WDHD1,ZAP70 | | | 616 (20) |  |
| CDKN1A | kinase | Inhibited | 1.41E-37 | | ANLN,ARL6IP1,ASPM,ATAD2,AURKA,AURKB,BBC3,BIRC5,BRCA1,BUB1,BUB1B,CCNA2,CCNB1,CCND1,CCNE1,CDC20,CDC25A,CDC25B,CDC6,CDK1,CDK4,CDKN2B,CDKN3,CENPF,CEP55,CHAF1B,CHEK1,CIT,CPA3,DLGAP5,DTL,DUSP1,EXO1,FANCG,FANCI,FN1,FOXM1,H2AFX,H2AFZ,HJURP,HMGB2,INHBA,KIAA0101,KIF20A,KIF2C,KNSTRN,KRT18,LBR,MAD2L1,MCM2,MCM3,MCM4,MCM6,MCM7,MKI67,MMP1,MMP3,MYBL2,MYC,NPM1,NUSAP1,ORC1,P4HA1,PBK,PCNA,PHGDH,POLD1,PRC1,RACGAP1,RFC4,RRM1,SKP2,SMC2,SMC4,SOX9,SPAG5,STMN1,TOP2A,TPX2,TTK,TUBB3,UBE2C,UBE2S,UBE2T,WDHD1 | | | 485 (14) |  |
| MYC | transcription regulator | Activated | 4.59E-36 | | ABCE1,ACAN,ADAMTS1,AHCY,ALCAM,APCDD1,ARHGAP25,ARL6IP1,AURKB,BBC3,BCL2L1,BIRC5,BRCA1,BUB1,BUB1B,C1QBP,CAD,CCNA2,CCNB1,CCNB2,CCND1,CCND2,CCNE1,CCT3,CD44,CD48,CD69,CDC20,CDC25A,CDC25B,CDCA7,CDK1,CDK2,CDK4,CDK6,CDK7,CDKN2B,CHEK1,CKS2,COL1A1,COL1A2,COL3A1,COL4A1,COL5A2,COL8A1,CSTB,CXCL10,CXCL8,DBN1,DDIT4,DDX21,DKC1,DNMT1,DUSP1,DUSP4,E2F1,EIF2S2,EIF4EBP1,ENO1,EVX1,EZH2,FABP1,FABP2,FAP,FASN,FBL,FN1,FOS,FOSL1,FOXM1,GAPDH,GAS1,GGH,GPT,GRHL3,GTF2F2,H2AFZ,HAPLN1,HDAC2,HMGA1,HSD11B2,HSP90AA1,HSPA9,HSPD1,HSPE1,HSPH1,IGLL1/IGLL5,INHBA,IPO7,ITGAL,KLF4,KLK6,KRT17,KRT6B,KRT7,LDHA,LDHB,LOX,LUM,LYZ,MAD2L1,MAP4K1,MCM6,MCM7,MIF,MKI67,MMP7,MSH2,MSX2,MTBP,MTHFD1,MYC,NAP1L1,NCAM1,NCL,NME1,NME2,NOLC1,NOP56,NOP58,NPM1,PAICS,PAM,PCK1,PCNA,PDCD4,PERP,PGK1,PKM,PLAU,PLP1,PLS3,PMAIP1,POLD1,POLR1B,POLR2D,PPAT,PPL,PRDX2,PRDX4,PRKACB,PRMT1,PSAT1,PYCR1,RANBP1,RBBP7,RCC1,RHPN1,RPL23,RPL26,RPL27,RPL35,RPS12,RRM2,RRP1B,RRS1,RUVBL1,RUVBL2,S100A6,SERPINE2,SERPINH1,SFRP1,SHMT2,SKP2,SLC11A1,SLC2A1,SLC3A2,SLC7A5,SMS,SNRPD2,SOX9,SPARC,SPP1,SRM,STMN1,TAT,TDGF1,TESPA1,TFDP1,TGFB2,THBS2,THY1,TIMP1,TKT,TMEM97,TMSB10/TMSB4X,TRAP1,TSPAN7,TXN,TXNRD1,UBE2C,UBE2S,UGT1A1,UGT1A3,UGT1A6,VARS,WISP1,ZIC2 | | | 660 (22) |  |
| calcitriol | chemical drug | Inhibited | 4.51E-34 | | ALPI,ANLN,BCL2L1,BMP7,BRCA1,BUB1B,CA2,CCNA2,CCNB1,CCND1,CCNE1,CDC20,CDC45,CDC6,CDCA5,CDK1,CDT1,CELSR3,CENPA,CENPH,CENPM,CENPQ,CFD,CHAF1B,CHGA,CHTF18,COL4A1,CSE1L,CXCL10,CXCL2,CXCL3,CXCL8,CYP19A1,CYP2C9,DUSP1,DUSP10,ECT2,EPHB2,ESPL1,ESR1,ETV5,EXO1,EZH2,FAM107A,FASN,FCER2,FEN1,FIGNL1,FN1,FOS,FOXM1,GAPDH,GDPD5,GHR,H2AFZ,HSD17B2,HSPD1,IFITM1,IGF1,IL10RA,IL1A,IL1RN,INCENP,IRF4,KCNH1,KIAA0101,KIF20A,KIF23,KLF4,KLK10,KLK6,KLK7,KPNA2,KRT23,LPAR1,MAD2L1,MAL,MCM2,MCM3,MCM4,MCM7,MELK,MMP1,MMS22L,MYC,NCAM1,NCAPD2,NEK2,NOX4,NUP37,NUSAP1,OSTN,PADI2,PBK,PCNA,PDCD4,PDE9A,PMEPA1,POLE2,PRC1,PTGER2,RACGAP1,RAD51AP1,RFC3,RRM2,SKP2,SMC2,SPAG5,SPP1,STMN1,SULF1,SULT1C2,TACC3,TGFB2,TIMM50,TK1,TLR7,TMEM100,TPX2,TXN | | | 538 (24) |  |
| E2F4 | transcription regulator | | 9.49E-34 | | ANLN,ASF1B,AURKB,BRCA1,CCNA2,CCND1,CCNE1,CDC25A,CDC6,CDCA4,CDK1,CDK2,CENPA,CENPE,CHEK1,CKS2,CSTF1,DBF4,E2F1,ECT2,FAM60A,FANCD2,FEN1,FOXM1,GINS1,H2AFX,H2AFZ,HIST1H3D,HMMR,KIAA0101,KIF4A,LPAR1,MAD2L1,MCM10,MCM3,MCM4,MCM6,MKI67,MSH2,MT1G,MTHFD1,MYBL2,MYC,NCL,NDC80,NEK2,NPM1,OIP5,ORC1,PCNA,POLA1,POLD1,PRC1,PRKDC,PSAT1,PTTG1,RAD51AP1,RAD54L,RANBP1,RBL1,RFC2,RFC3,RFC4,RRM1,RRM2,SFRP1,SHH,SLC3A2,SMC2,SMC4,SNRPC,SSX2IP,STMN1,TEAD4,TK1,TOP2A,TPX2,TTK,UBE2C,UBE2T,WDR76 | | | 250 (4) |  |
| dexamethasone | chemical drug | Inhibited | 1.16E-31 | | ABCC8,ABHD2,ACADS,ACSL1,ACTG2,ADAM12,ADAMTS1,ADAMTS2,ALCAM,ALDH4A1,ALDOB,AMIGO2,ANK2,ANO1,APLN,ARHGEF19,ASPH,ATP11A,AXIN2,BBC3,BCL2L1,BGN,BHLHE40,BIRC5,BMP2,BMP5,BRCA1,BRCA2,BZW2,C12orf5,CA2,CCL21,CCNA2,CCND1,CCND2,CCNE1,CCR2,CD209,CD36,CD3E,CD44,CD69,CDK2,CDK4,CDKN2B,CEACAM6,CEACAM7,CFB,CFD,CHGA,CHI3L1,CLDN5,CLIC1,COL11A1,COL1A1,COL4A1,COL4A5,COL7A1,CPSF3,CR2,CTNNAL1,CXCL1,CXCL10,CXCL12,CXCL2,CXCL3,CXCL8,CYP19A1,CYP2C19,CYP2C9,CYP2S1,DDIT4,DIO2,DLGAP5,DUSP1,DUSP10,DUSP4,ECT2,EDNRB,EIF1AX,ENC1,EREG,ETV5,EYA2,FAM107A,FASN,FKBP4,FN1,FOS,FOSB,FOSL1,GAL,GAPDH,GAS1,GDF15,GHR,GPR115,GPT,GPX1,GSTP1,HAPLN1,HDAC2,HMGA1,HMMR,HPGDS,HSD11B2,HSPA8,HSPH1,IFITM1,IFITM3,IGF1,IGF2,IL10RA,IL11,IL16,IL1A,IL1RL1,IL1RN,IL6R,IL7R,ILF2,INHBA,IRAK1,ITGA2,ITGBL1,KAL1,KIF14,KIF4A,KIFC1,KLF9,KPNA2,KRT17,KRT7,LAMA5,LDHA,LIF,LPAR1,LRP8,LSP1,LTB,LY6E,LYVE1,MCM4,ME1,METTL7A,MFAP5,MID1IP1,MIF,MME,MMP1,MMP14,MMP3,MS4A1,MSX2,MT1G,MT1H,MT1M,MT1X,MTHFD1,MUSK,MXRA5,MYC,NCAM1,NCAPH,NEBL,NGFR,NPM1,NPR3,NUF2,OLFML2B,OLR1,OSM,OXTR,P4HA1,PADI2,PAM,PCK1,PCSK2,PCSK5,PDCD4,PDK4,PDX1,PER1,PHLDA1,PHLDA2,PIM2,PLA2G16,PLAU,PLCL2,PLIN1,PMCH,PODXL,PPA1,PPARGC1A,PRKG2,PSMA2,PTGDS,PTGES,RACGAP1,RBL1,RCN2,RNF128,RPL26,S100P,SCD,SCNN1B,SCNN1G,SDPR,SEPP1,SERPINA3,SERPINE2,SFRP1,SHH,SKA3,SLC13A3,SLC26A2,SLC2A1,SLC38A4,SLC39A10,SLCO2A1,SNAI1,SNRPC,SOX9,SPARC,SPARCL1,SPINK4,SPOCK1,SPP1,SST,SSTR5,STIL,STX1A,SULT1B1,SULT1C3,TAT,TFAP4,TGFB2,TGFBI,TGIF2,TIMP1,TK1,TMEM97,TNS4,TOP2A,TRPM6,TSC22D3,TSN,TXN,UBD,UBE2C,UCN2,UGT1A1,UGT1A6,UGT1A9 (includes others),VARS,VCAN,VIPR1,ZBTB16,ZIC2,ZNF343 | | | 574 (17) |  |
| E2F1 | transcription regulator | Activated | 1.72E-30 | | ABCG2,ATAD2,AURKA,AURKB,BBC3,BIRC5,BMP4,BRCA1,CA2,CCNA2,CCNB1,CCNB2,CCND1,CCND2,CCNE1,CCT2,CCT4,CD44,CDC20,CDC25A,CDC25B,CDC45,CDC6,CDCA4,CDK1,CDK2,CDK4,CHEK1,CITED2,CLIC1,CSE1L,DBF4,DNMT1,DUSP1,DUSP10,DUSP4,E2F1,ECT2,EIF3I,EYA2,EZH2,FAM60A,FANCD2,FEN1,FGFR2,FHL1,FOS,FOXM1,GINS1,HELLS,HIST1H2BJ,HMGA1,HMGB2,HSPA8,HSPD1,HSPE1,IGF1,JAG2,KCNA3,KIAA0101,KIF23,MAD2L1,MCM10,MCM2,MCM3,MCM4,MCM6,MCM7,MMP1,MMP3,MSH2,MT1G,MTHFD1,MUC4,MYBL2,MYC,NCL,NDC80,NUSAP1,ORC1,PA2G4,PCNA,PDCD5,PDX1,POLA1,POLD1,PRKDC,PRPS2,PSAT1,RACGAP1,RAD54L,RAN,RANBP1,RBL1,RFC2,RFC3,RFC4,RRM1,RRM2,RSL1D1,RUVBL1,SLC3A2,SMC4,SNRPC,STMN1,TK1,TNFSF9,TOP2A,TRAP1,TRIM28,TXNRD1,VRK1 | | | 397 (15) |  |
| CCND1 | transcription regulator | Activated | 1.13E-29 | | ASPM,ATAD2,AURKA,AZGP1,BIRC5,BRCA1,BRIP1,C7,CASC5,CCNA2,CCND1,CCND2,CCNE1,CD44,CDC45,CDC6,CDCA2,CDCA7,CDK2,CDK4,CDK6,CDKN2B,CENPF,CENPH,CENPK,CENPN,CEP55,CLSPN,COL1A1,COL27A1,COL5A2,DDIAS,DEPDC1,DKK2,DNMT1,DTL,E2F1,E2F7,EIF5A2,ENPP2,EREG,ESCO2,FAM83D,FOXM1,GAS2L3,HJURP,KIAA0101,KIAA1524,KIF11,KIF20A,KIF20B,KIF2C,KIF4A,KLK3,MCM10,MCM4,MCM7,MELK,MMP3,MORC4,MYC,NCAPH,PBLD,PCNA,PDCD4,PMEPA1,PSRC1,RACGAP1,RBL1,RBMS3,RMI2,RRM2,SELENBP1,SI,SLC4A4,SOX4,SPP1,ST6GALNAC1,STXBP1,TP53INP2,TPX2,TRAIP,TRIP13,ZNF367 | | | 419 (13) |  |
| CTNNB1 | transcription regulator | Activated | 2.82E-29 | | ABCD2,AGRN,ALCAM,ASCL2,AXIN2,BBC3,BCAP31,BCL2L1,BGN,BHLHE40,BIRC5,BMP2,BMP4,BMP7,CACNA1G,CCNA2,CCND1,CCND2,CCNE1,CD44,CDH11,CDK7,CDKN2B,CELSR1,CENPM,CFD,CHGA,CLDN2,CLDN5,CNN2,COL1A1,COL27A1,COL4A1,COL4A5,COL4A6,CTHRC1,CXCL12,CXCL8,CYP19A1,DIAPH3,DIO2,DPEP1,EDIL3,EDN3,EPHB2,EPHB3,EPHB4,ETV4,EVX1,EYA2,F13A1,FEN1,FGD2,FGF9,FN1,FOS,FOSL1,FOXQ1,FZD3,GAD1,GHR,GNAO1,GPX2,HAPLN1,HDGF,HHIP,HILPDA,HSD17B2,HSPE1,HTRA1,IFITM1,IGF2,IGJ,IRF4,ITGAL,KIF23,KIFC1,KLK3,KRT7,LAMC2,LEF1,LGR5,LY6E,ME1,MME,MMP1,MMP14,MMP3,MMP7,MPEG1,MPZ,MSX2,MYC,MYH3,NCAM1,NGFR,NKD1,NPHS2,NRG1,PCNA,PDE1C,PHLDA2,PLAU,PLS3,PMCH,QPCT,RCN1,SCD,SERPINA3,SERPINE2,SFRP1,SFRP4,SHH,SIM2,SLC12A2,SLC26A2,SMS,SNAI1,SOX11,SOX4,SOX9,SPP1,STXBP1,SYNM,TBX20,TDGF1,TIMP1,TWIST1,UBQLN4,VCAN,WISP1 | | | 579 (18) |  |
| ERBB2 | kinase | Activated | 6.46E-29 | | ABCG2,ADAM12,ALDOC,ASPM,AURKA,BCL2L1,BHLHE40,BIRC5,BMP7,BRIP1,BUB1,BUB1B,CCNA2,CCNB1,CCNB2,CCND1,CCND2,CCNE1,CD36,CDC20,CDC25A,CDH11,CDH3,CDK1,CDK6,CDKN2B,CDKN3,CDT1,CENPA,CENPE,CENPF,CHEK1,CKS2,COL1A1,COL3A1,COL4A1,COL5A2,COL7A1,CXCL10,CXCL12,CXCL8,DPEP1,EIF4EBP1,EPHB2,EREG,ESPL1,ESR1,ETV1,ETV4,ETV5,FASN,FEN1,FGF9,FN1,FOS,FSCN1,GHR,GINS1,GLO1,GTF3A,HMGB2,HTRA1,IGF2,IGJ,ITGA2,JAG2,JAM2,KLF4,KLK3,KRT7,LAMC2,LUM,MFAP2,MGAT5,MIF,MKI67,MME,MMP1,MMP11,MMP14,MMP3,MYBL2,MYC,NCAPD2,NCAPG,NDC80,NEK2,NOTCH3,NOX4,NRG1,PCNA,PDCD4,PDGFD,PDK4,PGK1,PHLDA2,PLAC8,PLAU,PMEPA1,POLD1,POLE2,PRC1,PRDX2,PTGES,RAB31,RAD51AP1,RFC4,RRM2,S100A6,S100P,SDPR,SERPINA3,SFRP1,SKP2,SLC2A1,SMC2,SNAI1,SOX4,SP5,SPAG5,SPARC,SPARCL1,SPINK4,SPOCK1,SQLE,TFAP2C,TGFBI,TGIF1,THY1,TOP2A,TP53RK,TPD52L1,UBE2C,UGT1A6,VCAN,WNT5B,ZWINT | | | 654 (23) |  |
| Vegf | group | Activated | 8.22E-29 | | ACAN,ADAMTS1,ANPEP,APLN,AURKA,AURKB,BIRC5,BMP2,BMP7,BUB1,BUB1B,CA2,CCNF,CD44,CD46,CDC20,CDC25A,CDC25B,CDC45,CDC6,CDK1,CDK2,CDKN3,CELSR1,CENPF,CHI3L1,CLDN1,CNN1,CNTFR,CSE1L,CXCL1,CXCL12,CXCL8,DBF4,DKC1,DUSP4,ENPP2,ESM1,FANCG,FN1,FOSB,FOSL1,FOXM1,GPR56,GPSM2,GRB10,HDC,HELLS,HMGCS2,HMMR,HOMER1,HOPX,IL11,INHBA,ITGA2,ITGB8,JAM2,KIF11,KIF15,KIF20B,KIF2C,LEF1,LPAR1,LRP8,LYVE1,MAD2L1,MCM2,MELK,MET,MFSD4,MIF,MKI67,MT1G,MYC,NDC80,NEK2,NOLC1,NOP2,NR5A2,NRG1,OXTR,P2RY14,PHLDA1,PHLDA2,PIM2,PKMYT1,PLAU,PLK4,PMCH,PRC1,PRKCB,RCC1,SFRP1,SHISA2,SKP2,SLC7A1,SMC2,STIL,TACSTD2,TIMP1,TPX2,TRAIP,TRIP13,TTK,UBE2C,XDH | | | 705 (24) |  |
